# Supplementary material for: Third delay in care of critically ill patients: a qualitative investigation of public hospitals in Kenya
Source: BMJ Open. 2024 Jan 4;14(1):e072341. doi: 10.1136/bmjopen-2023-072341 (PMC10773318; doi:10.1136/bmjopen-2023-072341)
Supplement: Supplementary data [file bmjopen-2023-072341supp001.pdf]

APPENDICES

Appendix 1: EECC checklists

PANEL 2. The Hospital Readiness Requirements for Essential Emergency and Critical Care

IDENTIFICATION OF CRITICAL ILLNESS

The following items are required for a hospital to be ready for the identification of critically ill patients:

CATEGORY	ITEM
1.1. EQUIPMENT	1.1.1 Clock with second hand<br>1.1.2 Pulse oximeter & probe<br>1.1.3 Blood pressure measuring equipment (eg. sphygmomanometer with a stethoscope)<br>1.1.4 Blood pressure cuffs of different paediatric and adult sizes<br>1.1.5 Light source (lamp or flashlight)<br>1.1.6 Thermometer
1.2 CONSUMABLES	1.2.1 Soap or hand disinfectant<br>1.2.2 Examination gloves
1.3 DRUGS	None
1.4 HUMAN RESOURCES	1.4.1 Health workers with the ability to identify critical illness 24h/day
1.5 TRAINING	1.5.1 The health workers are trained in the identification of critical illness
1.6 ROUTINES	1.6.1 Routines for the identification of critical illness
1.7 GUIDELINES	1.7.1 Guidelines for the identification of critical illness
1.8 INFRASTRUCTURE	1.8.1 Designated triage area (area for the identification of critical illness) in the Out-Patient Department or Emergency Unit (area of the hospital where patients arrive)<br>1.8.2 Running water

PANEL 2 continued ...

CARE OF CRITICAL ILLNESS

The following items are required for a hospital to be ready to provide the care of critically ill patients:

CATEGORY	ITEM
2.1 EQUIPMENT	2.1.1 Suction machine (electric or manual)<br>2.1.3 Oxygen supply 24h/day (cylinder, concentrator (with electricity supply) or piped oxygen)<br>2.1.4 Flow meter (if using cylinder or piped oxygen)<br>2.1.5 Leak-free connectors from oxygen source to tubing<br>2.1.6 Bag Valve Mask (resuscitator) – neonatal, paediatric and adult sizes<br>2.1.7 Sharps disposal container<br>2.1.8 External heat source
2.2 CONSUMABLES	2.2.1 Suction catheters of paediatric and adult sizes<br>2.2.2 Guedel airways of paediatric and adult sizes<br>2.2.3 Pillows<br>2.2.4 Oxygen tubing<br>2.2.5 Oxygen nasal prongs<br>2.2.6 Oxygen face masks of paediatric and adult sizes<br>2.2.7 Oxygen face masks with reservoir bags of paediatric and adult sizes<br>2.2.8 Masks for Bag Valve Mask (resuscitator) – neonatal, paediatric and adult sizes<br>2.2.9 Compression bandages<br>2.2.10 Plasters or tape<br>2.2.11 Gauze<br>2.2.12 Intravenous cannulas of paediatric and adult sizes<br>2.2.13 Intravenous giving sets<br>2.2.14 Skin disinfectant for cannulation<br>2.2.15 Syringes<br>2.2.16 Nutrition<br>2.2.17 Nasogastric tubes<br>2.2.18 Lubricant for nasogastric tube insertion<br>2.2.19 Intramuscular needles<br>2.2.20 Intraosseous cannulas of different sizes<br>2.2.21 Blankets<br>2.2.22 Facemasks for Infection Prevention and Control<br>2.2.23 Aprons or gowns<br>2.2.24 Charts/notes for documentation<br>2.2.25 Pens
2.3 DRUGS	2.3.1 Oral rehydration solution<br>2.3.2 Intravenous crystalloid fluids (eg. normal saline or Ringer's Lactate)<br>2.3.3 Intravenous dextrose fluid (eg. 5%, 10% or 50%)<br>2.3.4 Oxytocin<br>2.3.5 Adrenaline<br>2.3.6 Appropriate antibiotics<br>2.3.7 Diazepam<br>2.3.8 Magnesium sulphate<br>2.3.9 Paracetamol<br>2.3.10 Local anaesthetic (eg. 2% lignocaine) (eg. for intraosseous cannulation)
2.4 HUMAN RESOURCES	2.4.1 Health workers with the ability to care for critically ill patients 24hrs/day<br>2.4.2 Senior health worker who can be called to assist with the care of critically ill patients 24hrs/day

PANEL 2 continued ...

2.5 TRAINING	2.5.1 The health workers are trained in the care of critically ill patients
2.6 ROUTINES	2.6.1 Routines for managing critically ill patients<br>2.6.2 Routine for the provision of EECC without taking into account patients' ability to pay<br>2.6.3 Routines for who and how to call to seek senior help 24hrs/day, 7 days/week<br>2.6.4 Routines for integrating EECC with other care including the definitive care of the underlying condition (eg. use of condition-specific guidelines)
2.7 GUIDELINES	2.7.1 Guidelines for the essential care of critically ill patients
2.8 INFRASTRUCTURE	2.8.1 Designated space for the care of critically ill patients (eg. a bay, ward, high dependency unit)<br>2.8.2 Areas for separating and managing patients with a suspected or confirmed contagious disease from those without

PANEL 3. The essential diagnosis-specific care for critically ill patients with COVID-19

CLINICAL PROCESSES

1. The Essential Emergency and Critical Care (EECC) clinical processes as specified for all critical illnesses
2. Personal Protective Equipment (PPE) that is appropriate for COVID-19 as part of Infection, Prevention and Control (IPC)
3. Monitoring oxygen saturation using pulse oximetry at least every 6 hours, unless otherwise prescribed
4. Intermittent prone positioning
5. Low molecular weight heparin or other anticoagulant
6. Corticosteroid
7. Antibiotics in patients with suspected bacterial superinfection

HOSPITAL READINESS REQUIREMENTS

Critically ill patients with COVID-19 require the same hospital readiness for EECC as other critically ill patients. For the provision of the essential diagnosis specific care of critically ill patients with COVID-19, the following additional items are required:

CATEGORY	ITEM
3.1 EQUIPMENT	None
3.2 CONSUMMABLES	3.2.1 Facemasks appropriate for COVID-19 (eg. N95)<br>3.2.2 Eye protection or face shields
3.3 DRUGS	3.3.1 Low-molecular weight heparin (eg. enoxaparin or dalteparin) or other anticoagulant<br>3.3.2 Corticosteroid (eg. dexamethasone)
3.4 HUMAN RSOURCE	3.4.1 Health workers with the ability to care for critically ill COVID-19 patients 24hrs/day
3.5 TRAINING	3.5.1 The health workers are trained in essential care of critically ill COVID-19 patients
3.6 ROUTINES	3.6.1 Routines for care of critically ill COVID-19 patients
3.7 GUIDELINES	3.7.1 Guidelines for essential care of critically ill COVID-19 patients
3.8 INFRASTRUCTURE	3.8.1 Areas for separating and managing patients with suspected or confirmed COVID-19 from those without

Appendix 2: The combined in-depth interview guides (Guide 1 – frontline healthcare workers; Guide 2 – administrative healthcare workers)

2a) GUIDE 1: Interview Guides for Frontline Healthcare Workers

PART 1: Consent for recording

Before we begin, please can you confirm that you consent to having this interview recorded in line with the information sheet shared? Everything you say is confidential, the recorded data will not be shared with anyone outside the research team and the data will be anonymized.

PART 2: Introduction and basic information

Thank you for agreeing to participate in this research. Before we begin, I would just like to emphasize again that this is not an inspection or an audit – and no information specific to this hospital will be fed back to your managers. We are just interested in understanding more about the provision of care for critically ill patients in hospitals. So please feel at ease to be as open as possible with your responses.

**Date for interview**	**Day** [ ] [ ] **Month** [ ] [ ] **Year** [ ] [ ] [ ] [ ]
**Interviewer name**	
**County Name**	
**CODE** E.g. HCW_KE_H1_001_01	

GENERAL INFORMATION ABOUT RESPONDENT

**Gender**	1) Male … 01<br>2) Female… 02
**Age**	
**Job Title**	
**Name of hospital**	
**Name of department**	
**Highest educational qualification achieved?**	
**What kind of medical training do you have?**	
**How many years have you been qualified for this post?**	
**How many years have you been working at this hospital?**	

NOTE TO INTERVIEWER: PROVIDE OUR DEFINITION OF CRITICAL ILLNESS AND EXPLAIN THAT THE INTERVIEW QUESTIONS ARE BASED ON THIS DEFINITION.s

Critical Illness is a state of ill health with vital organ dysfunction, a high risk of imminent death if action is not taken and the potential for reversibility: so, we mean ***all the very sick patients who need immediate care*** *(and not just the patients in intensive care units or other critical care units).*

- Where are critically ill patients (children, pregnant women, medical adults, surgical adults) cared for in your hospital?
  Probes/clarification if they answer "ICU" or something else. Are critically ill patients cared for:
    - *In the OPD?*
    - *In the Emergency Dept?*
    - *In the general wards?*
    - *Anywhere else in the hospital?*
- How are you involved in the care of critically ill patients?
  Probe:
    - *in what capacity?*

- *In which department?*
- *For how long?*

PART 3: SCENARIO-BASED QUESTIONS

I will now ask a series of questions based on patient scenarios. This is not a test, I am just interested in the systems in your hospital and department. Please answer these questions based on what happens in your hospital, not what should happen based on your clinical training or in textbooks.

3.1 TRIAGE AND ADMISSION

NOTE TO INTERVIEWER: YOU MIGHT SKIP THROUGH RAPIDLY IF THE INTERVIEWEE IS NOT VERY FAMILIAR WITH THIS PROCESS.

Assume a new adult patient arrives at your hospital outpatient department/emergency department (as applicable) with symptoms of severe breathing difficulties.

- Can you describe what happens to this patient on arrival at your hospital?
  Probe:
  - *Who is the first person to help or provide care on arrival? Are they medically trained? Are they emergency medicine trained?*
  - *Registration system (paperwork, payments)?*
  - *Are there delays? Why?*
- How would the severity of this patient's condition be assessed to identify if he/she is critically ill? How does this work?
  Probe:
  - *Who does this?*
  - *Are triage categories used (mention which ones they use)/understood?*
  - Do you look for or screen people who might have signs associated with contagious diseases?
  - *Are Vital sign checked? Which ones?*
- Who is in-charge of triage at the outpatient department? How does this change over shifts?
  Probe:
  - *Night shifts versus day shift*
  - *How are your staff communicating with each other in this department? Are there differences in how staff members communicate with each other?*
- What care do you provide for critically ill patients in the OPD/emergency department in your hospital?
  Probe:
  - *If they become unconscious, what do you do? If they get shock or low blood pressure, what do you do? If their airway becomes unclear, what do you do?*
  - *Where you trained on the approach you use?*
  - *Is there a written guideline or protocol that you follow for unconscious patients?*
- *Is oxygen available in the OPD/emergency department?*
  Probe:
  - *In what form? Concentrators? Cylinders? Piped to bedside?*
  - *Is the supply reliable? How often are there shortages (e.g. weekly).*
  - *How many days in a week do you have patients who need oxygen but you aren't able to provide it?*
  - *How do you decide if the oxygen supply is enough?*
  - *If oxygen runs out or components are missing, what process do you use to report/resolve this?*
  - *If not: why not? How do you manage?*
- Where would this patient be admitted and how would they get there?
  Probe:
  - *Clarify process- straight to ward for admission or via resuscitation room/other?*
  - *Do patients have to wait along the way? And why?*
  - *Does the system for handover and communication with the receiving department/unit/ward work? Why not?*
- Overall, what are the biggest challenges you face during triage and admission of critically ill patients in your hospital?

Probe:

- *Reflect on what has already been discussed: equipment, supplies, communication, drugs, guidelines, routines, training, staffing issues?*

3.2 MANAGEMENT, MONITORING AND ESCALATION OF CARE

NOTE TO INTERVIEWER: YOU MIGHT SKIP THROUGH RAPIDLY OR COMPLETELY DEPENDING ON POSITION OF INTERVIEWEE AND HOW FAMILIAR HE/SHE IS WITH THIS PROCESS. USE YOUR JUDGMENT TO ADJUST QUESTIONS IF REQUIRED, BASED ON WHERE THE INTERVIEWEE WORKS.

*Now, consider the ward or unit where you work the most. Assume this **critically ill** patient has been admitted to your ward/unit with a clinical syndrome consistent with a pneumonia*

- Can you briefly describe the patient handover process when a critically ill patient is admitted at your ward/unit?
  Probe:
  - *Who is involved?*
  - *How do staff communicate with each other both in the department and with other departments?*
  - *Does it work? Why not?*

- After admission, how are critically ill patients checked or monitored for deterioration or improvement?
  Probe:
  - *What monitoring is done? How would you know a patient is deteriorating?*
  - *Are vital signs checked? Which ones?*
  - *How often is this monitoring done? By whom?*
  - *How is this documented and communicated to others on duty?*
  - *Where in your department are they monitored?*
  - *Is someone responsible/in- charge? Who?*
  - Are patients consulted or their opinions asked when the team makes a management decision?
- Is medical oxygen always available in your ward/unit for such patients?
  Probe:
  - *In what form? Concentrators? Cylinders? Piped to bedside?*
  - *Is the supply reliable? How often are there shortages (e.g. weekly). Is it available on all wards?*
  - *If not: why not? How do you manage?*

- What care do you provide for critically ill patients in your ward/unit?
  Probe: if they become unconscious, what do you do? If they get shock or low blood pressure, what do you do? If their airway becomes unclear, what do you do? Are there any written guidelines you follow for this? If so do they work well and how?
- What training have you received to monitor and manage such a critically ill patient?
  Probe:
  - *On the wards, are there any guidelines you follow to monitor and manage patients?*
  - *Who makes decisions about how a patient is monitored in your department?*
  - *Are you confident in being able to identify deterioration? Why/why not?*
  - *Are you confident in being able to care for such patients? Why/why not?*
- What options for escalation of care are available to you, if the patient deteriorates?
  Probe:
  - *CPAP*
  - *High-flow nasal oxygen*
  - *Intubation and mechanical ventilation*
  - *Admission to HDU or ICU*
  - *Referral to another hospital (which hospital?)*
  - *Is the capacity sufficient? Why not?*
- Is there a framework or guideline used in your ward or department to decide when to stop certain treatments?" If so is it helpful?
- Are senior staff available to advise/help for management of critically ill patients?

Probe:

- *Which cadres and types of staff?*
- *Is there a system for alerting them? Please describe.*
- *How effective is this system? Is anyone in-charge of checking it is working?*
- *Differences between night and day shifts?*

- What are the biggest challenges you face when monitoring and managing critically ill patients admitted to your ward/unit?
  Probe: Reflect on what has already been discussed: equipment, supplies, communication, drugs, guidelines, routines, training, staffing issues?

PART 4: GENERAL QUESTIONS

NOTE TO INTERVIEWER: YOU MIGHT NOT HAVE TIME TO DO ALL OF THIS. DO NOT PROBE IF TIME IS VERY LIMITED.

We know that some hospitals have experienced increased cases of patients with respiratory illness in the past 1 year due to COVID-19, and we are interested in how hospitals have managed.

- Did your hospital face any surge in patients with respiratory illness in the past one year due to COVID-19?
- How has care for critically ill patients changed in your facility since the COVID-19 pandemic?
  Probe:
  - *New emergency department or unit?*
  - *New HDU/ICU or increased capacity? Is it in use? Are there any plans to improve its use if its not fully operational? What was the previous capacity?*
  - *Increased number of staff working in critical care, emergency care, anaesthesia? Where have they been placed?*
  - *New equipment or supplies for emergency or critical care? Are they in use? What was you previous equipment capacity on the wards and in OPD/emergency department?*
  - *Increased oxygen capacity? E.g did you get a new oxygen plant? Is it in use?*
  - *Changed processes for identifying and managing patients who need higher levels of care? Has this been beneficial?*
- Have there been changes made to how things are done in this facility or your department in the past (I.e note keeping, handover, handling equipment etc)?
  - If so who started it? How did people receive this change?
  - Why was it successful or not successful?
  -
- Has your hospital received any support (funding/donations in kind/technical support) to strengthen management of the critically ill due to COVID-19? or is any support expected in the coming months?
  Probe:
  - *What support and from whom?*
  - *Do you think it has helped/not helped? Why? (Equipment, drugs, additional staff, supplies, training)*
  - *If no: has this been challenging?*
  - *How did your hospital cope e.g. repurpose staff, longer shifts, borrow equipment from other departments?*
- Compared to pre COVID-19 pandemic, do you think your hospital is more ready to identify, care for and monitor critically ill patients?
  Probe:
  - *If yes/no, how and why?*
- Are there any important issues that still need to be fixed, which you haven't already mentioned?
  Probe:
  - *Equipment, supplies, staff, training, routines/guidelines, communication, systems*
- Finally, if we want to run a program to improve the management of all critically ill patients, what would you recommend that we do?
  Probe:
  - *What additional support would have the most impact/be most helpful?*
  - *How would this improve the care of critically ill patients*

Thank you very much for your time.

2b) GUIDE 2: Interview Guide for Administrative Healthcare Workers- Kenya

PART 1: Consent for recording

Before we begin, please can you confirm that you consent to having this interview recorded in line with the information sheet shared? Everything you say is confidential, the recorded data will not be shared with anyone outside the research team and the data will be anonymized.

PART 2: Introduction and basic information

Thank you for agreeing to participate in this research. Before we begin, I would just like to emphasize again that this is not an inspection or an audit – and no information specific to this hospital will be fed back to your managers. We are just interested in understanding more about the provision of care for critically ill patients in hospitals. So please feel at ease to be as open as possible with your responses.

**Date for interview**	**Day** [ ] [ ] **Month** [ ] [ ] **Year** [ ] [ ] [ ] [ ]
**Interviewer name**	
**County Name**	
**CODE** E.g. HCW_KE_H1_001_01	

GENERAL INFORMATION ABOUT RESPONDENT

**Gender**	3) Male … 01<br>4) Female… 02
**Age**	
**Job Title**	
**Name of hospital**	
**Name of department**	
**Highest educational qualification achieved?**	
**What kind of medical training do you have?**	
**How many years have you been qualified for this post?**	
**How many years have you been working at this hospital?**	

NOTE TO INTERVIEWER: PROVIDE OUR DEFINITION OF CRITICAL ILLNESS AND EXPLAIN THAT THE INTERVIEW QUESTIONS ARE BASED ON THIS DEFINITION.

*Critical Illness is a state of ill health with vital organ dysfunction, a high risk of imminent death if action is not taken and the potential for reversibility: so, we mean **all the very sick patients who need immediate care** (and not just the patients in intensive care units or other critical care units).*

PART 3: SCENARIO-BASED QUESTIONS

I will now ask a series of questions based on patient scenarios. This is not a test, I am just interested in the systems in your hospital and department. Please answer these questions based on what happens in your hospital, not what should happen based on your clinical training or in textbooks.

3.1 TRIAGE AND ADMISSION

NOTE TO INTERVIEWER: YOU MIGHT SKIP THROUGH RAPIDLY IF THE INTERVIEWEE IS NOT VERY FAMILIAR WITH THIS PROCESS.

Assume a new adult patient arrives at your hospital outpatient department/emergency department (as applicable) with symptoms of severe breathing difficulties.

- Can you describe what happens to this patient on arrival at your hospital?
  Probe:
  - *Who is the first person to help or provide care on arrival? Are they medically trained? Are they emergency medicine trained?*
  - *Registration system (paperwork, payments)? How do you decide which payments must be made upfront?*
- Who decides which systems are used to identify the severity of this patient's condition if he/she is critically ill? How does this work?
  Probe:
  - *Are there staff trainings in this department on systems to manage critically unwell patients?*
  - *Who is responsible for training staff on which systems to use?*
  - *Are anaesthetists or critical care staff involved in training staff in your department on critical illness management?*
  - *Who ensures staff have the equipment they need to use these systems?*
- Who is in-charge of triage at the outpatient department? How does this change over shifts?
  Probe:
  - *Night shifts versus day shift*
  - *Who decides how many staff members are needed for each shift?*
- *Is oxygen available in the OPD/emergency department?*
  Probe:
  - *In what form? Concentrators? Cylinders? Piped to bedside?*
  - *Is the supply reliable? How often are there shortages (e.g. weekly).*
  - *Who is responsible for monitoring oxygen supply? Who maintains the oxygen delivery systems?*
- If this patient was admitted to a ward, how would staff communicate between departments?
  Probe:
  - *Who is responsible for setting up and maintaining communication systems?*

3.2 MANAGEMENT, MONITORING AND ESCALATION OF CARE

NOTE TO INTERVIEWER: YOU MIGHT SKIP THROUGH RAPIDLY OR COMPLETELY DEPENDING ON POSITION OF INTERVIEWEE AND HOW FAMILIAR HE/SHE IS WITH THIS PROCESS. USE YOUR JUDGMENT TO ADJUST QUESTIONS IF REQUIRED, BASED ON WHERE THE INTERVIEWEE WORKS.

*Now, consider the ward or unit where you work the most. Assume this **critically ill** patient has been admitted to your ward/unit with a clinical syndrome consistent with pneumonia*

- After admission, how are critically ill patients checked or monitored for deterioration or improvement?
  Probe:
  - *What monitoring is done? How would staff know a patient is deteriorating?*
  - *Are vital signs checked? Which?*
  - *How often is this monitoring done? By whom?*
  - *How is this documented and communicated to others on duty?*
  - *Where in your department are they monitored?*
  - *Is someone responsible/in- charge? Who?*
- Is medical oxygen always available in your ward/unit for such patients?
  Probe:
  - *In what form? Concentrators? Cylinders? Piped to bedside?*
  - *Is the supply reliable? How often are there shortages (e.g. weekly). Is it available in all wards?*
  - *Who is responsible for monitoring oxygen supply? Who maintains the oxygen delivery systems?*

- What training have staff received to monitor and manage such a critically ill patient?
  Probe:
  - *When did they receive this training?*
  - *Do they receive refresher sessions? How often?*
  - *Who decides which staff should undergo training?*
- What options for escalation of care are available to you, if the patient deteriorates?
  Probe:
  - *Is there a guideline or SOP staff use to escalate care?*
  - *Who decides which guidelines are used?*
- Is there a framework or guideline used in your ward or department to decide when to stop certain treatments?"

PART 4: GENERAL QUESTIONS

NOTE TO INTERVIEWER: YOU MIGHT NOT HAVE TIME TO DO ALL OF THIS. DO NOT PROBE IF TIME IS VERY LIMITED.

We know that some hospitals have experienced increased cases of patients with respiratory illness in the past 1 year due to COVID-19, and we are interested in how hospitals have managed.

- Did your hospital face any surge in patients with respiratory illness in the past one year due to COVID-19?
- How has care for critically ill patients changed in your facility since the COVID-19 pandemic?
  Probe:
  - *New emergency department or unit?*
  - *New HDU/ICU or increased capacity? Is it in use? Are there any plans to improve its use if its not fully operational? What was the previous capacity?*
  - *Increased number of staff working in critical care, emergency care, anaesthesia? Where have they been placed?*
  - *New equipment or supplies for emergency or critical care? Are they in use? What was you previous equipment capacity on the wards and in OPD/emergency department?*
  - *Increased oxygen capacity? E.g did you get a new oxygen plant? Is it in use?*
  - *Changed processes for identifying and managing patients who need higher levels of care? Has this been beneficial?*
- Have there been changes made to how things are done in this facility or your department in the past (I.e note keeping, handover, handling equipment etc)?
  - If so who started it? How did people receive this change?
  - Why was it successful or not successful?
- Has your hospital received any support (funding/donations in kind/technical support) to strengthen management of the critically ill due to COVID-19? or is any support expected in the coming months?
  Probe:
  - *What support and from whom?*
  - *Do you think it has helped/not helped? Why? (Equipment, drugs, additional staff, supplies, training)*
  - *If no: has this been challenging?*
  - *How did your hospital cope e.g. repurpose staff, longer shifts, borrow equipment from other departments?*
- Finally, if we want to run a program to improve the management of all critically ill patients, what would you recommend that we do?
  Probe:
  - *What additional support would have the most impact/be most helpful?*
  - *How would this improve the care of critically ill patients?*

Thank you very much for your time.

Appendix 3: Process maps

Appendix 3a: Process map – Facility 1

Entry and triage

- The hospital receives patients either as walk-ins/referrals
- The triage nurse sorts patients to their respective care points
- Preliminary vital signs are taken by the triage nurse

Casualty & Resuscitation

- The casualty room also doubles up as the resuscitation room
- The room has two examination couches and 5 beds
- Clinicians review patients hereupon arrival

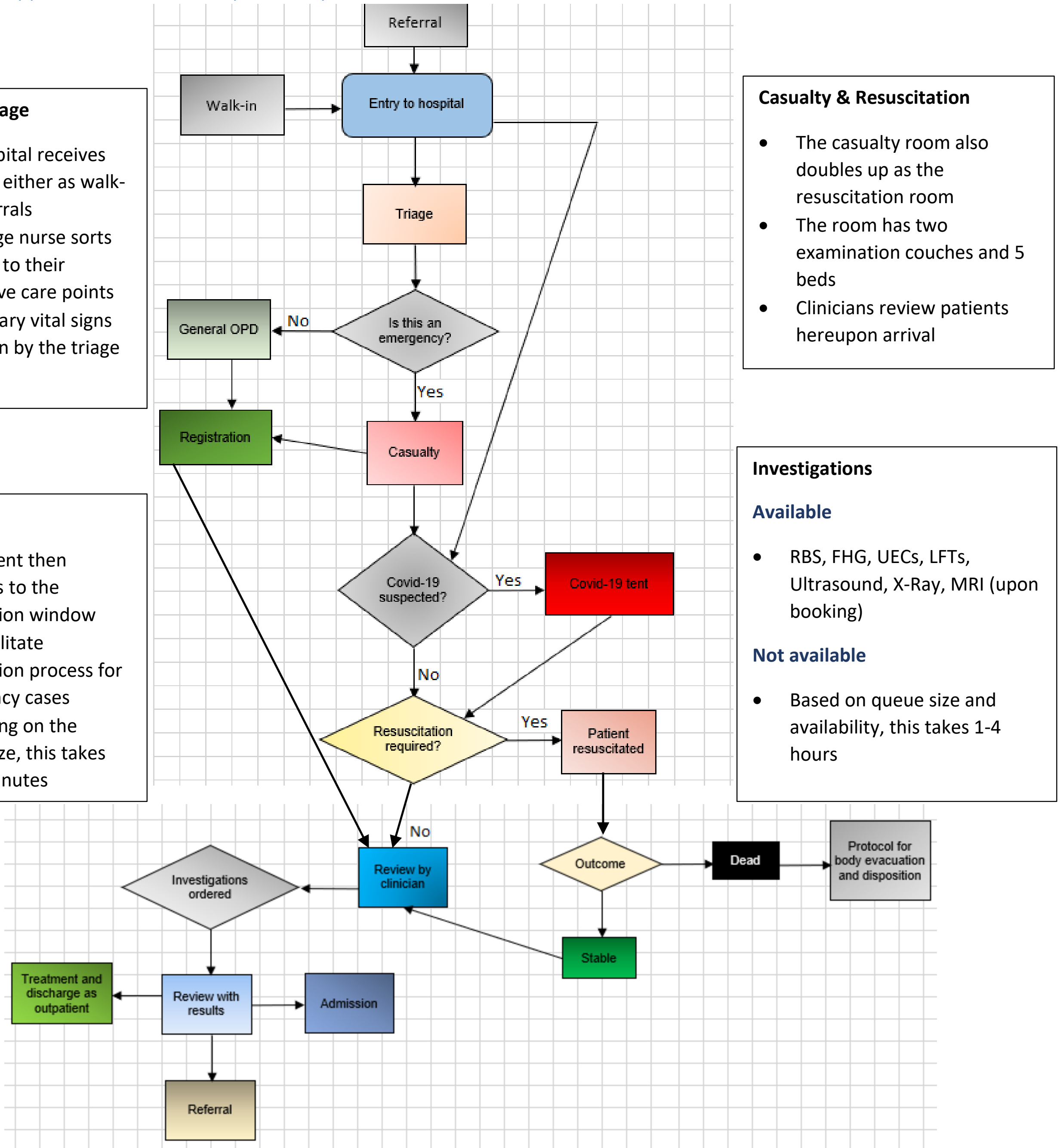

Registration

- The patient then proceeds to the registration window
- Staff facilitate registration process for emergency cases
- Depending on the queue size, this takes 10-45 minutes

Investigations

Available

- RBS, FHG, UECs, LFTs, Ultrasound, X-Ray, MRI (upon booking)

Not available

- Based on queue size and availability, this takes 1-4 hours

Consultation protocol

- Clinical Officer (+/- clinical officer interns) sees patient
- Clinical Officer calls Medical Officer/registrar
- MO/registrar calls consultant

Appendix 3b: Process map – Facility 3

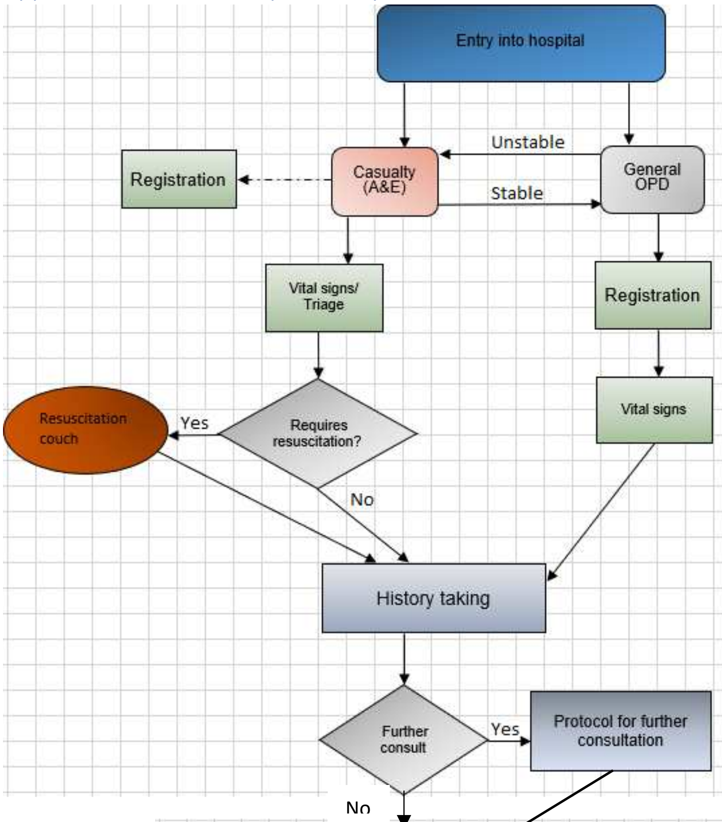

Registration

- Registration may go on during resuscitation/triage if the patient is accompanied by someone
- Takes place upon confirmation of payment/health cover
- The process takes 2-60 minutes

History taking

- History taking is done by a medical officer +/ a nurse. The MO (or MO + MO intern) and the nurse take patient history independently for their respective teams.
- Takes place upon confirmation of payment/health cover
- The process takes 2-60 minutes

Consultation protocol

- The Medical Officer (+/- Medical Officer Intern) sees the patient in the consultation cubicles
- The channel for consultation is Medical Officer (+/- Intern)-> Registrar -> Consultant

Investigations

- Blood works – FHG, LFTs, UECs, Coagulation profile, Blood gas analysis (BGA)
- Radiology – CT scan, X-ray, MRI, Ultrasound, Echocardiography
- Whole process takes 30 minutes to 3 hours

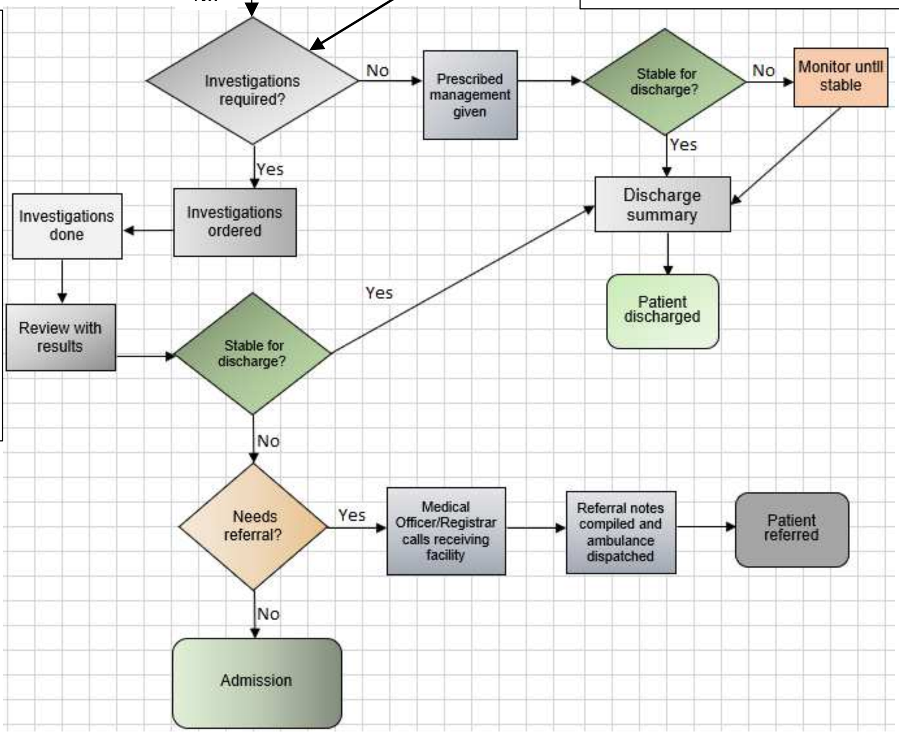

Appendix 4: Patient Journeys

4a) Journey of a patient presenting with severe abdominal pains (later diagnosed with ruptured ectopic pregnancy) at Facility 1.

Date: September 2, 2021

Narrator: OO, Assistant Research Officer – POETIC-COVID project

10:20 am: We have just received an adult female patient – she has been brought in [at the A&E] accompanied by two relatives. She is in obvious distress and reports symptoms of severe progressive abdominal pain. She has been put on a bed – the nurse and a clinician (Clinical Officer) are already with her. I'll keep you posted.

10:24 am: The vitals have been taken: SPO2 is 93% on room air, the heartrate is 90 b/min. Other parameters seem, okay? A thing to note is that this unit, the ER, has five couches, four oxygen ports and there are two bedside monitors that are operational. So, I'll keep you posted.

10:30 am: The nurse is fixing an IV line, together with the Clinical Officer.

10:34 am: The Clinical Officer has written a lab request for a blood test [full hemogram]. The nurse has drawn a blood sample to be taken to the lab. So, the patient is still in bed.

10:40 am: The nurse has repeated the vital signs; BP is 93/50mmHg. The nurse is putting up IV normal saline [500 ml] right now.

10:57 am: Status update: patient still in bed, IV fluid running. No other consultations made.

11:20 am: The first bottle of IV is over, the nurse has put up a second 500ml bottle of normal saline. The relatives have been asked to go make payments for the blood sample to be taken to the lab for processing. They are yet to make the payments.

11:30 am: The second IV is running well. We still await review. Notable is that the unit is favourably staffed, I can count five nurses in the shift – although one of them is the unit in-charge, but the other four are pretty much on the ground – also two Clinical Officers and one Medical Officer.

11:35 am: The relatives have gone to make payments, after which the blood sample will be taken to the lab.

12:04 pm: The relatives are back with the receipt of payment. So, any time now the sample will be taken to the lab.

12:21 pm: The second IV normal saline is still running. Vital signs have been repeated: BP is 98/59mmHg. Also, on assessment, the patient was noted to be pale. We still await the results from the lab.

12:45 pm: The Medical Officer has reviewed the patient and has requested for an abdominopelvic ultrasound to determine what could be the cause of her discomfort and symptoms. The IV is still running. Meanwhile, the relatives have been asked to go make payment for the radiological tests.

1:07 pm: The second IV fluid is still running. It is around the change-over time, new staff are coming in for the afternoon shift. They are sitting together, and a hand-over process is going on - the new staff are being briefed on the patients' conditions – so we hope that things are going to be swift and the patient is going to receive the care soonest possible.

1:14 pm: The patient is being wheeled to the radiology department for the abdominopelvic ultrasound. She's accompanied by her relatives. Payment to be made at the cashier's window next to the department.

1:15 pm: We are at the radiology department: there is a queue, so, the patient will have to wait outside on the wheelchair before she's seen. Payment is being made.

1:45 pm: One relative [patient's sister] has come back to the casualty [A&E]. Apparently, she has been told that the test imaging (abdominal ultrasound) cannot be done until a later time, maybe even tomorrow since the patient is required to have fasted before the radiological test. We are yet to establish if there's anything else that can be done.

1:52 pm: The doctor has instructed the patient's sister to communicate to the radiology department technicians that the test is urgent and that this patient is due for admission into the ward. We are hoping that the patient is going to get the test done soon. The patient is still waiting outside the radiology department on a wheelchair in the company of the other relative.

1:57 pm: The patient has been wheeled back to the casualty department, but the test has not been done because the radiology team want a clear communication from the casualty on the urgency of the test.

2:05 pm: The doctor has indicated [in writing] in the patient's file that the test request is URGENT, and it needs to be done as soon as possible. That communication is being relayed to the radiology through the file that is to be taken there by the patient's relatives. The patient is being wheeled back to the radiology department. We hope that the test is going to be done and the results to be out soon because this patient is supposed to be admitted.

2:15 pm: The patient has been wheeled back again into the A&E from the radiology department. The test has not been done. The radiology team holds that the abdominal ultrasound cannot be done right now because the patient is needed to have fasted so it will have to be done later. The doctor at the A&E has reviewed the initial request and ordered specifically for a pelvic ultrasound. The patient is to be taken for a pelvic ultrasound soon.

2:25 pm: The patient has been wheeled to the radiology department again, with the new request, so we await feedback. We hope that the test is going to be done so she can proceed to the next care point. They are to make payment for the new test and reverse the initial payment.

2:35 pm: Patient is in the waiting bay at the radiology department. She has been instructed to take some water (has a litre of water) to have a full bladder to aid the pelvic ultrasound. The patient's sister has gone to make the payment.

3:03 pm: The patient is still waiting outside. The waiting area seems packed; a lot of patients waiting to be seen. We are just hoping that the patient will be in soon.

3:37 pm: Status update: not much has moved, the patient is still waiting outside.

5:20 pm: Patient still waiting in the waiting bay. She is next in line to be seen, likely in the next ten or less minutes.

5:33 pm: The patient has just been wheeled into the radiology room.

5:50 pm: The patient has just left the radiology room. The results are expected to be out within the next 30 minutes, so they'll have to wait a little longer after she leaves the radiology department.

6:17 pm: Final update for Patient 1. The patient has been wheeled back to the casualty, awaiting review with the radiology results. This marks the end of my follow-up with this patient because of time but from

the look of things the doctor says the patient is to be admitted, although that is after review with results once the results are out.

Follow up call at 8:26 am - Friday September 3: The patient was admitted in the gynaecological ward at night around 10:30pm with a diagnosis of a ruptured ectopic pregnancy and was taken to theatre for urgent surgical intervention [exploratory laparotomy].

4b) An adult male patient brought in unconscious at Facility 2 (Case 2 in the main document).

Date: September 13, 2021

Narrator: OO, Assistant Research Officer – POETIC-COVID project.

12:06 pm: I am at Facility 2. The patient has just been brought in, carried by two relatives. He looks unconscious. The patient has been put on a chair in the ER. The ER has two rooms; A& B, each with 4 beds.

12:08 pm: The nurse and two EMTs are setting up to take vital signs. The random blood sugar (RBS) has just been taken and is 8.5 mmol/L. The patient is still unresponsive. Meanwhile, the relatives have been asked to go start the registration process.

12:10 pm: The vitals seem okay except for the SPO_2: the BP is 130/75mmHg, HR is 81b/min and SPO_2 is 85% on room air. The ER doesn't have piped oxygen, but we have two huge oxygen tanks with flowmeters attached (no flow splitters). Right now, the two cylinders are both in use. The room has two monitors with one SPO_2 probe attached to one of the monitors. The other SPO_2 probe is in use at the triage area.

12:19 pm: The patient has been taken to the other room, where a space has been created in a bed. The patient is yet to be reviewed; the relatives are not yet back from the registration office.

12:22 pm: The patient has been put onto a bed in the other room, but he's sharing the bed with another patient since the space is full. This room also has 4 beds that are currently all occupied. Two more patients are sitting in chairs with IV drips. This cubicle has one oxygen tank with a capacity of about 50.2L.

12:31 pm: The Medical Officer and two EMTs are with the patient. The EMTs are fixing an IV cannula and drawing a blood sample into two vacutainers.

12:35 pm: The blood samples have been drawn into the two sample containers. The Medical Officer has drawn up a lab request and handed it to the patient's relative to deliver to the lab upon making the payments. The patient is still in bed and awaits further review.

12:55 pm: A 500ml bottle of IV Ringer's Lactate has been set up and is running well. The patient is yet to be reviewed.

1:16 pm: The IV is over and the patient still awaits further review. The patient is still in distress. No further vital signs have been taken. We await the next course of steps. The ER is quite busy, and there's a queue outside of patients waiting to be seen. The two rooms in the ER are full as well, with patients sharing beds. We are just hoping that the patient is going to be reviewed soon.

1:25 pm: The patient has been noticed to be having convulsions. We still haven't had a concrete review because the doctor is quite held up and the patient's details have not been updated.

1:40 pm: The doctor has prescribed an anticonvulsant. The prescription has been handed to a relative to pick up the drug from the pharmacy upon payment. The unit is still very busy, with very sick patients being wheeled in.

2:41 pm: The medication has just been brought and will be administered any time now. The patient is still in bed in status quo; still unresponsive and having convulsions. The results are not yet out.

2:56 pm: The nurse has just given the injection of the anticonvulsant, so, right now we await that to take effect. We hope the patient will be reviewed soon; the lab results are still not out.

3:50 pm: The results are still not out, and the patient still awaits a review. No further vital observations have been taken.

4:15 pm: The patient hasn't been reviewed yet; the results are yet to be received back from the lab. No further vitals have been taken. The patient's condition hasn't improved much, but the convulsions are not as strong as they were before. This is the final update for today because of time.

4c) Journey of a patient who presented with symptoms of severe difficulty in breathing (Covid-19 patient) at Facility 4 (Case 3 in the main document).

Facility: Facility 4

Date: June 9, 2021

Narrator: OO, Assistant Research Officer – POETIC-COVID project.

0830h: Morning. This is OO, the ARO for the POETIC-COVID study. It is about 8:30 am, a patient has just been wheeled into the casualty [ER] with apparent symptoms of severe breathing difficulties. The patient is being wheeled onto a couch in the casualty. The Medical Officer is still not yet in. The doctor [Medical Officer] was here overnight, so he has stepped out to refresh before coming back. Upon coming back, we hope the patient will be reviewed and if at all there are any investigations to be taken the patient will be taken accordingly. That's all for now, we wait for the next steps.

0835h: Update for Patient 2, it is 8:35 am. The vitals have just been taken. It has been noted that the heartrate is a little unstable, ranging as low as from 50s to 60s but at times it's spiking, so not really stable. She's in obvious breathing difficulty [distress]. The SPO2 currently is reading 58% off oxygen. To be noted again is that the unit has one pulse oximeter that is operational. There is another one attached to the monitor but not working. The other patients…there is one other patient who needs constant pulse oximetry, but he has his own…the family came with a personal pulse oximeter, so I think for that he's sorted for now. That is allfor now, I'll keep you posted.

0900h: Update for Patient 2, it's 9 am. The doctor arrived at around 8:57 am. He has reviewed the patient as well as the others. He's suggesting an X-ray, so the relatives have gone to the registration window to open a file for the patient. So, we await that after which the prescribed test [X-ray] …she'll be wheeled in.

0915h: Patient 2, the time is 9:15 am. The patient has just been wheeled in for the X-ray. Being that she isamong the very first patients who reported to this casualty we are hoping that it won't take long, she's going to be attended to almost immediately. So, we hope that she'll be out in the next few minutes.

0937h: The patient has just left the X-ray department, awaiting review with results [results are not out yet]…so, the patient is back in the casualty. I'll keep you posted. It's now 9:37 am, the patient went in at 9:15 am and is already out. So, we'll follow up upon review of the results.

0950h: The patient has been put on oxygen, in bed at the casualty department awaiting the X-ray results. Not really connected to a monitor because the monitors are faulty, but the patient is on oxygen, so from time to time the vitals are being checked. I'll keep you posted.

1015h: The results are out…the X-ray results are out but apparently there has to be a Covid test done. TheRDT [Covid rapid test] is not available in that department so the patient has to be taken away again; a stone-throw away within the hospital for the test. The relatives are still following up with whoever is responsibleon how best that can be done, but otherwise, the patient is still on oxygen, we are awaiting a review of theX-ray results. There is a queue…there's one patient ahead so the Medical Officer is still with another patientbut once he's done, I think the patient will be reviewed with the X-ray results. However, we are still following up with the Covid team for the Covid test. I'll keep you posted.

1043h: Update for Patient 2, 10:43 am. The Covid team came to take samples for the rapid test from the casualty. The samples are being taken, so we await the next few minutes for the results to be out after

which it'll inform the direction of care; whether this patient is going to be admitted in the medical ward orthe Covid isolation ward. I'll keep you posted, that's the update for now.

1127h: Update for Patient 2, it is 11:27am. The test results for Covid-19 are out, she has been confirmed tobe Covid positive. So, the patient has been indicated to be admitted in the Covid isolation ward. Initially they had planned to admit her in the female medical ward, but now that the results are out, she's going tobe taken to the isolation unit. One thing about the isolation unit (I was already there a just a while ago), it has a capacity of six [6]. It has a total of 6 beds. At the moment four [4] of those are occupied, so we are hoping that this patient is going to get space. We are just hopeful that the space avails…we keep on waitingat the casualty. The patient is still on oxygen via face mask, the vitals-the SPO2 is okay. She's saturating on97% on oxygen, but the pulse rate was a little worrisome initially. It was going as low as 52, at one point even at 43 beats per minute but at the moment we cannot say that she's so badly off. She's still on oxygen, pulse rising to above 68. That's the update for now.

1140h: Update for Patient 2. The vitals have been taken again…still the SPO2 is ranging between 90-93% onoxygen. To be noted again is that there is no high-flow oxygen in this facility due to the oxygen supply [strained]. Again, in the casualty there is on oxygen port which is being used to give oxygen to this patient via mask. There's no non-rebreather mask in the department as at now but hopefully the SPO2 will rise, it is promising. Again, this unit is actually very full at this point, remember there is only one medical officer. The unit is full and other patients are actually complaining. Also there's another patient who is having similar issues [as Patient 2] and they [the medical team] were trying to locate a bed space for the patient…there has been a push-and-pull between the Medical Officer here and the destination hospital [FACILITY X], they have been arguing over phone and the Medical Officer seems frustrated here. Looks like he feels his hands are tied and the patients need help and they cannot be able to help but we are hoping that things ease up over the next few minutes.

1254h: Update for Patient 2, the time is 12:54 pm. It has been confirmed that there is bed space for this patient in the Covid isolation ward in this Facility. Her latest SP02 was 94%. So, we are waiting for a stretcherto be brought so that the patient can be wheeled there.

1304h: Update for Patient 2, the time is 1304h. The patient has just been put on the stretcher to be takento the Covid isolation ward…the patient is being admitted in the Covid isolation ward. The patient is to be taken there on portable oxygen in a cylinder. They're setting that up. I'll keep you posted once the patientlands in the Covid isolation ward. They have just left the casualty.

1319h: Update for Patient 2, the time is 1319h. The patient has just been transferred to the Covid isolation unit...patient has been admitted. To be noted is that this patient is currently saturating at 94% on oxygen but on admission [when she landed at the casualty] the patient was actually saturating as low as 17% - thatwas off oxygen. The patient has been maintained in the casualty on oxygen and has been transferred into the Covid isolation unit. As it stands the patient has been admitted into the isolation unit with Covid-19 Pneumonia. I'll halt there for now, that marks the end of my journey with

4d) An adult male patient brought in unconscious at Facility 2.

Facility 2
Date: September 13, 2021
Narrator: OO, Assistant Research Officer – POETIC-COVID project.

11:40 am: I am in the A&E department at Facility 2. The ER has two rooms: A & B, each with 4 beds. At the moment all eight beds are occupied. There's no piped oxygen in the department but I can count three oxygen cylinders with a capacity of 50.2L each. Right now, two of the cylinders are in use. Each cylinder has a flowmeter attached, but no flow splitters. The cylinders serve one patient at a time.

11:45 am: An adult male patient has been brought in, carried by two relatives, apparently unconscious.

11:48 am: The patient is in a chair (all 8 beds are currently occupied), still not responsive. The nurse and an assistant - looks like a trained Patient Attendant or an EMT – are attending to the patient. They are setting up to take the patient's vital signs. The SPO2 probe has just been put. The EMT has fixed an IV line.
The RBS is 3.4 mmol/L. A blood sample has been drawn, to be taken to the lab upon payment by the relatives. I'll keep you posted.

12:15 pm: The patient is still in a chair since the 4 beds in this cubicle are occupied. There is an adjacent cubicle but that as well is currently full. The nurse and an EMT are trying to set up an IV fluid, but they first have to settle the patient in a bed. They are trying to create a bed space by relocating another patient who has stabilized. Just to add, this department has 3 nurses, 1 Medical Officer and 2 Emergency Medical Technicians (EMTs). The EMT assist with wheeling the patients as well as fixing IV lines.

12:21 pm: The patient has been put onto a bed, but he's sharing the bed with another patient since the space is full. The nurse has just put up a 500 ml bottle of 5% IV Dextrose. The patient's relatives have been given the sample to deliver to the lab upon making payment at the laboratory department.

12:40 pm: The IV Dextrose is still running. No further review at this point. The department is quite busyand packed with patients. The results are not yet out from the lab.

12:48 pm: The patient is currently responsive; he is waking up. The IV Dextrose is still running, but still no further review yet.

1:15 pm: The IV fluid (5% Dextrose) is over. The patient is awake and looks stable. We still await review. The lab results are not out yet. The patient has been taken outside to wait on the bench.

1:50 pm: The patient is stable and has been asked to wait outside on the bench in the waiting bay so as tocreate a bed space for another patient. The results are not ready from the lab. The patient will be reviewed once the results are out.

4e) An adult female patient brought in by social worker with symptoms of respiratory distress at Facility 2. Observation was concurrent with 4b above.

Facility: Facility 2
Date: September 13, 2021
Narrator: OO

12:13 pm: This is a new patient – patient 3. The patient is an adult female. She has been brought to the ER in the company of the City County personnel and a Social Worker from the hospital. She is from a street family. She is in obvious distress; generally, she looks unwell and seems to be having respiratory problems. She has been put in a chair since all beds are currently full. We await further steps.

12:31 pm: The patient is still in obvious respiratory distress. She has not been reviewed yet. The patient is alone right now (not in the company of anyone – she is from the street families). She's sitting in a chair but still inobvious distress. I can see she's carrying some medications in her hands, seems to have been seen elsewhere prior to being brought here. She also has an IV cannula already fixed in her arm, apparently fixed outside the hospital. We hope she gets seen soon.

12:41 pm: The patient has just been seen, the vitals have been taken; BP seems to be 110/52 mmHg, HRis 109 b/min and SPO2 is 78% on room air. She's still in a chair, yet to be reviewed further. The beds arestill full, so the patient will have to wait in this chair a little longer.

1:07 pm: The patient has been put on oxygen via face mask. She's still seated on the chair. To note is thatthis patient seems to be having altered mental status with incoherent speech. She's still by herself but occasionally the EMTs are checking up on her, and she's yet to be evaluated in the next round of vital signs. Right now she doesn't seem good: still in severe respiratory distress and altered mental status.

1:15 pm: Patient is still on oxygen, status quo. The nurse has noticed that the patient keeps pulling off the oxygen mask from her face, doesn't want the mask put on her face. The patient still hasn't been reviewed by the doctor. No samples or further vitals have been taken. She's still on oxygen.

1:37 pm: The oxygen has been discontinued for now since the patient is uncooperative. The patient is still in a chair, no improvement. We still await a review, hopefully she'll get seen soon.

1:43 pm: The patient has been noted to have fallen off the chair and is lying on the floor, unresponsive. I have alerted the nurse and she has come accompanied with an EMT. The nurse and the EMT are surrounding the patient, trying to assist. The patient is unresponsive. We hope she's okay.

1:47 pm: The patient has just been confirmed dead. Just to repeat, the patient was brought in by a socialworker and the Nairobi City County team. She has no relatives as of this moment. The morgue is being informed shortly; the body is still on the floor in the ER.

3:00 pm: The body is being prepared for transport to the morgue. This marks the end of my follow-upwith the patient.

4f) Asample patient journey from the perspective of healthcare workers at Hospital 2 of a hypothetical patient requiring X-Ray, CT scan and ICU admission

Step	Activity	Minimum time (minutes)	Maximum time (minutes)
1	Patient arrives in the casualty department		
2	Patient is received by the triage nurse	2	30
3≠	Preliminary vital signs are taken by the nurse	1	2
4	Patient waits in the queue at the registration window	1	30
5≠	Confirmation of patient health cover/ payment of registration fee	2	5
6	Patient registration	5	10
7	Waiting in the queue for vital signs checking	1	30
8≠	Vital signs repeated and recorded by nurse	2	5
9	History taking by nurse	5	20
10	Waiting in the queue for review by clinician	5	45
11	Examination by clinician	5	15
12	History taking by clinician	5	15
13	Investigations (X-Ray and CT Scan) ordered	2	2
14	Payment of X-Ray fee at the cashier	5	45
15	Waiting in queue for X-Ray	15	120
16≠	Transport outside facility to seek the CT Scan	15	60
17	Waiting in queue for CT Scan	30	120
18≠	Waiting for interpretation of results	15	60
19	Travel back for review with CT Scan results	15	60
20	Waiting in queue for review with X-Ray and /CT Scan results	10	60
21	Doctor's review with results	10	45
22≠	Doctor calls a referral facility for ICU bed space	1	10
23	Waiting for availability of bed space (ICU)*	30	120
24≠	Ambulance set up and patient transported to destination facility	45	120

= waiting, ≠ = potential bottleneck

Registration is usually facilitated for a critically ill patient not accompanied by a relative

*Based on availability of ICU beds, the patient may wait for up to two hours or longer. In the event that no ICU space is available in the public health facilities the patient will have to source for space in private ICUs or wait until one avails in the public hospitals.

Consultation protocol

- Clinical officer (+/- clinical officer interns) sees patient
- Clinical officer calls Medical officer/registrar
- MO/registrar calls consultant

Investigations

- Available tests: RBS, FHG, UECs, LFTs, Ultrasound, X-Ray

- Not available: CT-Scan, MRI

Referral protocol (similar to Facility 1 in the main text)

- Through respective specialties;
- Consultant reviews patient, decides referral
- Unit manager notified
- MO/ Registrar calls destination hospital with patient details
- Ambulance set up and patient referred
