# Supplementary material for: Third delay in care of critically ill patients: a qualitative investigation of public hospitals in Kenya
Source: BMJ Open. 2024 Jan 4;14(1):e072341. doi: 10.1136/bmjopen-2023-072341 (PMC10773318; doi:10.1136/bmjopen-2023-072341)
Supplement: Author's manuscript [file bmjopen-2023-072341.draft_revisions.pdf]

BMJ Open

BMJ Open is committed to open peer review. As part of this commitment we make the peer review history of every article we publish publicly available.

When an article is published we post the peer reviewers' comments and the authors' responses online. We also post the versions of the paper that were used during peer review. These are the versions that the peer review comments apply to.

The versions of the paper that follow are the versions that were submitted during the peer review process. They are not the versions of record or the final published versions. They should not be cited or distributed as the published version of this manuscript.

BMJ Open is an open access journal and the full, final, typeset and author-corrected version of record of the manuscript is available on our site with no access controls, subscription charges or pay-per-view fees (http://bmjopen.bmj.com).

If you have any questions on BMJ Open's open peer review process please email info.bmjopen@bmj.com

BMJ Open

Third delay in care of critically ill patients: patient journeys in public hospitals in Kenya during the COVID-19 Pandemic

Journal:	*BMJ Open*
Manuscript ID	bmjopen-2023-072341
Article Type:	Original research
Date Submitted by the Author:	07-Feb-2023
Complete List of Authors:	Onyango, Onesmus; KEMRI-Wellcome Trust Research Programme, Health Services Unit<br>Willows, Tamara; University of Oxford, Nuffield Department of Medicine<br>McKnight, Jacob ; University of Oxford, Tropical Medicine; Oxford University<br>Schell, Carl Otto; Karolinska Institutet, Department of Global Public Health; Uppsala University, Department of Global Public Health<br>Baker, Tim; Karolinska Institutet; Ifakara Health Institute<br>Mkumbo, Elibariki; Ifakara Health Institute<br>Maiba, John; Ifakara Health Institute<br>Khalid, Karima; Ifakara Health Institute; Muhimbili University of Health and Allied Sciences<br>English, Mike; University of Oxford, Nuffield Department of Medicine ; KEMRI-Wellcome Trust Research Programme Nairobi,<br>Oliwa, Jacqueline; KEMRI-Wellcome Trust Research Programme Nairobi, Health Services Unit; University of Nairobi, Department of Paediatrics
Keywords:	ACCIDENT & EMERGENCY MEDICINE, COVID-19, Decision Making, Health Services Accessibility, Quality in health care < HEALTH SERVICES ADMINISTRATION & MANAGEMENT, QUALITATIVE RESEARCH

SCHOLARONE™
Manuscripts

BMJ

I, the Submitting Author has the right to grant and does grant on behalf of all authors of the Work (as defined in the below author licence), an exclusive licence and/or a non-exclusive licence for contributions from authors who are: i) UK Crown employees; ii) where BMJ has agreed a CC-BY licence shall apply, and/or iii) in accordance with the terms applicable for US Federal Government officers or employees acting as part of their official duties; on a worldwide, perpetual, irrevocable, royalty-free basis to BMJ Publishing Group Ltd ("BMJ") its licensees and where the relevant Journal is co-owned by BMJ to the co-owners of the Journal, to publish the Work in this journal and any other BMJ products and to exploit all rights, as set out in our licence.

The Submitting Author accepts and understands that any supply made under these terms is made by BMJ to the Submitting Author unless you are acting as an employee on behalf of your employer or a postgraduate student of an affiliated institution which is paying any applicable article publishing charge ("APC") for Open Access articles. Where the Submitting Author wishes to make the Work available on an Open Access basis (and intends to pay the relevant APC), the terms of reuse of such Open Access shall be governed by a Creative Commons licence – details of these licences and which Creative Commons licence will apply to this Work are set out in our licence referred to above.

Other than as permitted in any relevant BMJ Author's Self Archiving Policies, I confirm this Work has not been accepted for publication elsewhere, is not being considered for publication elsewhere and does not duplicate material already published. I confirm all authors consent to publication of this Work and authorise the granting of this licence.

For peer review only

Third delay in care of critically ill patients: patient journeys in public hospitals in Kenya during the COVID-19 Pandemic

Onesmus Onyango*[1], Tamara Willows[2], Jacob McKnight[2], Carl Otto Schell[4,5,6], Tim Baker[4,7,8,9], Elibariki Mkumbo[7], John Maiba[7], Karima Khalid[7,8], Mike English[1,2], Jacquie Oliwa[1,3]

1. Health Services Unit, KEMRI-Wellcome Trust Research Programme, Nairobi, Kenya
2. Health Systems Collaborative, Nuffield Department of Medicine, University of Oxford, UK
3. Department of Paediatrics, University of Nairobi, Nairobi, Kenya
4. Department of Global Public Health, Karolinska Institutet, Stockholm, Sweden
5. Centre for Clinical Research Sörmland, Uppsala University, Eskilstuna, Sweden
6. Department of Medicine, Nyköping Hospital, Nyköping, Sweden
7. Ifakara Health Institute, Dar es Salaam, Tanzania
8. Muhimbili University of Health and Allied Sciences, Department of Anaesthesia and Critical care, Tanzania
9. Department of Clinical Research, London School of Hygiene and Tropical Medicine, UK

*Correspondence
Onesmus Odongo Onyango
Health Services unit – KEMRI-Wellcome Trust Research Program, Nairobi, Kenya
OOnyango@kemri-wellcome.org

For peer review only

ABSTRACT

Objectives

Third delay refers to delays in delivering requisite care to patients after they arrive at a health facility. In low-resource care settings, effective triage and flow of care are difficult to guarantee. In this study, we aimed to identify delays in the delivery of care to critically ill patients in Kenyan secondary-level public hospitals and possible ways to address these delays.

Methods

This study was conducted in four secondary-level public Kenyan hospitals between March and December 2021 and employed a mix of in-depth interviews with frontline and administrative healthcare workers, observations during patient journey walkthroughs, field notes as well as informal conversations. The qualitative data were transcribed and aggregated into themes in NVivo 12 Plus using inductive coding.

Results

Our study identified a cycle of suboptimal systems for care with adaptive mechanisms that prevent quality care to critically ill patients. We identified suboptimal systems for identification of critical illness, inadequate resources for continuity care, and disruption of the flow of care, as the major causes of delays in *identification* and *the initiation of care* to critically ill patients. Our study also shed light on the contribution of non-clinical business- and protocol-related organizational processes in third delay.

Conclusion

Eliminating or reducing delays after patients arrive at the hospital is a time-sensitive measure that could improve the care outcomes of critically ill patients. This is achievable through an essential emergency and critical care package within the hospitals. Findings from our study can help emphasize the need for standardised effective and reliable care priorities to maintain of care of critically ill patients.

Study limitation and strengths

Our study was conducted in only four hospitals during the COVID-19 pandemic and was limited to only seven actual patient walkthrough journeys. A wider sample of facilities and patients would likely provide more robust evidence to explore our aim. However, this study draws strength from the multiple methods employed, which with triangulation, yielded valuable evidence and support. In addition, the study was conducted in facilities operating at the same level from four counties across different regions of the country, thus provided comparability. Lastly, the agreement of the staff from the hospitals with our findings as being a true reflection of their various facilities provided a validation for our study findings.

Keywords

Critical illness, Clinical decision, Essential care, Third delay, Critical Care, Low resource setting, LMICs, Patient safety, Quality of Care.

Wordcount – 5549 words

For peer review only

BACKGROUND

Critical illness is defined as a "*state of ill health with vital organ dysfunction, a high risk of imminent death if care is not provided and the potential for reversibility*" (1). Critically ill patients therefore require prompt, effective care wherever they are in health facilities to reduce the risk of dying. Critical care is herein described as identifying, monitoring and treating patients with critical illness through the initial and sustained support of vital organ functions (1). Although the true burden is unknown, the morbidity and mortality from critical illness are thought to be very high and constitute a considerable contribution to the global burden of disease (2-6). Many of the deaths caused by critical illness are potentially preventable (7, 8) if a prompt, basic, essential package of care (Appendix 1) is provided to these patients (9).

The *Three Delays* model (Figure 1) has been used for decades in obstetrics to illuminate obstacles to the provision of timely and quality care. The three delays are: 1) delay in making the decision to seek medical care (First Delay), 2) delay in arriving at a health facility after the decision to seek care has been made (Second Delay), and 3) delays in delivering care to patients after arrival to a health facility (Third Delay) (10, 11). While reducing all delays improves the clinical outcome of patients both in and outside the hospital, reducing the third delay directly reduces in-hospital morbidity and mortality. To address the third delay in the obstetric department in hospitals in resource-limited settings, a package of interventions was provided that focussed on adequate infrastructure, sufficient medical supplies, sufficiently trained health care providers at facilities, evidence-based care, and referral capacity to support transfers to higher-level care (12).

Early identification of critical illness is essential to its subsequent management and patient survival in health facilities that attend to large volumes of patients (13-16). Triage is the basis of care delivery to patients in the hospital emergency departments (17, 18) and consequently the first step in addressing third delay. During triage a critically ill patient should ideally be examined immediately on arrival, leading to a decision on the priority level of the patient's condition and their management. To engage in the triage process, facilities must have basic treatment materials, skilled personnel with full awareness of the resources and, if necessary, means of transportation for treatment or referrals to be carried out (19-21).

In low resource settings, effective triage and flow of care are difficult to accomplish. Healthcare facilities are frequently characterised by overcrowding, insufficient drugs, limited equipment and staff shortages (22) contributing directly to delay of care. The available evidence suggests that triage, basic emergency care training, better flow in hospitals, and supervision of junior emergency providers reduces mortality in low-resource settings (23). The most cited factor associated with lack of appropriate care in hospitals is shortage of human resources which affects many processes including triage (13). This shortage is compounded by inadequate training of available staff, lack of access to continuing education for healthcare workers, lack of motivation due to poor working conditions and low pay, as well as suboptimal supervision and management (12). To use limited human and material resources effectively , different forms of triage are used to classify patients according to the severity of their condition and determine the priority for further treatment (24, 25).

Critical illness gained significant global attention during the COVID-19 pandemic but strategies to improve the management of critically ill patients in resource-constrained settings are still in their infancy. While the third delay model helps us understand and address care gaps in maternal health within sub-Saharan Africa, the methods and findings of this approach have not been applied substantively to the delivery of care to critically ill patients outside the obstetric domain. Simple strategies such as mapping workflows could help identify points of intervention. In this paper we

aimed to identify delays in the delivery of care to critically ill patients in Kenyan secondary-level public hospitals. This literature offers potential insights for the use of a three-delays model in critical care.

For peer review only

METHODS

This study was part of a larger study – Provision of Essential Treatment in Critical Illness in the COVID-19 pandemic (POETIC) –a multi-method project investigating Essential Emergency and Critical Care (EECC) in Kenyan hospitals during the pandemic.

Study setting

Kenya has a population of approximately 48 million people served by 907 hospitals across 47 counties operating at primary, secondary and tertiary levels (26) (Figure 2). We included four purposively selected hospitals for our study that are part of the Clinical Information Network (CIN) because of their accessibility and level of care provided (27). All four hospitals included in our study received funding to scale up critical illness care provision during the COVID-19 pandemic. These hospitals had bed capacities ranging from 185 to 449.

Data collection

Process mapping and Patient journeys

We conducted process mapping of seven critically ill patients seeking various services at these facilities. The lead author OO identified frontline healthcare workers working in the emergency departments and with their help, mapped the journey of a critically ill patient when they arrived in the hospital. OO drew the process maps as described by the health workers in illustrative flow diagrams (Appendix 2 & Appendix 3). The healthcare workers were asked to estimate the time taken at each step along the journey (Table 1).

Table 1: A sample patient journey from the perspective of healthcare workers at Hospital 1 (this is a hypothetical patient who ends up needing CT scan)

Step	Activity	Minimum Time (minutes)	Maximum time (minutes)
1	Patient arrives in the casualty department		
2	Patient is received by the triage nurse	1	10
3	Preliminary vital signs are taken by the nurse	1	2
4	Patient waits in the queue at the registration window	1	30
5	Confirmation of patient health cover/ payment of registration fee	2	5
6	Patient registration	5	10
7	Waiting in the queue for vital signs checking	1	30
8	Vital signs repeated and recorded by nurse	2	5
9	History taking by nurse	5	20
10	Waiting in the queue for review by clinician	5	45
11	History taking by clinician	5	15
12	Examination by clinician	5	15
13	Investigations (CT scan) ordered	2	2
14	Transport outside facility to seek the required investigation	15	60
15	Waiting in queue for investigation	30	120
16	Waiting for interpretation of results	15	60
17	Travel back for review with CT Scan results	15	60
18	Waiting in queue for review with results	15	45
19	Doctor's review with results	10	45
20	Admission by nurse: nurse calls receiving ward for bed space	5	30

21	Waiting for availability of bed space	5	15
22	Nurse transports patient to destination ward	5	25

Consultation protocol

- Clinical officer (+/- clinical officer interns) sees patient
- Clinical officer calls Medical Officer/registrar
- MO/registrar calls consultant

Investigations

- Available tests: RBS, FHG, UECs, LFTs, Ultrasound, X-Ray, MRI (upon booking)
- Not available: CT-Scan, OGD

Referral protocol

- Through respective specialties;
  - Consultant reviews patient, decides referral
  - Unit manager notified
  - MO/ Registrar calls destination hospital with patient details
  - Ambulance set up and patient referred

The observer, OO, purposively selected and followed seven critically ill patients as determined by their signs and symptoms from the time of arrival at the Outpatient/Emergency department to the point of admission into the wards or referral out to another facility. The observer noted the care provided and periods of inactivity against the time. Using a Dictaphone, the observer made an audio commentary of the steps and activities against the time performed and this data was used to write out each patient journey (28, 29).

To explore third delay issues, we assessed the time taken in identification and administration of appropriate care for the selected patients. We used time to triage, time to diagnosis and time to management as proxies for determining third delay. *Time to triage* here referred to the time from patient's first contact with a healthcare worker (clinical officer, nurse, medical officer, or intern) to the time of departure to the next appropriate care point. Between this time and the time of an objective diagnosis was termed *time to diagnosis*. The time from when an objective diagnosis was made to the administration of appropriate care or intervention was defined as *time to initiation of care*.

In-depth Interviews (IDIs)

We used a semi-structured interview guide to conduct in-depth interviews (IDIs) with frontline healthcare workers – nurses, clinical officers, medical officers, and administrative staff. These interviews were conducted virtually via Microsoft Teams and telephone owing to the COVID-19 restrictions regarding face-to-face meetings. The IDI findings were used to build on the observations during the actual patient journeys. For this study, we used transcripts from twelve IDIs.

Patient and public involvement statement

The study design was advised by contemporary experiences of patients in Kenyan public hospitals, especially in light of the COVID-19 pandemic. Throughout the recruitment and conduct of the study, informed consent from patients and/or their relatives was maintained.

We held three feedback meetings with the hospitals after completion of data collection where we shared our findings and checked if our interpretation of the data made sense to hospital staff. Two of these (Facility 1 and Facility 2) were held in-person at the respective facilities, while one (Facility 3) was held virtually via Microsoft Teams. The fourth was unable to take part due to their COVID-19 workload. The audiences consisted of healthcare staff from various departments including accident & emergency, outpatient, surgery, paediatrics, medical ward, laboratory, radiology, nutrition, and hospital administration. The teams confirmed that our findings were a fair representation of their respective hospitals.

For peer review only

Data analysis

The audio recordings and observation notes were transferred to a password-protected cloud drive managed by the Kenya Medical Research Institute (KEMRI). The recordings and notes were transcribed verbatim in English and imported into NVivo® 12 Plus to generate nodes and to enable thematic analysis using inductive coding.

For peer review only

RESULTS

The following are findings from the patient journeys and themes derived from the in-depth interviews with the healthcare workers. These findings are summarised in the theoretical model we developed in this study (Figure 3).

Patient Journeys

These are transcripts of three out of the seven patient journeys we conducted across the four hospitals. The other patient journeys are attached in the appendices (Appendix 4).

Textbox 1

Context: *A County Referral Hospital. The A&E unit has five nurses on the shift – although one of them is the unit manager or 'in-charge' and, as such has duties other than frontline care. The unit also has two Clinical Officers and one Medical Officer. The Emergency Room (ER) has five couches, four oxygen ports, and there are two bedside monitors that are operational.*

An adult female patient arrives at **1020h**, accompanied by two relatives, one of whom is her sister. The nurse takes a brief history and identifies symptoms of severe abdominal pains. Four minutes after the patients' arrival, the nurse takes vital signs and the patient's SPO_2 is noted to be 93% on room air, and she has a slightly elevated heart rate of 90 beats per minute.

Twenty minutes later, the nurse takes her blood pressure measurement, and it is noted to be **93/50** mmHg. The patient is also found to be clinically pale. The nurse sets up an intravenous infusion of normal saline. At this point, the **clinician has ordered a laboratory request** for a full hemogram, but the sample is not delivered to the laboratory until the patient's relatives **make payments for the laboratory investigations – at 1204h, nearly two hours after she arrived**.

Over two hours after the initial vital signs, the Medical Officer reviews the patient and orders an abdominopelvic ultrasound. The **relative joins the queue at the payment window to make payments** for the investigation. The patient, in a wheelchair, then joins the queue at the radiology department.

At 1345h, a staff member at the radiology department **sends the patient's relative back to the A&E to communicate that the test cannot be done at the moment** because the patient is supposed to have fasted. The back-and-forth between these two departments continues until **1415h** when the doctor at the A&E orders specifically for a pelvic ultrasound. This is now four hours after the patient arrived.

Afterward, the patient is wheeled back to the radiology department and the **relatives queue up at the payment window to update payments for the new test**. The patient remains in the queue until 1733h when she is wheeled into the ultrasonography room. A diagnosis of **ruptured ectopic pregnancy** was made, and the patient was admitted **12 hours later**, at 2230h, for an 'urgent' surgical intervention: an **exploratory laparotomy**.

Textbox 1: Case 1 - A female patient presenting with progressive abdominal pains at Facility 1 (later diagnosed with ruptured ectopic pregnancy)

Context: *The Emergency Room (ER) and the general outpatient are housed under one roof. The unit has one nurse and one Medical Officer and is full of patients waiting to be seen. The nurse reported at 7:30am and will oversee this unit until 12:30pm when another nurse will come. The medical officer is also covering theatre in case of any surgical emergencies. The ER has three [3] beds/couches for patients, and at the moment, all three beds are occupied. There are also a few nursing students assisting in the care of these patients. Also, two Clinical Officers; one here in the emergency room, the other one covering the outpatient section. The unit has one functional port for piped oxygen which is currently in use. The ER is full. There's a COVID-19 isolation ward but currently that is full as well.*

An elderly male patient – a referral on follow-up for COVID-19 pneumonia – arrives at Facility 4 at **0857h** with symptoms of severe breathing difficulty, although the SPO_2 levels were above 95% on arrival. The patient is kept in the Emergency Department, awaiting allocation of space in the COVID-19 isolation ward at the Facility.

At **1217h**, the Medical Officer reviews the patient and noted the patient to be desaturating; SPO_2 as low as **84%. There's only one oxygen port at the ER, and at the moment it's in use by another patient**. The MO calls another facility, Facility X (a regional referral facility), since the isolation unit at Facility 4 is full. Facility X responds with a promise to call back once a space avails.

At **1252h** the SPO_2 levels are noted to be below **80%** and the patient is put on oxygen via face mask. The space at Facility X has not been confirmed yet. There's a back-and-forth between the medical officer and Facility X until **1543h** when the space in the isolation unit at Facility X is confirmed, and the ambulance is set up to ferry the patient.

Textbox 2 *2 - Journey of an adult male patient brought in with symptoms of severe difficulty in breathing at Facility 4*

Context: *The unit has ED has 8 beds, all occupied at the time the patient is brought in. this department has 3 nurses, 1 Medical Officer, and 2 Emergency Medical Technicians (EMTs). The unit is full of patients: some are seated on chairs, others waiting outside on benches. The department lacks piped oxygen but has three oxygen cylinders with flowmeters attached (no flow splitters). The three cylinders are all in use at the time the patient is brought in.*

Four minutes after arrival, at 1210h, the nurse takes vital observations, and the SPO2 is **85%** on room air. At 1222h, the patient is placed on a bed, **but he's sharing the bed with another patient** since the space is full. The patient awaits review. No intervention since the initial vital observations at 1210h. Ten minutes later, the Medical Officer instructs EMTs to fix an IV line and draw blood samples for laboratory investigations.

At 1235h, the Medical Officer writes a laboratory request and hands the blood samples to the patient's **relatives to deliver to the laboratory upon making payment for the tests.** Twenty minutes later, a nurse sets up a 500ml bottle of [illegible]ctate. The patient is yet to be reviewed.

Textbox 3

At 1325h, the **patient has been noticed to be having convulsions**. We still haven't had a concrete review because the doctor is quite held up and the patient's details have not been updated. Fifteen minutes later, the Medical Officer prescribes an anticonvulsant. The **prescription is handed to a relative to pick up the drug** from the pharmacy **upon payment**. The unit is still very busy, with very sick patients being wheeled in. The **medication arrives one hour later**, at 1441h, and is administered six minutes later. All this while the patient has not yet been reviewed and is still in bed in status quo; still unresponsive and having convulsions. The results from the lab are not yet out.

At **1615h**, the results are yet to be received back from the lab. No further vitals have been taken. The patient's condition hasn't improved much, but the convulsions have subsided for the moment. The patient is yet to be reviewed.

Textbox 3: Case 3 - The journey of an adult male patient received at the Emergency Department at Facility 3.

For peer review only - http://bmjopen.bmj.com/site/about/guidelines.xhtml

Themes

The main findings identified were ineffective identification of critical illness, inadequate resources for care, staffing shortages, interruptions from administrative processes, and lack of maintenance of EECC during patient flow. We combined these into three themes: suboptimal systems for identification of critical illness, inadequate resources for continuity care, and disruption of the flow of care.

Suboptimal systems for identification of critical illness

Informal triage systems (time to triage)

All four hospitals had a designated area for triage of patients as they arrive at the Casualty (Accident & Emergency) and Outpatient departments. Overall, all the facilities had processes to appraise a patient's vital signs – temperature, blood pressure, pulse rate, and oxygen saturation levels – and general status **within twenty minutes** of arrival (Table 1, Case 1, Case 3) at the Emergency departments, although they lacked routines and systems for identification and continuous patient monitoring after the initial evaluation.

> "Okay for that one [triage] it's not really clear. It is not clear; there is a no systematic triaging." – **Clinical Officer, Facility 1**.

At Facility 1, for example, upon entry into the facility, the patients were directed into the tent where they waited to be seen – mostly on a first-come-first-served basis. The tent was located away from the general outpatient and emergency departments and was set up at the advent of COVID-19 to screen patients. The tent was reported to be congested with no specific guidelines governing the categorization of these patients in the tent.

> "[…] there is delayed care because […] you know you have to remain in the triage until you're investigated for COVID at times there are no swabs, so you'll remain there. You see, the lab is far so even when they get their samples they have to go to the lab, back again, take care of other patients along the way you find that the patients can stay outside the tent for even twelve hours so there is delayed care." **Clinical Officer, Facility 1**.

Triage was therefore largely informal, and the flow of patients was dependent on the availability and number of the staff and resources, as facilities adopted routines that worked most conveniently for these understaffed departments. The stretched capacities in space and staff-to-patient ratios further compounded the effects of ineffective triage.

> "Ok in the triage system, sometimes you find that when we the staffs, there is shortage of staff. Like say you are alone for example like in a casualty Paeds [paediatric department]; our acute room is a bit far from where we do triage so when you're handling a patient in the acute room and there is nobody at the triage so you might miss out identification of a critically ill patient in the queue so such scenario, we might

For peer review only

end up losing a patient *akiwa kwa* (while still on the) queue." – **Nurse In-charge, Paediatric Department, Facility 2**.

Only Facility 3 had a functional formal triage system, adopting the South African Triage System (SATS) in the Emergency Department, using colour-coding – red, orange, yellow and green – to classify patients upon arrival.

"Initially we didn't have a proper triage system, but we brought it up that we needed a proper triage system and for the past I think three or four days we've been having a proper triaging system, we have like the triage sheet where you see and then we have the list of patients who are emergency patients who patients can wait and like that." **Medical Officer, A&E department – Facility 3**

"So, we are using the South Africa triage system whereby we have at least started coding the patients. We are using the red, orange, green and yellow. So, when a patient comes, we are using the triage chart, which is at the triage area…so we follow that triage, we fill everything and any patient who appears to be red there is…we tag the patient then we take him or her to the emergency area." **Nurse, Facility 3.**

Delays in early recognition of condition (time to diagnosis)

In-between the time the tests were ordered and when the results came back, the management of the patient was mainly symptomatic, with no system for continuous evaluation of patients' conditions (Case 1, Case 2, Case 3). Although the hypothetical journey map (Table 1) placed time to diagnosis within 35 minutes, findings from our actual patient journeys found that for tests available in the hospitals, it took 3-12 hours to make a clinical diagnosis after triage and initial evaluation.

Processing the laboratory and radiological tests ordered took considerable amounts of time. The delays were occasioned by long, inflexible registration and payment systems and long queues in these departments. The diagnostic departments were also noted to be limited in space and variety of tests, occasioning profound delays and often necessitating referrals.

"We only have X-ray, and ultrasound. Yeah, so we don't have a CT scan yet…and MRI we don't have. If we want to do an MRI or a CT scan we have to send outside." – **Nurse In-charge, Paediatric outpatient department – Facility 2**.

"Then the other thing is the laboratory. Our laboratory lacks many things; like there is no culture and sensitivity, those are things that miss in our lab. So, if we can have a bigger lab with many areas of assessment, it can be very much okay." – **Nursing Services Manager, Facility 1**.

Inadequate resources for continuity of essential care

Resources and infrastructure contribution to delay

Although the vital observations were done within 20 minutes of patients' arrival at the emergency departments, subsequent monitoring was delayed or not done altogether. The reasons cited for this included limited physical infrastructure and shortages and/or failure of equipment. This was picked across the four facilities as illustrated in the context information of the patient journeys and the following IDI quotes.

> "Like we have only one monitor and we encounter many patients, and you see you cannot put this one [patient] on a monitor *umnyanganye uweke mwengine* (then you take it from them to connect to another patient). We have one monitor. The BP machines are also limited they are like two. The equipment is there but we cannot say it's enough." – **Nurse, Facility 1.**

The few equipment available were sometimes broken down and often shared between the many patients in these departments, and occasionally between departments. This further impacted negatively on patient monitoring and care during interdepartmental/interfacility transfers.

> "...but sometimes you find the SPO_2 machine is not working, sometimes we don't have thermometers so we might miss out the degree of fever, so things like that. Sometimes they break down or they're stolen. Like the glucometer – you might travel with the patient but now if you can't check on the blood sugar level this patient might be in hypo, but you'll fail to note." – **Nurse, Facility 3**.

Lack of essential commodities and consumables in the destination departments resulted in slow movement of patients from the outpatient and emergency departments, further slowing down the identification and management of incoming critically ill patients.

> "I've seen only one point in Medical Ward. Oxygen is a challenge in the wards; most of the wards. And that is why you find patients of COVID stay here [Emergency Department] longer because they [wards] don't have piped oxygen like we, we have. They have to carry a cylinder. And right now, there is no oxygen." – **Nurse, Facility 3**.

As this was in the heart of the COVID-19 pandemic, in some cases, adaptive measures like referral and out-of-pocket purchase of these commodities were used to override the shortages and delays occasioned by unavailability of space and oxygen.

> "Like recently, the last three four days we didn't have oxygen. So, you tell the patient to make an option where he or she can go to, she can be treated, either Facility 88 or Facility 99 [private hospitals], because we cannot handle them. In real sense, this is a

For peer review only

government institution, whereby we are supposed to handle those COVID patients when they come." – **Nurse, Facility 3**.

Shortage of a skilled health workforce

Table 2 outlines the staffing structure across the departments in the four hospitals. Low staffing numbers we picked across departments in the four facilities.

Table 2: staffing structure across the facilities during the visit (numbers represent one shift).
Key: N= Nurses, CO= Clinical officers, MO= Medical officers.*

* The Medical Officers across all facilities covered more than one department during their shift
** Multiple department
s reduced their patient intake due to refurbishment work in Facility. Previous bed capacity for the medical and surgical departments was 32 beds each.

Department	Staff cadre	Staff-to-patient ratios (day shift)			
		Facility 1	Facility 2**	Facility 3	Facility 4
Accident & Emergency	N	4	5	3	2
	CO	1	4	0	2
	MO*	1	2	1	1
Obstetrics	N	10:12	8:37	9:73	2:19
	CO	2:12	2:37	0	0
	MO*	2:12	5:37	2:73	1:19
Paediatric ward	N	4:48	4:25	3:34	1:22
	CO	5:48	4:25	2:34	1:22
	MO*	2:48	3:25	1:34	2:22
Medical wards (male + female)	N	8:45	4:6	8:35	2:15
	CO	4:45	4:6	0	0
	MO*	2:45	4:6	2:35	1:15
Surgical wards (male + female)	N	8:50	3:8	8:51	3:12
	CO	2:50	3:8	0:51	0:12
	MO*	1:50	4:8	1:51	1:12
ICU	N	3:1		9:5	
	CO	1:1		1:5	
	MO	1:1	-	0	-

Across the hospitals, human resource shortage was a major hinderance to implementation of care interventions, and the available few staff could not allocate adequate focus to the critically ill patients in these departments.

"The other thing I would require staff who are very well trained. We would require protocols and all staff need to be trained about these protocols. That if a patient presents with this symptom, what next? You know, people don't just walk around without you know, knowing what to do." – **Medical Officer, Facility 4**.

The facilities lack an elaborate training system for staff on emergency and critical care, and since most of the staff train on the job, it is difficult to keep track of staff readiness to provide care to the critically ill patients.

"And also, many of us, you see we have not been trained all of us about the BLS and ACLS, those ones. Not all of them have been trained; so, if we can get any update training and motivation, that is, it can be very good. Because once you have knowledge, the skills and the technique, those are the three key things you'll need for production." – **Nursing Services Manager, Facility 1**.

Sometimes the strained staffing capacities led to coping measures such as rationing of care even seriously ill patients, in order to spread out the care to as many patients as possible.

"In the wards there is also shortage, so they just check in general, and they if they deteriorate there is resuscitation and they make it, sometimes they don't make it" **Nurse, Facility 1**

Disruption of the flow of care (time to initiation of care)

The role of administrative processes in delay – registration and payments

Across the four hospitals, payment for registration, laboratory investigations, and radiological tests were a barrier to quality of care and smooth flow of patients between care points. Staff in some facilities felt that it would help if there were systems to hold off payments for basic tests until later when the results are out, and the patient has been attended to.

"The issue that we have is this issue of funds. A patient may come to you presenting with hypoglycaemia and you don't know…it's an altered mental status. You really don't know, because the…the relatives have to pay for the RBS [random blood sugar]. You understand? I would require that we have a very clear path with the lab so that we don't require patients to pay out for things like RBS, you know. Essential tests like BS for P. falciparum. So that in that clear path patients can always pay for these things later once, once they get well or whether they make it, or they don't make it" – **Medical Officer, A&E department – Facility 4**

For peer review only

Besides lacking systems to hold off payments for registration or investigations and tests for critically ill patients in the four facilities, long queues at the payment point contributed towards delays in care for the critically ill patients across the four hospitals. Some facilities reported having a waiver process if a patient is unable to pay for a test or investigation. This is done through the office of the hospital Social Worker when they are available. However, the process is slow, and care is greatly delayed before the waiver is effected.

> "So, the challenge is *pesa* (money)…money, and you see the patient cannot be waived, nowadays, you have to pay cash. And when you go to the social worker, what he'll tell you is that he or she can waive to a certain point – not beyond maybe I don't know, 2000 [Kenyan shillings], not beyond 3000. That is now the challenge." – **Nurse In-charge A&E department – Facility 3**

Inefficient communication systems

Lack of direct interdepartmental communication lines was picked across all four hospitals. Most departments lacked a functional departmental telephone, and communication between departments was largely through the health workers' personal mobile phones.

> "In fact, I was forgetting. That is a very big problem. We don't have communication equipment. It used to be there but *ziliharibika zote* (they all broke down) so right now we use our phones. Sometimes you find we call personally; you find somebody has decided to be *mteja* (offline). So, communication is very important. We are lacking communication. To get an MO [medical officer] or even to get someone to call, to connect; it's very difficult because you have to call personal phones. Somebody can even ignore. Maybe if that one can be put in place will be able to help our patients" **Nurse, Facility 2.**

In some cases, the faulty communication hardware meant that the staff had to walk to other departments to relay the information in person.

> "At least when you are taking there a patient, they, you should tell them at least prior, tell them earlier to prepare, we are bringing such and such a patient. It has been a challenge anyway and it's still a challenge. It's not [clear]…because the phones are here but they're still not functioning. And it's…it's also difficult because of shortage every time I keep on walking in the ward, *"Naleta mgonjwa"* (I'm bringing a patient), I'm bringing a patient, you know, it's also cumbersome." – **Nurse In-charge A&E department – Facility 3**

For peer review only

In the four facilities, communication generally relied on the office of the Covering Nurse even in emergencies. This office serves as a call centre for the hospital to provide liaison between departments and to link the hospital with other facilities for inbound and outgoing referrals. In every shift, there's a designated nurse who takes up the role, with a designated facility phone. The covering nurse receives and relays information between the relevant personnel or departments, including the administration when needed. With absence of EECC and lack of processes to maintain care in-between, the protocol for escalation of care was also described as long and time-consuming, especially during night shifts and weekends.

> "The nurse now has to look for the nurse covering and tell the nurse covering that we have a patient, and this patient is to be reviewed by a medical officer. Then the nurse covering calls the medical officer, at the same time the nurse covering calls the driver that is night-time. So that is when the driver will go, bring the, the medical officer to see the patient. At times it takes one hour, two hours, three hours. And sometimes the medical officer is within the hospital yes but doing an operation. So, it means the patients have to wait until the operation is over, that is when this medical officer will come to review the patient. That is what happens in the night. In…over the weekend, again there is that delay." **– A&E Nurse, Facility 4**

When probed about the possibility of a better and more prompt communication system, some of the facilities reported that it would be a welcome upgrade, although budgetary constraints would cripple the implementation and sustainability of such a system.

In general, *time to initiation of care* was between 4-12 hours. In Case 1, a follow-up call the following day revealed that the patient was admitted past 10:30 pm and line for an emergency surgical intervention, although the patient had arrived at the facility more than 12 hours prior.

For peer review only

DISCUSSION

We qualitatively examined the patient monitoring and evaluation processes and the limiting factors in the journey of critically ill patients when seeking care and yielded findings congruent with global literature (12, 13, 21, 30-33). Figure 3 summarises our concept for the causes of third delay in care of critically ill patients.

We identified ineffective communication systems, and administrative challenges as the major limitations to reducing the *time to initiation of care* – one of our indicators for third delay - of critically ill patients. Although the hospitals and units have 'protocols' in place for receiving critically patients to other destination departments, the practicality of these inter-disciplinary linkages was an evident challenge. Arranging and executing the inter-departmental transfers from one care point to the next e.g., from the Emergency department to radiology/laboratories and back prior to admission into wards took between 3-12 hours. During these times, care was often withheld as the logistics were sorted out. In addition, the existence of a functional reliable communication system is key in carrying through the urgency of care for critically ill patients across the departments. In Case 1 at Facility 1, for example, the one-and-a-half-hour delay due to dysfunctional communication between the A&E and the radiology department could have been avoided if a reliable communication channel between the two departments existed. Patient-specific information, particularly in the ED, is an essential component of the patient pathway, patient-physician relationship and nursing practice and team working (34). There needs to be a system of communication in the radiology and laboratory departments, too, to ensure maintain the continuity of triage and urgency of care for critically ill patients.

We found that while the hospitals had allocated physical space – room or cubicle – in the emergency and outpatient departments for triage, time to triage was markedly increased and effective triage practice was mostly postponed or forfeited altogether. Shortage of material resources described by others contributed greatly to missed triage and monitoring of patients (12, 21, 22). Secondly, the acute shortage of human resources noted across the facilities due to low employment rates and high staff turnovers affected both initial and subsequent evaluation and review of critically ill patients, as the healthcare workers focused on clearing the overwhelming queues in these departments. This has forced adaptive mechanisms like on-job staff training in the ICU. These findings are congruent with those by Morof et. al. and Knight et. al. that identified human resource factor, in volume and capacity, as the most common barrier to prompt identification and subsequent management of critical illness (12, 13).

Our study also shed light on the contribution of administrative processes – non-clinical business- and protocol-related organizational processes – in third delay. We identified lack prompt systems to override payments for tests, investigations, and essential services for critically ill patients, all of which contribute towards delays in receipt of adequate and appropriate treatment. This occurred because prescribed tests and investigations were not processed until the patients, or their relatives paid the requisite fees attached to these services. Even in facilities that have waiver systems for patients who cannot afford a service, the processes are often very slow and unreliable in the event of critical illness. While it is vital to account for all the services offered to patients in the facilities, there should be straightforward systems in place to ensure that the payments or lack thereof, do not interfere with the care delivered to critically ill patients and that care is not withheld when the patient is not able to raise the amounts required, or when the payments are yet to be processed. Reducing the turn-around-time in emergencies is key to favourable prognosis, therefore eliminating these administrative stumbling blocks will help reduce the congestion in the emergency care pathways and well as capitalize on the cost-effectiveness of the basic emergency care interventions (35-37).

For peer review only - http://bmjopen.bmj.com/site/about/guidelines.xhtml

In chaos, the risks are profound. COVID-19 was pivotal to illuminating areas for improvement in the ill-prepared hospital systems globally. In summary, the findings from our study speak to a series of dysfunctional systems for care with adaptive mechanisms that are unideal for the care of critically ill patients. Implementing a guiding principle that the most time-critical, life-saving and feasible care is the priority when resources are scare, might be a helpful staring point in improving quality of care and outcomes hospitals. Such a policy, as articulated in the recent national strategic plan for implementing EECC in Tanzania (38) could be useful for the change needed to address defective triage, improper initial and continued evaluation of critically ill patients, strained resource capacities, and administrative bottlenecks all impede the flow of care for critically ill patients while seeking care in these secondary-level public hospitals as seen in the lengthy delays in triage, diagnosis, and definitive management. All these culminate into a marked third delay in patients seeking care in these hospitals, and in the case of critically ill patients, the delays could be detrimental to their clinical outcome and lead to preventable mortality.

CONCLUSION

Our study revealed how common long-standing ineffective intra and inter-facility patient flow pathways that are unreliable in critical illness, are across many facilities. While this approach has allowed facilities to remain functional, critical illness require prompt systems to transmit the need for urgency across multiple care points.

Eliminating third delay is an essential and time-critical measure that would improve the care outcomes of critically ill patients in facilities in resource-constrained settings. This is achievable with a package of essential emergency and critical care within the hospitals. Findings from our study can help emphasize the need for standardised effective and reliable care priorities to maintain of care of critically ill patients within hospitals.

For peer review only

ACKNOWLEDGEMENTS

We are grateful to Prof. Claudia Hanson, Senior Lecturer, Karolinska Institutet, Sweden, Professor, London School of Hygiene & Tropical Medicine, UK and Dr Roy Nobhojit, Surgeon and Researcher, BARC Hospital, HBNI University, Mumbai, India, for their invaluable mentorship that helped shape the concept and direction of this study.

We wish to thank the POETIC-COVID team and the entire EECC network for the immense support and teamwork throughout the study period and beyond. We would also like to acknowledge the contribution of the participating hospitals and their staff in the process of this study.

Contributorship statement

OO conceptualized and designed the study, acquired and analysed the data, and developed the first draft of the manuscript with assistance from TW, JO, and JMcK. TB, ME, and COS contributed to the conceptualization and design of the study. TW, EM, JM, KK, and OO analysed the data together. JO also contributed to acquisition of data and was the Scientific Lead for the larger POETIC-COVID study. All the authors interpreted the findings, critically revised the manuscript, and approved the final version.

Conflict of interest statement

The authors in this study all declare no conflicts of interest.

Funding Statement

This study was funded through the POETIC-COVID project by a grant from Wellcome Trust [221571/Z/20/Z] as part of the *Innovation in Low and Middle-income Countries* Flagship. ME is supported by a Wellcome Senior Fellowship (#207522) with further support for authors from a core grant awarded to the KEMRI-Wellcome Trust\Research Programme (#092654).

Data sharing statement

Additional anonymised transcripts of patient journeys, process maps, and in-depth interviews are attached separately as supplemental material submitted alongside this manuscript.

Ethics approval statement

This study was part of a larger study approved by the KEMRI Scientific and Ethical Review Committee (SERU Number 4085) and London School of Hygiene and Tropical Medicine (REF 22 866). Institutional approvals were also obtained at hospital level and/or their relevant County liaison for all four hospitals.

Individual consent was sought from the participants (healthcare workers and patients/relatives) regarding participation and recording of the interviews and observations. It was explained that anonymity would be ensured regarding the use of the transcribed interviews, observations, and voice notes in the patient journeys. We explained to the participants that all the audio recordings pertaining to the walkthroughs would be deleted once the recordings were transcribed.

For peer review only

REFERENCES

1. Kayambankadzanja RK, Schell CO, Warnberg MG, Tamras T, Mollazadegan H, Holmberg M, et al. Towards definitions of critical illness and critical care using concept analysis. medRxiv. 2022. https://dx.doi.org/10.1136/bmjopen-2022-060972
2. Adhikari NK, Fowler RA, Bhagwanjee S, Rubenfeld GD. Critical care and the global burden of critical illness in adults. The Lancet. 2010;376(9749):1339-46. https://doi.org/10.1016/S0140-6736(10)60446-1
3. Bell MB, Konrad D, Granath F, Ekbom A, Martling C-R. Prevalence and sensitivity of MET-criteria in a Scandinavian University Hospital. Resuscitation. 2006;70(1):66-73. https://doi.org/10.1016/j.resuscitation.2005.11.011
4. Dart PJ, Kinnear J, Bould MD, Mwansa S, Rakhda Z, Snell D. An evaluation of inpatient morbidity and critical care provision in Zambia. Anaesthesia. 2017;72(2):172-80. https://doi.org/10.1111/anae.13709
5. Kruisselbrink R, Kwizera A, Crowther M, Fox-Robichaud A, O'Shea T, Nakibuuka J, et al. Modified early warning score (MEWS) identifies critical illness among ward patients in a resource restricted setting in Kampala, Uganda: a prospective observational study. PloS one. 2016;11(3):e0151408. https://doi.org/10.1371/journal.pone.0151408
6. Razzak J, Usmani MF, Bhutta ZA. Global, regional and national burden of emergency medical diseases using specific emergency disease indicators: analysis of the 2015 global burden of disease study. BMJ global health. 2019;4(2):e000733. https://dx.doi.org/10.1136/bmjgh-2018-000733
7. Molyneux E, Ahmad S, Robertson A. Improved triage and emergency care for children reduces inpatient mortality in a resource-constrained setting. Bulletin of the World Health Organization. 2006;84:314-9.
8. Roy N, Kizhakke Veetil D, Khajanchi MU, Kumar V, Solomon H, Kamble J, et al. Learning from 2523 trauma deaths in India-opportunities to prevent in-hospital deaths. BMC health services research. 2017;17(1):1-8. https://doi.org/10.1186/s12913-017-2085-7
9. Schell CO, Khalid K, Wharton-Smith A, Oliwa J, Sawe HR, Roy N, et al. Essential Emergency and Critical Care: a consensus among global clinical experts. BMJ Glob Health. 2021;6(9). https://dx.doi.org/10.1136/bmjgh-2021-006585
10. Barnes-Josiah D, Myntti C, Augustin A. The “three delays” as a framework for examining maternal mortality in Haiti. Social science & medicine. 1998;46(8):981-93. https://doi.org/10.1016/S0277-9536(97)10018-1
11. Thaddeus S, Maine D. Too far to walk: maternal mortality in context. Social science & medicine. 1994;38(8):1091-110.
12. Morof D, Serbanescu F, Goodwin MM, Hamer DH, Asiimwe AR, Hamomba L, et al. Addressing the third delay in Saving Mothers, Giving Life districts in Uganda and Zambia: ensuring adequate and appropriate facility-based maternal and perinatal health care. Global Health: Science and Practice. 2019;7(Supplement 1):S85-S103. https://doi.org/10.9745/GHSP-D-18-00272
13. Knight HE, Self A, Kennedy SH. Why are women dying when they reach hospital on time? A systematic review of the 'third delay'. PLoS One. 2013;8(5):e63846. https://doi.org/10.1371/journal.pone.0063846
14. Sorensen BL, Rasch V, Massawe S, Nyakina J, Elsass P, Nielsen BB. Advanced life support in obstetrics (ALSO) and post-partum hemorrhage: a prospective intervention study in Tanzania. Acta Obstet Gynecol Scand. 2011;90(6):609-14. https://doi.org/10.1111/j.1600-0412.2011.01115.x

For peer review only

15. Vora N, Maitra N, Pandya P. Use of the Maternal Foetal Triage Index-Addressing the Third Delay in Obstetrics: An Observational Study. Authorea Preprints. 2021. https://doi.org/10.22541/au.162142765.52779042/v1
16. Gupta S, Khajanchi M, Kumar V, Raykar NP, Alkire BC, Roy N, et al. Third delay in traumatic brain injury: time to management as a predictor of mortality. Journal of neurosurgery. 2019;132(1):289-95. https://doi.org/10.3171/2018.8.JNS182182
17. Bullard MJ, Unger B, Spence J, Grafstein E, Group CNW. Revisions to the Canadian emergency department triage and acuity scale (CTAS) adult guidelines. Canadian Journal of Emergency Medicine. 2008;10(2):136-42. https://doi.org/10.1017/S1481803500009854
18. Göransson KE, Ehrenberg A, Ehnfors M. Triage in emergency departments: national survey. Journal of clinical nursing. 2005;14(9):1067-74. https://doi.org/10.1111/j.1365-2702.2005.01191.x
19. Cavallaro FL, Marchant TJ. Responsiveness of emergency obstetric care systems in low-and middle-income countries: A critical review of the “third delay”. Acta obstetricia et gynecologica Scandinavica. 2013;92(5):496-507. https://doi.org/10.1111/aogs.12071
20. Gabrysch S, Campbell OM. Still too far to walk: literature review of the determinants of delivery service use. BMC pregnancy and childbirth. 2009;9(1):1-18. https://doi.org/10.1186/1471-2393-9-34
21. Downar J, Smith MJ, Godkin D, Frolic A, Bean S, Bensimon C, et al. A framework for critical care triage during a major surge in critical illness. Can J Anaesth. 2022;69(6):774-81. https://doi.org/10.1007/s12630-022-02231-2
22. Floyd L, Bryce F, Ramaswamy R, Olufolabi A, Srofenyoh E, Goodman D, et al. The introduction of a midwife-led obstetric triage system into a regional referral hospital in Ghana. Midwifery. 2018;61:45-52. https://doi.org/10.1016/j.midw.2018.02.003
23. Hsia RY, Thind A, Zakariah A, Hicks ER, Mock C. Prehospital and emergency care: updates from the disease control priorities, version 3. World J Surg. 2015;39(9):2161-7. https://doi.org/10.1007/s00268-015-2997-5
24. Sasser S, Kellermann A, Lormand J. Prehospital trauma care systems. Geneva: World Health Organization; 2005. Arh Hig Rada Toksikol. 2013;64:567-79.
25. Surgeons CoTotACo. Resources for optimal care of the injured patient 2006. Chicago, IL: American College of Surgeons. 2006.
26. MOH. Kenya Harmonized Health Facility Assessment (KHFA) 2018/2019. Nairobi MOH; 2019.
27. Tuti T, Bitok M, Malla L, Paton C, Muinga N, Gathara D, et al. Improving documentation of clinical care within a clinical information network: an essential initial step in efforts to understand and improve care in Kenyan hospitals. BMJ Global Health. 2016;1(1):e000028. http://dx.doi.org/10.1136/bmjgh-2016-000028
28. Holden RJ, Carayon P. SEIPS 101 and seven simple SEIPS tools. BMJ Quality & Safety. 2021;30(11):901-10. http://dx.doi.org/10.1136/bmjqs-2020-012538
29. Simonse L, Albayrak A, Starre S. Patient journey method for integrated service design. Design for Health. 2019;3(1):82-97. https://doi.org/10.1080/24735132.2019.1582741
30. De Freitas L, Goodacre S, O’Hara R, Thokala P, Hariharan S. Qualitative exploration of patient flow in a Caribbean emergency department. BMJ open. 2020;10(12):e041422. http://dx.doi.org/10.1136/bmjopen-2020-041422
31. Tang C, Chen Y, Lee S. Non-clinical work counts: facilitating patient outflow in an emergency department. Behaviour & Information Technology. 2015;34(6):585-97. https://doi.org/10.1080/0144929X.2014.963673
32. Van Vaerenbergh C. Using mixed methods to identify factors influencing patient flow. Health Serv Manage Res. 2009;22(4):170-5. https://doi.org/10.1258/hsmr.2009.009006

For peer review only

33. Organization WH. The hospital in rural and urban districts: report of a WHO Study Group on the Functions of Hospitals at the First Referral Level [meeting held in Geneva, 30 October-5 November 1990]: World Health Organization; 1992.
34. Cohen EL, Wilkin HA, Tannebaum M, Plew MS, Haley LL. When Patients Are Impatient: The Communication Strategies Utilized by Emergency Department Employees to Manage Patients Frustrated by Wait Times. Health Communication. 2013;28(3):275-85. https://doi.org/10.1080/10410236.2012.680948
35. In: Jamison DT, Breman JG, Measham AR, Alleyne G, Claeson M, Evans DB, et al., editors. Disease Control Priorities in Developing Countries. Washington (DC)New York: The International Bank for Reconstruction and Development / The World Bank.Oxford University PressCopyright © 2006, The International Bank for Reconstruction and Development/The World Bank Group.; 2006.
36. Jayaraman S, Mabweijano JR, Lipnick MS, Caldwell N, Miyamoto J, Wangoda R, et al. First things first: effectiveness and scalability of a basic prehospital trauma care program for lay first-responders in Kampala, Uganda. PLoS One. 2009;4(9):e6955. https://doi.org/10.1371/journal.pone.0006955
37. Shanahan T, Risko N, Razzak J, Bhutta Z. Aligning emergency care with global health priorities. Int J Emer Med. 2018;11(1):1-4. https://doi.org/10.1186/s12245-018-0213-8
38. MOH T. National Strategic Plan on Essential Emergency and Critical Care Services (2023-2026). 2022. 2022.

For peer review only

Figure Legends

1. Figure 1: The framework of the Third Delay model (adapted from Gabrysch & Campbell, and Cavallaro & Marchant). The loop represented by the green connectors illustrates the continuous re-assessment and monitoring of acutely ill patients needed to identify critical illness. The loop represented by the orange dotted connectors represents a second referral to a higher-level facility. Any complications that cannot be managed at the first facility should be promptly diagnosed and referred within the shortest time possible to eliminate **second emergency delay**.

2. Figure 2: Organization of the Kenyan public health system

3. Figure 3: A theoretical concept of the causes of Third Delay in the care of patients presenting with clinical illness.

For peer review only

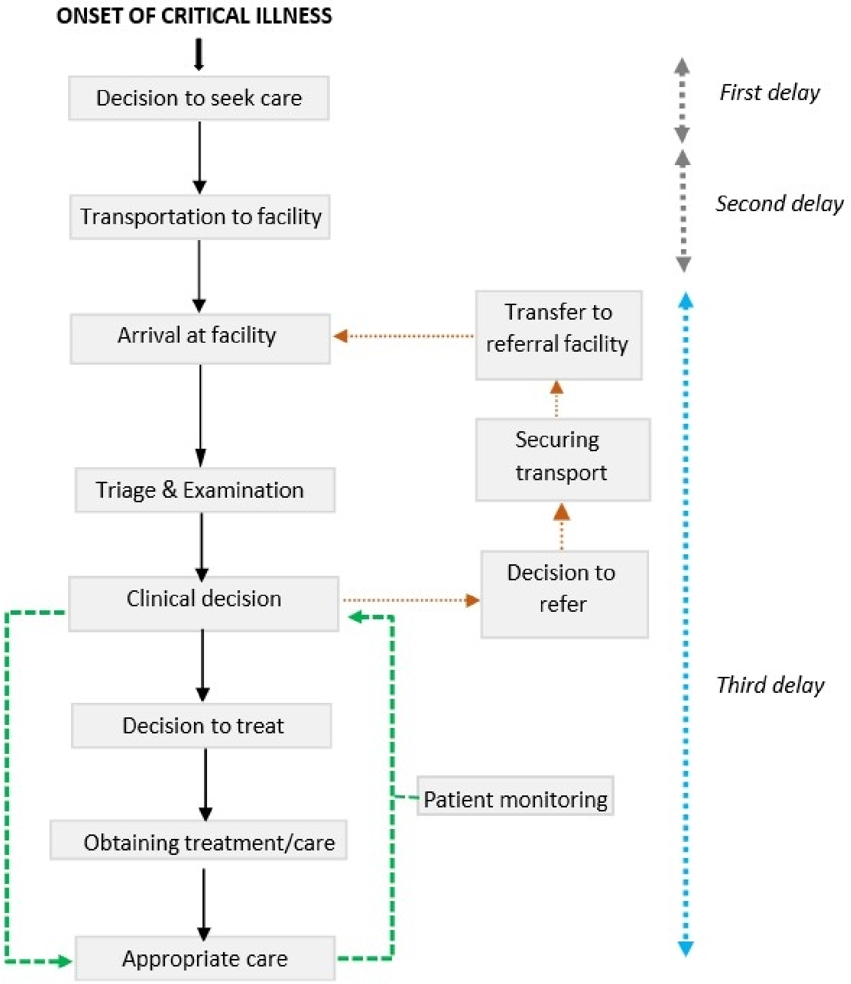

The framework of the Third Delay model (adapted from Gabrysch & Campbell, and Cavallaro & Marchant). The loop represented by the green connectors illustrates the continuous re-assessment and monitoring of acutely ill patients needed to identify critical illness. The loop represented by the orange dotted connectors illustrates a second referral to a higher-level facility. Any complications that cannot be managed at the first facility should be promptly diagnosed and referred within the shortest time possible to eliminate second emergency delay.

146x155mm (120 x 120 DPI)

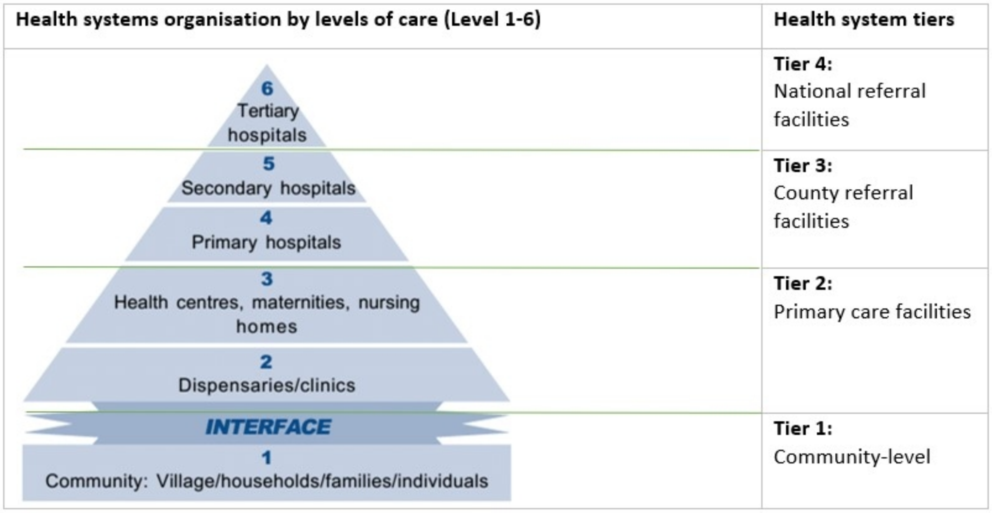

Organization of the Kenyan public health system by levels of care

170x87mm (120 x 120 DPI)

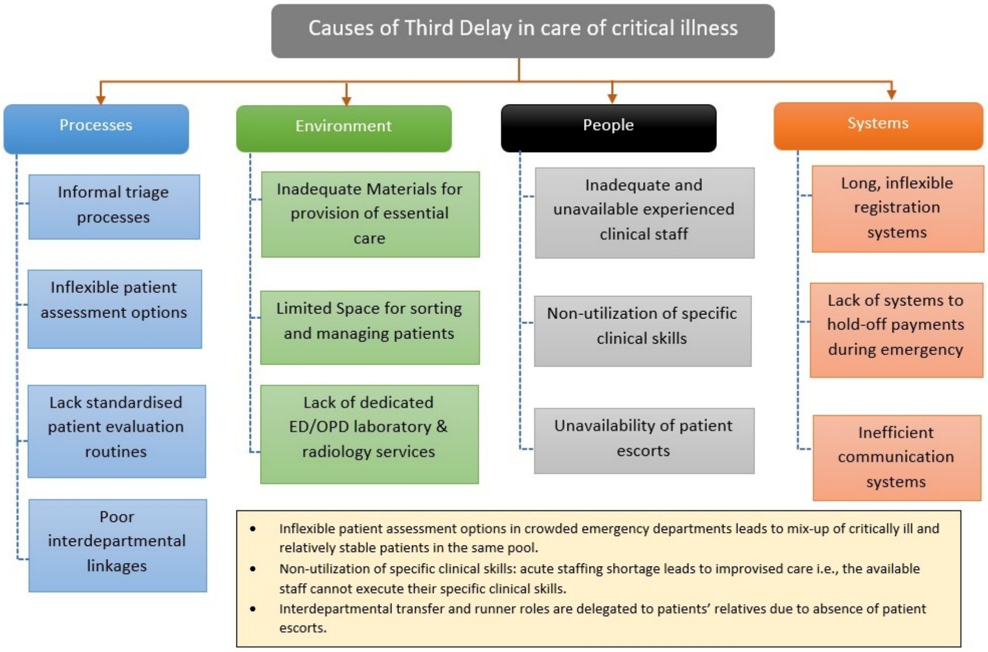

A theoretical concept of the causes of Third Delay in the care of patients presenting with clinical illness.

211x144mm (120 x 120 DPI)

APPENDICES

Appendix 1: EECC checklists

PANEL 2. The Hospital Readiness Requirements for Essential Emergency and Critical Care

IDENTIFICATION OF CRITICAL ILLNESS

The following items are required for a hospital to be ready for the identification of critically ill patients:

CATEGORY	ITEM
1.1. EQUIPMENT	1.1.1 Clock with second hand<br>1.1.2 Pulse oximeter & probe<br>1.1.3 Blood pressure measuring equipment (eg. sphygmomanometer with a stethoscope)<br>1.1.4 Blood pressure cuffs of different paediatric and adult sizes<br>1.1.5 Light source (lamp or flashlight)<br>1.1.6 Thermometer
1.2 CONSUMABLES	1.2.1 Soap or hand disinfectant<br>1.2.2 Examination gloves
1.3 DRUGS	None
1.4 HUMAN RESOURCES	1.4.1 Health workers with the ability to identify critical illness 24h/day
1.5 TRAINING	1.5.1 The health workers are trained in the identification of critical illness
1.6 ROUTINES	1.6.1 Routines for the identification of critical illness
1.7 GUIDELINES	1.7.1 Guidelines for the identification of critical illness
1.8 INFRASTRUCTURE	1.8.1 Designated triage area (area for the identification of critical illness) in the Out-Patient Department or Emergency Unit (area of the hospital where patients arrive)<br>1.8.2 Running water

only

PANEL 2 continued …

CARE OF CRITICAL ILLNESS

The following items are required for a hospital to be ready to provide the care of critically ill patients:

CATEGORY	ITEM
2.1 EQUIPMENT	2.1.1 Suction machine (electric or manual)<br>2.1.3 Oxygen supply 24h/day (cylinder, concentrator (with electricity supply) or piped oxygen)<br>2.1.4 Flow meter (if using cylinder or piped oxygen)<br>2.1.5 Leak-free connectors from oxygen source to tubing<br>2.1.6 Bag Valve Mask (resuscitator) – neonatal, paediatric and adult sizes<br>2.1.7 Sharps disposal container<br>2.1.8 External heat source
2.2 CONSUMABLES	2.2.1 Suction catheters of paediatric and adult sizes<br>2.2.2 Guedel airways of paediatric and adult sizes<br>2.2.3 Pillows<br>2.2.4 Oxygen tubing<br>2.2.5 Oxygen nasal prongs<br>2.2.6 Oxygen face masks of paediatric and adult sizes<br>2.2.7 Oxygen face masks with reservoir bags of paediatric and adult sizes<br>2.2.8 Masks for Bag Valve Mask (resuscitator) – neonatal, paediatric and adult sizes<br>2.2.9 Compression bandages<br>2.2.10 Plasters or tape<br>2.2.11 Gauze<br>2.2.12 Intravenous cannulas of paediatric and adult sizes<br>2.2.13 Intravenous giving sets<br>2.2.14 Skin disinfectant for cannulation<br>2.2.15 Syringes<br>2.2.16 Nutrition<br>2.2.17 Nasogastric tubes<br>2.2.18 Lubricant for nasogastric tube insertion<br>2.2.19 Intramuscular needles<br>2.2.20 Intraosseous cannulas of different sizes<br>2.2.21 Blankets<br>2.2.22 Facemasks for Infection Prevention and Control<br>2.2.23 Aprons or gowns<br>2.2.24 Charts/notes for documentation<br>2.2.25 Pens
2.3 DRUGS	2.3.1 Oral rehydration solution<br>2.3.2 Intravenous crystalloid fluids (eg. normal saline or Ringer's Lactate)<br>2.3.3 Intravenous dextrose fluid (eg. 5%, 10% or 50%)<br>2.3.4 Oxytocin<br>2.3.5 Adrenaline<br>2.3.6 Appropriate antibiotics<br>2.3.7 Diazepam<br>2.3.8 Magnesium sulphate<br>2.3.9 Paracetamol<br>2.3.10 Local anaesthetic (eg. 2% lignocaine) (eg. for intraosseous cannulation)
2.4 HUMAN RESOURCES	2.4.1 Health workers with the ability to care for critically ill patients 24hrs/day<br>2.4.2 Senior health worker who can be called to assist with the care of critically ill patients 24hrs/day

PANEL 2 continued …

2.5 TRAINING	2.5.1 The health workers are trained in the care of critically ill patients
2.6 ROUTINES	2.6.1 Routines for managing critically ill patients<br>2.6.2 Routine for the provision of EECC without taking into account patients' ability to pay<br>2.6.3 Routines for who and how to call to seek senior help 24hrs/day, 7 days/week<br>2.6.4 Routines for integrating EECC with other care including the definitive care of the underlying condition (eg. use of condition-specific guidelines)
2.7 GUIDELINES	2.7.1 Guidelines for the essential care of critically ill patients
2.8 INFRASTRUCTURE	2.8.1 Designated space for the care of critically ill patients (eg. a bay, ward, high dependency unit)<br>2.8.2 Areas for separating and managing patients with a suspected or confirmed contagious disease from those without

PANEL 3. The essential diagnosis-specific care for critically ill patients with COVID-19

CLINICAL PROCESSES

1. The Essential Emergency and Critical Care (EECC) clinical processes as specified for all critical illnesses
2. Personal Protective Equipment (PPE) that is appropriate for COVID-19 as part of Infection, Prevention and Control (IPC)
3. Monitoring oxygen saturation using pulse oximetry at least every 6 hours, unless otherwise prescribed
4. Intermittent prone positioning
5. Low molecular weight heparin or other anticoagulant
6. Corticosteroid
7. Antibiotics in patients with suspected bacterial superinfection

HOSPITAL READINESS REQUIREMENTS

Critically ill patients with COVID-19 require the same hospital readiness for EECC as other critically ill patients. For the provision of the essential diagnosis specific care of critically ill patients with COVID-19, the following additional items are required:

CATEGORY	ITEM
3.1 EQUIPMENT	None
3.2 CONSUMMABLES	3.2.1 Facemasks appropriate for COVID-19 (eg. N95)<br>3.2.2 Eye protection or face shields
3.3 DRUGS	3.3.1 Low-molecular weight heparin (eg. enoxaparin or dalteparin) or other anticoagulant<br>3.3.2 Corticosteroid (eg. dexamethasone)
3.4 HUMAN RSOURCE	3.4.1 Health workers with the ability to care for critically ill COVID-19 patients 24hrs/day
3.5 TRAINING	3.5.1 The health workers are trained in essential care of critically ill COVID-19 patients
3.6 ROUTINES	3.6.1 Routines for care of critically ill COVID-19 patients
3.7 GUIDELINES	3.7.1 Guidelines for essential care of critically ill COVID-19 patients
3.8 INFRASTRUCTURE	3.8.1 Areas for separating and managing patients with suspected or confirmed COVID-19 from those without

Appendix 2: Process map – Facility 1

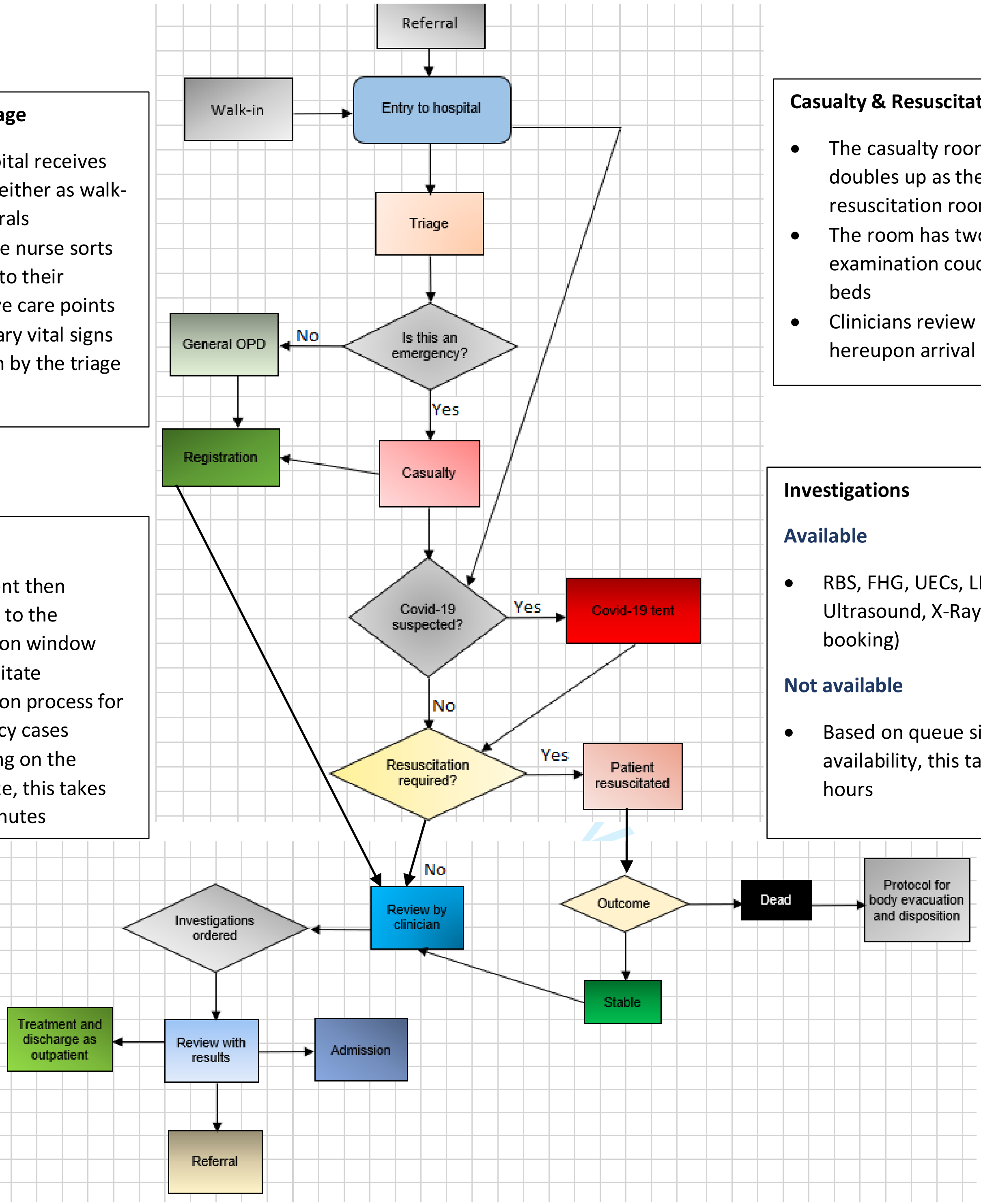

Entry and triage

- The hospital receives patients either as walk-ins/referrals
- The triage nurse sorts patients to their respective care points
- Preliminary vital signs are taken by the triage nurse

Casualty & Resuscitation

- The casualty room also doubles up as the resuscitation room
- The room has two examination couches and 5 beds
- Clinicians review patients hereupon arrival

Registration

- The patient then proceeds to the registration window
- Staff facilitate registration process for emergency cases
- Depending on the queue size, this takes 10-45 minutes

Investigations

Available

- RBS, FHG, UECs, LFTs, Ultrasound, X-Ray, MRI (upon booking)

Not available

- Based on queue size and availability, this takes 1-4 hours

Consultation protocol

- Clinical Officer (+/- clinical officer interns) sees patient
- Clinical Officer calls Medical Officer/registrar
- MO/registrar calls consultant

Appendix 3: Process map – Facility 3

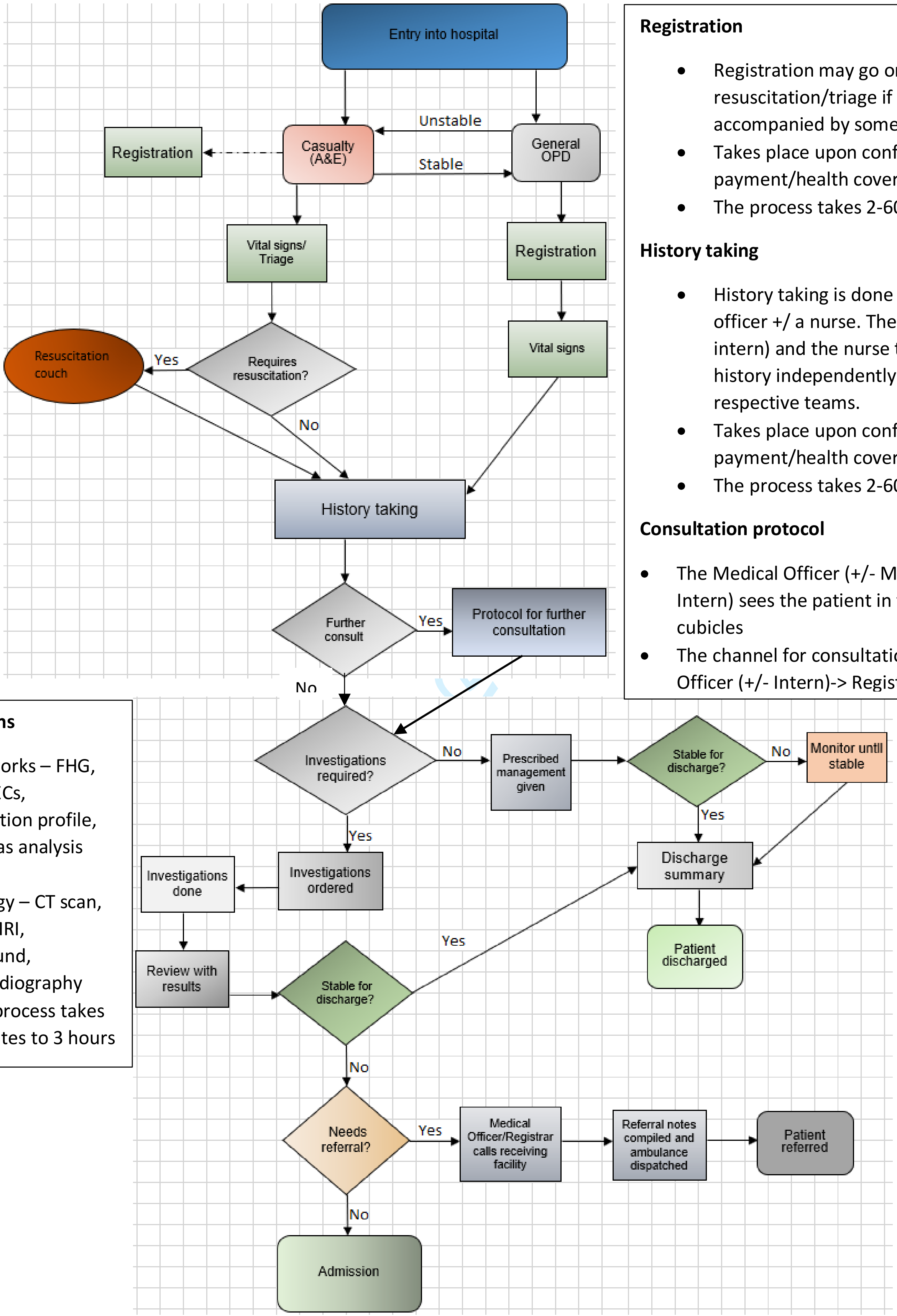

Registration

- Registration may go on during resuscitation/triage if the patient is accompanied by someone
- Takes place upon confirmation of payment/health cover
- The process takes 2-60 minutes

History taking

- History taking is done by a medical officer +/ a nurse. The MO (or MO + MO intern) and the nurse take patient history independently for their respective teams.
- Takes place upon confirmation of payment/health cover
- The process takes 2-60 minutes

Consultation protocol

- The Medical Officer (+/- Medical Officer Intern) sees the patient in the consultation cubicles
- The channel for consultation is Medical Officer (+/- Intern)-> Registrar -> Consultant

Investigations

- Blood works – FHG, LFTs, UECs, Coagulation profile, Blood gas analysis (BGA)
- Radiology – CT scan, X-ray, MRI, Ultrasound, Echocardiography
- Whole process takes 30 minutes to 3 hours

Appendix 4: Patient Journeys

Patient 2 Facility 4

Journey of a patient who presented with symptoms of severe difficulty in breathing (Covid-19 patient).

Date: 09/06/2021

Narrator: OO

0830h: Morning. This is OO, the ARO for the POETIC-COVID study. It is about 8:30 am, a patient has just been wheeled into the casualty [ER] with apparent symptoms of severe breathing difficulties. The patient is being wheeled onto a couch in the casualty. The Medical Officer is still not yet in. The doctor [Medical Officer] was here overnight, so he has stepped out to refresh before coming back. Upon coming back, we hope the patient will be reviewed and if at all there are any investigations to be taken the patient will be taken accordingly. That's all for now, we wait for the next steps.

0835h: Update for Patient 2, it is 8:35 am. The vitals have just been taken. It has been noted that the heart rate is a little unstable, ranging as low as from 50s to 60s but at times it's spiking, so not really stable. She's in obvious breathing difficulty [distress]. The SPO2 currently is reading 58% off oxygen. To be noted again is that the unit has one pulse oximeter that is operational. There is another one attached to the monitor but not working. The other patients…there is one other patient who needs constant pulse oximetry, but he has his own…the family came with a personal pulse oximeter, so I think for that he's sorted for now. That is all for now, I'll keep you posted.

0900h: Update for Patient 2, it's 9 am. The doctor arrived at around 8:57 am. He has reviewed the patient as well as the others. He's suggesting an X-ray, so the relatives have gone to the registration window to open a file for the patient. So, we await that after which the prescribed test [X-ray] …she'll be wheeled in.

0915h: Patient 2, the time is 9:15 am. The patient has just been wheeled in for the X-ray. Being that she is among the very first patients who reported to this casualty we are hoping that it won't take long, she's going to be attended to almost immediately. So, we hope that she'll be out in the next few minutes.

0937h: The patient has just left the X-ray department, awaiting review with results [results are not out yet] …so, the patient is back in the casualty. I'll keep you posted. It's now 9:37 am, the patient went in at 9:15 am and is already out. So, we'll follow up upon review of the results.

0950h: The patient has been put on oxygen, in bed at the casualty department awaiting the X-ray results. Not really connected to a monitor because the monitors are faulty, but the patient is on oxygen, so from time to time the vitals are being checked. I'll keep you posted.

1015h: The results are out…the X-ray results are out but apparently there has to be a Covid test done. The RDT [Covid rapid test] is not available in that department so the patient has to be taken away again; a stone-throw away within the hospital for the test. The relatives are still following up with whoever is responsible on how best that can be done, but otherwise, the patient is still on oxygen, we are awaiting a review of the X-ray results. There is a queue…there's one patient ahead so the Medical Officer is still with another patient but once he's done, I think the patient will be reviewed with the X-ray results. However, we are still following up with the Covid team for the Covid test. I'll keep you posted.

For peer review only

1043 h: Update for Patient 2, 10:43 am. The Covid team came to take samples for the rapid test from the casualty. The samples are being taken, so we await the next few minutes for the results to be out after which it'll inform the direction of care; whether this patient is going to be admitted in the medical ward or the Covid isolation ward. I'll keep you posted, that's the update for now.

1127h: Update for Patient 2, it is 11:27am. The test results for Covid-19 are out, she has been confirmed to be Covid positive. So, the patient has been indicated to be admitted in the Covid isolation ward. Initially they had planned to admit her in the female medical ward, but now that the results are out, she's going to be taken to the isolation unit. One thing about the isolation unit (I was already there a just a while ago), it has a capacity of six [6]. It has a total of 6 beds. At the moment four [4] of those are occupied, so we are hoping that this patient is going to get space. We are just hopeful that the space avails…we keep on waiting at the casualty. The patient is still on oxygen via face mask, the vitals-the SPO2 is okay. She's saturating on 97% on oxygen, but the pulse rate was a little worrisome initially. It was going as low as 52, at one point even at 43 beats per minute but at the moment we cannot say that she's so badly off. She's still on oxygen, pulse rising to above 68. That's the update for now.

1140h: Update for Patient 2. The vitals have been taken again…still the SPO2 is ranging between 90-93% on oxygen. To be noted again is that there is no high-flow oxygen in this facility due to the oxygen supply [strained]. Again, in the casualty there is on oxygen port which is being used to give oxygen to this patient via mask. There's no non-rebreather mask in the department as at now but hopefully the SPO2 will rise, it is promising. Again, this unit is actually very full at this point, remember there is only one medical officer. The unit is full and other patients are actually complaining. Also there's another patient who is having similar issues [as Patient 2] and they [the medical team] were trying to locate a bed space for the patient…there has been a push-and-pull between the Medical Officer here and the destination hospital [FACILITY X], they have been arguing over phone and the Medical Officer seems frustrated here. Looks like he feels his hands are tied and the patients need help and they cannot be able to help but we are hoping that things ease up over the next few minutes.

1254h: Update for Patient 2, the time is 12:54 pm. It has been confirmed that there is bed space for this patient in the Covid isolation ward in this Facility. Her latest SP02 was 94%. So, we are waiting for a stretcher to be brought so that the patient can be wheeled there.

1304h: Update for Patient 2, the time is 1304h. The patient has just been put on the stretcher to be taken to the Covid isolation ward…the patient is being admitted in the Covid isolation ward. The patient is to be taken there on portable oxygen in a cylinder. They're setting that up. I'll keep you posted once the patient lands in the Covid isolation ward. They have just left the casualty.

1319h: Update for Patient 2, the time is 1319h. The patient has just been transferred to the Covid isolation unit…patient has been admitted. To be noted is that this patient is currently saturating at 94% on oxygen but on admission [when she landed at the casualty] the patient was actually saturating as low as 17% - that was off oxygen. The patient has been maintained in the casualty on oxygen and has been transferred into the Covid isolation unit. As it stands the patient has been admitted into the isolation unit with Covid-19 Pneumonia. I'll halt there for now, that marks the end of my journey with Patient 2.

For peer review only

Facility: Facility 2

Date: September 13, 2021

Narrator: OO

11:40 am: I am in the A&E department at Facility 2. The ER has two rooms; A& B, each with 4 beds. At the moment all eight beds are occupied. There's no piped oxygen in the department but I can count three oxygen cylinders with a capacity of 50.2L each. Right now, two of the cylinders are in use. Each cylinder has a flowmeter attached, but no flow splitters. The cylinders serve one patient at a time.

11:45 am: An adult male patient has been brought in, carried by two relatives, apparently unconscious.

11:48 am: The patient is in a chair (all 8 beds are currently occupied), still not responsive. The nurse and an assistant - looks like a trained Patient Attendant or an EMT – are attending to the patient. They are setting up to take the patient's vital signs. The SPO2 probe has just been put. The EMT has fixed an IV line. The RBS is 3.4 mmol/L. A blood sample has been drawn, to be taken to the lab upon payment by therelatives. I'll keep you posted.

12:15 pm: The patient is still in a chair since the 4 beds in this cubicle are occupied. There is an adjacent cubicle but that as well is currently full. The nurse and an EMT are trying to set up an IV fluid, but they first have to settle the patient in a bed. They are trying to create a bed space by relocating another patient who has stabilized. Just to add, this department has 3 nurses, 1 Medical Officer and 2 EmergencyMedical Technicians (EMTs). The EMT assist with wheeling the patients as well as fixing IV lines.

12:21 pm: The patient has been put onto a bed, but he's sharing the bed with another patient since the space is full. The nurse has just put up a 500 ml bottle of 5% IV Dextrose. The patient's relatives have been given the sample to deliver to the lab upon making payment at the laboratory department.

12:40 pm: The IV Dextrose is still running. No further review at this point. The department is quite busy and packed with patients. The results are not yet out from the lab.

12:48 pm: The patient is currently responsive; he is waking up. The IV Dextrose is still running, but still no further review yet.

1:15 pm: The IV fluid (5% Dextrose) is over. The patient is awake and looks stable. We still await review.The lab results are not out yet. The patient has been taken outside to wait on the bench.

1:50 pm: The patient is stable and has been asked to wait outside on the bench in the waiting bay so as to create a bed space for another patient. The results are not ready from the lab. The patient will be reviewed once the results are out.

For peer review only

Patient 3:

Facility: Facility 2

Narrator: OO

12:13 pm: This is a new patient – patient 3. The patient is an adult female. She has been brought to the ER in the company of the City County personnel and a Social Worker from the hospital. She is from a street family. She is in obvious distress; generally, she looks unwell and seems to be having respiratory problems. She has been put in a chair since all beds are currently full. We await further steps.

12:31 pm: The patient is still in obvious respiratory distress. She has not been reviewed yet. The patient isalone right now (not in the company of anyone – she is from the streets). She's sitting in a chair but still inobvious distress. I can see she's carrying some medications in her hands, seems to have been seen elsewhere prior to being brought here. She also has an IV cannula already fixed in her arm, apparently fixed outside the hospital. We hope she gets seen soon.

12:41 pm: The patient has just been seen, the vitals have been taken; BP seems to be 110/52 mmHg, HRis 109 b/min and SPO2 is 78% on room air. She's still in a chair, yet to be reviewed further. The beds arestill full, so the patient will have to wait in this chair a little longer.

1:07 pm: The patient has been put on oxygen via face mask. She's still seated on the chair. To note is thatthis patient seems to be having altered mental status with incoherent speech. She's still by herself but occasionally the EMTs are checking up on her, and she's yet to be evaluated in the next round of vital signs. Right now she doesn't seem good: still in severe respiratory distress and altered mental status.

1:15 pm: Patient is still on oxygen, status quo. The nurse has noticed that the patient keeps pulling off theoxygen mask from her face, doesn't want the mask put on her face. The patient still hasn't been reviewedby the doctor. No samples or further vitals have been taken. She's still on oxygen.

1:37 pm: The oxygen has been discontinued for now since the patient is uncooperative. The patient is stillin a chair, no improvement. We still await a review, hopefully she'll get seen soon.

1:43 pm: The patient has been noted to have fallen off the chair and is lying on the floor, unresponsive. The nurse and the EMT are surrounding the patient, trying to assist. The patient is unresponsive. Wehope she's okay.

1:47 pm: The patient has just been confirmed dead. Just to repeat, the patient was brought in by a socialworker and the Nairobi City County team. She has no relatives as of this moment. The morgue is being informed shortly; the body is still on the floor in the ER.

3:00 pm: The body is being prepared for transport to the morgue. This marks the end of my follow-upwith the patient.

For peer review only

BMJ Open

Third delay in care of critically ill patients: a qualitative investigation of public hospitals in Kenya.

Journal:	*BMJ Open*
Manuscript ID	bmjopen-2023-072341.R1
Article Type:	Original research
Date Submitted by the Author:	29-Aug-2023
Complete List of Authors:	Onyango, Onesmus; KEMRI-Wellcome Trust Research Programme, Health Services Unit<br>Willows, Tamara; University of Oxford, Nuffield Department of Medicine<br>McKnight, Jacob ; University of Oxford, Tropical Medicine; Oxford University<br>Schell, Carl Otto; Karolinska Institutet, Department of Global Public Health; Uppsala University, Department of Global Public Health<br>Baker, Tim; Karolinska Institutet; Ifakara Health Institute<br>Mkumbo, Elibariki; Ifakara Health Institute<br>Maiba, John; Ifakara Health Institute<br>Khalid, Karima; Ifakara Health Institute; Muhimbili University of Health and Allied Sciences, Department of Anaesthesia and Critical care<br>English, Mike; University of Oxford, Nuffield Department of Medicine ; KEMRI-Wellcome Trust Research Programme Nairobi,<br>Oliwa, Jacqueline; KEMRI-Wellcome Trust Research Programme Nairobi, Health Services Unit; University of Nairobi, Department of Paediatrics
**Primary Subject Heading**:	Emergency medicine
Secondary Subject Heading:	Emergency medicine, Evidence based practice, Nursing, Patient-centred medicine, Public health
Keywords:	ACCIDENT & EMERGENCY MEDICINE, COVID-19, Decision Making, Health Services Accessibility, Quality in health care < HEALTH SERVICES ADMINISTRATION & MANAGEMENT, QUALITATIVE RESEARCH

SCHOLARONE™
Manuscripts

I, the Submitting Author has the right to grant and does grant on behalf of all authors of the Work (as defined in the below author licence), an exclusive licence and/or a non-exclusive licence for contributions from authors who are: i) UK Crown employees; ii) where BMJ has agreed a CC-BY licence shall apply, and/or iii) in accordance with the terms applicable for US Federal Government officers or employees acting as part of their official duties; on a worldwide, perpetual, irrevocable, royalty-free basis to BMJ Publishing Group Ltd ("BMJ") its licensees and where the relevant Journal is co-owned by BMJ to the co-owners of the Journal, to publish the Work in this journal and any other BMJ products and to exploit all rights, as set out in our licence.

The Submitting Author accepts and understands that any supply made under these terms is made by BMJ to the Submitting Author unless you are acting as an employee on behalf of your employer or a postgraduate student of an affiliated institution which is paying any applicable article publishing charge ("APC") for Open Access articles. Where the Submitting Author wishes to make the Work available on an Open Access basis (and intends to pay the relevant APC), the terms of reuse of such Open Access shall be governed by a Creative Commons licence – details of these licences and which Creative Commons licence will apply to this Work are set out in our licence referred to above.

Other than as permitted in any relevant BMJ Author's Self Archiving Policies, I confirm this Work has not been accepted for publication elsewhere, is not being considered for publication elsewhere and does not duplicate material already published. I confirm all authors consent to publication of this Work and authorise the granting of this licence.

For peer review only

Third Delay in care of critically ill patients: a qualitative investigation of public hospitals in Kenya.

Onesmus Onyango*[1], Tamara Willows[2], Jacob McKnight[2], Carl Otto Schell[4,5,6], Tim Baker[4,7,8,9], Elibariki Mkumbo[7], John Maiba[7], Karima Khalid[7,10], Mike English[1,2], Jacquie Oliwa[1,3]

1. Health Services Unit, KEMRI-Wellcome Trust Research Programme, Nairobi, Kenya
2. Health Systems Collaborative, Nuffield Department of Medicine, University of Oxford, UK
3. Department of Paediatrics, University of Nairobi, Nairobi, Kenya
4. Department of Global Public Health, Karolinska Institutet, Stockholm, Sweden
5. Centre for Clinical Research Sörmland, Uppsala University, Eskilstuna, Sweden
6. Department of Medicine, Nyköping Hospital, Nyköping, Sweden
7. Ifakara Health Institute, Dar es Salaam, Tanzania
8. Department of Emergency Medicine, Muhimbili University of Health and Allied Sciences, Tanzania
9. Department of Clinical Research, London School of Hygiene and Tropical Medicine, UK
10. Department of Anaesthesia and Intensive Care, Muhimbili University of Health and Allied Sciences, Tanzania

*Correspondence
Onesmus Odongo Onyango
Health Services unit – KEMRI-Wellcome Trust Research Program, Nairobi, Kenya
OOnyango@kemri-wellcome.org

For peer review only

For peer review only - http://bmjopen.bmj.com/site/about/guidelines.xhtml

ABSTRACT

Objectives

Third Delay refers to delays in delivering requisite care to patients after they arrive at a health facility. In low-resource care settings, effective triage and flow of care are difficult to guarantee. In this study, we aimed to identify delays in the delivery of care to critically ill patients in Kenyan secondary-level public hospitals and possible ways to address these delays.

Methods

This exploratory qualitative study was conducted in four secondary-level public Kenyan hospitals between March and December 2021. We used 12 in-depth interviews with 11 healthcare workers and patient journeys of 7 patients. The qualitative data were transcribed and aggregated into themes in NVivo 12 Plus using inductive coding. Nodes containing information about referrals were aggregated to build themes.

Results

Our study identified a cycle of suboptimal systems for care with adaptive mechanisms that prevent quality care to critically ill patients. We identified suboptimal systems for identification of critical illness, inadequate resources for continuity care, and disruption of the flow of care, as the major causes of delays in *identification* and *the initiation of care* to critically ill patients. Our study also illuminated the contribution of non-clinical business- and protocol-related organizational processes in Third Delay.

Conclusion

Eliminating or reducing delays after patients arrive at the hospital is a time-sensitive measure that could improve the care outcomes of critically ill patients. This is achievable through an essential emergency and critical care package within the hospitals. Findings from our study can help emphasize the need for standardised effective and reliable care priorities to maintain of care of critically ill patients.

Article summary

This paper highlights a qualitative investigation of delays in identification, monitoring, and treatment of critically ill patients in resource-constrained settings. Critical illness is hereby described as any potentially reversible condition presenting with vital organ dysfunction with a risk of imminent death if care is not provided promptly.

Our study shows the limitations that resource shortages impose on the nature and quality of care administered, and the rigid compensatory systems that emerge. The findings suggest a need for a holistic consideration of workflows with accountability of the whole hospital system, not just focusing on resources, to ensure this performance is maintained across the care pathways.

We conclude that as a standard, the administration of an essential emergency and critical care (EECC) package and sustained transmission of the care urgency is a time-critical and cost-effective measure to improve the clinical outcomes of critically ill patients in low-resource settings.

Strengths and limitations of this study

- Our study was conducted in only four hospitals during the COVID-19 pandemic and was limited to only 12 interviews and 7 actual patient walkthrough journeys.
- A wider sample of facilities, healthcare providers, and patients would likely provide more robust evidence to explore our aim.
- However, this study draws strength from the multiple methods employed, which with triangulation, yielded valuable evidence and support.
- In addition, the study was conducted in facilities operating at the same level from four counties across different regions of the country, thus provided comparability.

For peer review only - http://bmjopen.bmj.com/site/about/guidelines.xhtml

- Lastly, the agreement of the staff from the hospitals with our findings as being a true reflection of their various facilities provided a validation for our study findings.

For peer review only

For peer review only - http://bmjopen.bmj.com/site/about/guidelines.xhtml

BACKGROUND

Critical illness is defined as a "*state of ill health with vital organ dysfunction, a high risk of imminent death if care is not provided and the potential for reversibility*" (Kayambankadzanja et al., 2022). Critical care is herein described as identifying, monitoring and treating patients with critical illness through the initial and sustained support of vital organ functions. Critically ill patients therefore require prompt, effective care wherever they are in health facilities to reduce the risk of dying. Although the true burden is unknown, the morbidity and mortality from critical illness are thought to be very high and constitute a considerable contribution to the global burden of disease (Adhikari et al., 2010; Bell et al., 2006; Dart et al., 2017; Kruisselbrink et al., 2016; Razzak et al., 2019). Many of the deaths caused by critical illness are potentially preventable (Molyneux et al., 2006; Roy et al., 2017) if a prompt, basic, essential package of care (Appendix 1) is provided to these patients (Schell et al., 2021).

Critical illness gained significant global attention during the COVID-19 pandemic but strategies to improve the management of critically ill patients especially in resource-constrained settings are still in their infancy. With the overwhelming cases of critically ill patients during the COVID-19 pandemic, the Kenyan government – through the national and respective county governments – embarked on setting up COVID-19 isolation units and intensive care units (ICUs) as well as training healthcare workers to improve intensive care capacity (Maina et al., 2020). However, these efforts were limited by weak public hospital systems and marked health workforce deficits (Kenya, 2020; Zhao et al., 2020), and facilities continued to see an upsurge of critically ill patients.

Triage is the basis of early identification of critical illness and consequently the first step in the management and survival of critically ill patients in health facilities that attend to large volumes of patients (Gupta et al., 2019; Knight et al., 2013; Sorensen et al., 2011; Vora et al., 2021). A critically ill patient should ideally be examined immediately on arrival, leading to a decision on the priority level of the patient's condition and their management. For appropriate triage and care delivery, hospitals must have basic treatment materials, skilled personnel with full awareness of the resources and, if necessary, means of transportation for treatment or referrals to be carried out (Cavallaro & Marchant, 2013; Downar et al., 2022; Gabrysch & Campbell, 2009). Available evidence suggests that triage, basic emergency care training, better flow in hospitals, and supervision of junior emergency providers reduces care delays and mortality in low-resource settings (Hsia et al., 2015). In low resource settings, effective triage and flow of care are however difficult to accomplish. Healthcare facilities are frequently characterised by overcrowding, insufficient drugs, limited equipment, and staff shortages (Floyd et al., 2018; Knight et al., 2013; Morof et al., 2019) as well us a lack of understanding of critical illness (Mkumbo et al., 2023), contributing directly to delay in essential care. To use the limited human and material resources effectively, different forms of triage are used to classify patients according to the severity of their condition and determine the priority for further treatment (Sasser et al., 2013; Surgeons, 2006).

The *Three Delays* model(Barnes-Josiah et al., 1998; Thaddeus & Maine, 1994) (Figure 1) has been used for decades in obstetrics to illuminate obstacles to the provision of timely and quality care. The First Delay refers to delays in making the decision to seek medical care, mostly due to family and community reasons. Second Delay refers to delays in arriving at a health facility after the decision to seek care has been made due to accessibility challenges. The Third Delay is concerned with delay in delivering care to patients after arrival to a health facility. Reducing all delays improves the clinical outcome of patients both in and outside the hospital, but reducing the Third Delay directly reduces in-hospital morbidity and mortality by adopting a holistic approach to understand the responsibilities of hospital systems.

For peer review only - http://bmjopen.bmj.com/site/about/guidelines.xhtml

While the Third Delay model helps us understand and address care gaps in maternal health in resource-constrained settings, the methods and findings of this approach have not been applied substantively to the delivery of care to critically ill patients, especially in low-resource hospitals, outside the obstetric domain. Application of the Third Delay concept through simple strategies such as mapping workflows could therefore be a useful model to identify points of urgent intervention for all patients presenting with critical illness in these settings. Using the Third Delay concept, we aimed to identify delays in the delivery of care to critically ill patients in Kenyan secondary-level public hospitals.

For peer review only

For peer review only - http://bmjopen.bmj.com/site/about/guidelines.xhtml

METHODS

Study design

This was an exploratory qualitative study design to observe care provided to critically ill patients. The study was part of a larger multi-method project – Provision of Essential Treatment in Critical Illness in the COVID-19 pandemic (POETIC-COVID) – that sought to investigate Essential Emergency and Critical Care (EECC)(Schell et al., 2021) in Kenyan hospitals during the pandemic. We used in-depth interviews and patient journeys across the selected hospitals and analysed the results in detail using inductive and deductive approaches.

Study setting

Kenya, a lower middle-income country, has a population of approximately 48 million people served by 907 hospitals across 47 counties operating at primary, secondary and tertiary levels (MOH, 2019)(Figure 2). The study was conducted in four secondary-level public Kenyan hospitals (Figure 2) across four of the 47 counties in Kenya. The hospitals selected for our study were part of the Clinical Information Network (CIN) (Tuti et al., 2016) and were selected purposively(Palinkas et al., 2015) in four counties based on the level of care they provide and their accessibility from Nairobi, where the lead researcher was based, owing to COVID-19 restrictions. All four hospitals included in our study had received funding to scale up critical illness care provision during the COVID-19 pandemic. These hospitals had bed capacities ranging from 185 to 449.

Participant selection and recruitment

A purposive sampling method (Palinkas et al., 2015) was used to identify administrative staff and frontline healthcare providers from the emergency and out-patient departments, intensive care units (ICUs), maternity units and general wards (paediatric and adult wards). Using staff clinical judgement, the patients were picked from the emergency departments that typically received critically ill patients in these hospitals.

We explained to the eligible participants the purpose and procedure of the study, and that privacy and confidentiality, and their right to opt out at their convenience would be upheld. The number of participants to interview was guided by thematic saturation (Lowe et al., 2018; Pope et al., 2000), that is, when no new findings were emerging. Individual written consent was sought from the healthcare workers regarding participation and recording of the interviews and observations. We obtained verbal consent from the patients and/or their relatives about observing the care they received along the pathways.

It was explained that anonymity would be ensured regarding the use of the transcribed interviews, observations, and voice notes in the patient journeys. We explained to the participants that all the audio recordings pertaining to the walkthroughs would be deleted once the recordings were transcribed. Only participants who gave informed consent were recruited in this study.

Data collection

In-depth Interviews (IDIs)

We used a semi-structured interview guide (Appendix 2) to conduct a total of 12 in-depth interviews (IDIs) with 11 frontline healthcare workers – nurses, clinical officers, medical officers, and administrative staff. These interviews were conducted virtually via Microsoft Teams and phone calls owing to the COVID-19 restrictions regarding face-to-face meetings. The interviews, averaging 43 minutes, were audio recorded and saved onto password-protected cloud storage database managed

For peer review only - http://bmjopen.bmj.com/site/about/guidelines.xhtml

by the KEMRI (Kenya Medical Research Institute)-Wellcome Trust Research Programme (KWTRP). All audio recordings were transcribed verbatim and were deleted after transcription.

Process mapping and Patient journeys

Patient journey mapping is a deliberate patient-oriented approach to better understand barriers, facilitators, experiences, interactions with services and/or outcomes for individuals and/or their caregivers and family members as they enter, navigate, experience, and exit service points in a health system by documenting elements of the journey to produce a visual or descriptive map (Davies et al., 2023; Sijm-Eeken et al., 2020).

The patient journey approach has been applied in patient safety to analyse the care service system and support human centred design (Simonse et al., 2019a; Trbovich & Vincent, 2019). The approach focuses on the sequence of care from the patient's perspective and allows for the examination of discrete patient interactions with providers at each encounter (Simonse et al., 2019a). This can be used to identify gaps that might otherwise go unnoticed.

The observer – the lead author OO – a research assistant with background training in nursing, conducted the journey mapping during a health facility assessment (HFA) of the four hospitals for the larger POETIC-COVID project. He first identified eight frontline healthcare workers (two per hospital) working in the emergency departments and through informal conversations using scenario-based questions, mapped the journey of a hypothetical critically ill patient when they arrived in the hospitals form the healthcare workers' perspective. OO drew the process maps as described by the health workers in illustrative flow diagrams (Appendix 3 & Appendix 4). The healthcare workers were asked to estimate the time taken at each step along the journey.

With the help of the staff at the emergency and outpatient departments, the observer then purposively selected and followed 7 critically ill patients as determined by their signs and symptoms from the time of arrival at the Outpatient/Emergency department to the point of admission into the wards or referral out to another facility. The observer noted the care provided and periods of inactivity against the time. Using a Dictaphone, the observer made real-time onsite audio commentary of the steps and activities against the time performed and this data was used to write out each patient journey (Holden & Carayon, 2021; Simonse et al., 2019b).

To explore Third Delay issues, we assessed the time taken in identification and administration of appropriate care for the selected patients. We used time to triage, time to diagnosis and time to management as proxies for determining Third Delay. *Time to triage* here referred to the time from patient's first contact with a healthcare worker (clinical officer, nurse, medical officer, or intern) to the time of departure to the next appropriate care point. Between this time and the time of an objective diagnosis was termed *time to diagnosis*. The time from when an objective diagnosis was made to the administration of appropriate care or intervention was defined as *time to initiation of care*.

Data analysis

The audio recordings and notes were transcribed verbatim in English then deidentified and imported into NVIVO® 12 Plus to generate nodes and to enable thematic analysis (Fereday & Muir-Cochrane, 2008; Guest G & MacQueen N, 2012; Patton, 2002) using inductive coding and a code book was developed. Four of the authors – OO, TW, EM, and JM – participated in coding transcripts. Separately at first, we used inductive coding to determine fundamental problems in the care critically ill patients currently. Next, we compared the themes with the Delphi that defined EECC backed with clinical input from the team to identify more EECC-appropriate information. We then developed a new code book reflecting both the development of the grounded themes and the EECC themes. Transcripts were then recoded using our new framework.

For peer review only - http://bmjopen.bmj.com/site/about/guidelines.xhtml

Since the interviews were primarily designed to answer the objectives of the POETIC-COVID project, we only abstracted data relevant to the domains in the Third Delay. These were statements speaking to equipment, space, infrastructure, administrative processes, staffing, skills, triage, and diagnostics. The IDI findings were used to build on the observations during the actual patient journeys. For this study, we used transcripts from 12 IDIs out of the total 17.

We used the narrative form of the patient journey to further develop and test our analytical framework for the IDIs on the provision of care in critical illness. We initially read the patient journeys to provide context and further depth in IDIs.

Patient and public involvement statement

The study design was informed by contemporary experiences of patients in Kenyan public hospitals, especially in light of the COVID-19 pandemic. Throughout the recruitment and conduct of the study, informed consent from patients and/or their relatives was maintained.

We held three feedback meetings with the hospitals after completion of data collection where we shared our findings and checked if our interpretation of the data made sense to hospital staff. Two of these (Facility 1 and Facility 2) were held in-person at the respective facilities, while one (Facility 3) was held virtually via Microsoft Teams. Facility 4 was unable to take part due to their COVID-19 workload. The audiences consisted of healthcare staff from various departments including accident & emergency, outpatient, surgery, paediatrics, medical ward, laboratory, radiology, nutrition, and hospital administration. The teams confirmed that our findings were a fair representation of their respective hospitals.

For peer review only

For peer review only - http://bmjopen.bmj.com/site/about/guidelines.xhtml

RESULTS

Themes

The main findings identified were ineffective identification of critical illness, inadequate resources for care, staffing shortages, interruptions from administrative processes, and lack of maintenance of EECC during patient flow. We combined these into three themes: suboptimal systems for identification of critical illness, inadequate resources for initiation and continuity of essential care, and disruption of the flow of care. These findings are summarised in the theoretical model we developed in this study (Figure 3).

Table 1: A sample patient journey from the perspective of healthcare workers at Hospital 1 (this is a hypothetical patient who ends up needing CT scan)

Step	Activity	Minimum time (minutes)	Maximum time (minutes)
1	Patient arrives in the casualty department		
2	Patient is received by the triage nurse	1	10
3	Preliminary vital signs are taken by the nurse	1	2
4	Patient waits in the queue at the registration window	1	30
5	Confirmation of patient health cover/ payment of registration fee	2	5
6	Patient registration	5	10
7	Waiting in the queue for vital signs checking	1	30
8	Vital signs repeated and recorded by nurse	2	5
9	History taking by nurse	5	20
10	Waiting in the queue for review by clinician	5	45
11	History taking by clinician	5	15
12	Examination by clinician	5	15
13	Investigations (CT scan) ordered	2	2
14	Transport outside facility to seek the required investigation	15	60
15	Waiting in queue for investigation	30	120
16	Waiting for interpretation of results	15	60
17	Travel back for review with CT Scan results	15	60
18	Waiting in queue for review with results	15	45
19	Doctor's review with results	10	45
20	Admission by nurse: nurse calls receiving ward for bed space	5	30
21	Waiting for availability of bed space	5	15
22	Nurse transports patient to destination ward	5	25

Consultation protocol
- Clinical officer (+/- clinical officer interns) sees patient
- Clinical officer calls Medical Officer (MO)/Registrar
- MO/Registrar calls consultant

Investigations
- Available tests: RBS, FHG, UECs, LFTs, Ultrasound, X-Ray, MRI (upon booking)
- Not available: CT-Scan, OGD

Referral protocol
- Through respective specialties;
  - Consultant reviews patient, decides referral
  - Unit manager notified
  - MO/ Registrar calls destination hospital with patient details
  - Ambulance set up and patient referred

The table above describes the steps from a healthcare worker's perspective of a hypothetical patient presenting to the Accident & Emergency department, typically as they navigate the various care points across the hospital departments of one of the study hospitals. The minimum and maximum time are estimates from the healthcare workers' point of view. More patient journeys are attached in the appendices (Appendix 5).

For peer review only - http://bmjopen.bmj.com/site/about/guidelines.xhtml

Suboptimal systems for identification of critical illness

Informal triage systems (time to triage)

Context: *A County Referral Hospital. The A&E (ER) unit has five nurses on the shift – although one of them is the unit manager or 'in-charge' and, as such has duties other than frontline care. The unit also has two Clinical Officers and one Medical Officer. The Emergency Room (ER) has five couches, four oxygen ports, and there are two bedside monitors that are operational.*

An adult female patient arrives at **1020h**, accompanied by two relatives, one of whom is her sister. **The nurse takes a brief history and identifies symptoms of severe abdominal pains**. **Four minutes after the patients' arrival, the nurse takes vital signs** and the patient's SPO_2 is noted to be 93% on room air, and she has a slightly elevated heart rate of 90 beats per minute.

Twenty minutes later, the nurse takes her blood pressure measurement, and it is noted to be **93/50** mmHg. The patient is also found to be clinically pale. The nurse sets up an intravenous infusion of normal saline. At this point, the **clinician has ordered a laboratory request** for a full hemogram, but the sample is not delivered to the laboratory until the patient's relatives **make payments for the laboratory investigations – at 1204h, nearly two hours after she arrived**.

Over two hours after the initial vital signs, the Medical Officer reviews the patient and orders an abdominopelvic ultrasound. The **relative joins the queue at the payment window to make payments** for the investigation. The patient, in a wheelchair, then joins the queue at the radiology department.

At 1345h, a staff member at the radiology department **sends the patient's relative back to the A&E to communicate that the test cannot be done at the moment** because the patient is supposed to have fasted. The back-and-forth between these two departments continues until **1415h** when the doctor at the A&E orders specifically for a pelvic ultrasound. This is now four hours after the patient arrived.

Afterward, the patient is wheeled back to the radiology department and the **relatives queue up at the payment window to update payments for the new test.** The patient remains in the queue until 1733h when she is wheeled into the ultrasonography room. A diagnosis of **ruptured ectopic pregnancy** was made, and the patient was admitted **12 hours later**, at 2230h, for an 'urgent' surgical intervention: an **exploratory laparotomy**.

Box 1: Case 1 - A female patient presenting with progressive abdominal pains at Facility 1 (later diagnosed with ruptured ectopic pregnancy)

All four hospitals had a designated area for triage of patients as they arrive at the Casualty (Accident & Emergency) and Outpatient departments. Overall, all the facilities had processes to appraise a patient's vital signs – temperature, blood pressure, pulse rate, and oxygen saturation levels – and general status within twenty minutes of arrival (Table 1, Box 1, Box 2) at the Emergency departments, although they lacked routines and systems for identification and continuous patient monitoring after the initial evaluation.

> "Okay for that one [triage] it's not really clear. It is not clear; there is a no systematic triaging." – **Clinical Officer, Facility 1**.

At Facility 1, for example, upon entry into the facility, the patients were directed into the tent where they waited to be seen – mostly on a first-come-first-served basis. The tent was located away from the general outpatient and emergency departments and was set up at the advent of COVID-19 to screen patients. The tent was reported to be congested with no specific guidelines governing the categorization of these patients in the tent.

For peer review only - http://bmjopen.bmj.com/site/about/guidelines.xhtml

"[…] there is delayed care because […] you know you have to remain in the triage until you're investigated for COVID at times there are no swabs, so you'll remain there. You see, the lab is far so even when they get their samples they have to go to the lab, back again, take care of other patients along the way you find that the patients can stay outside the tent for even twelve hours so there is delayed care." **Clinical Officer, Facility 1**.

While the hospitals had allocated physical space – room or cubicle – in the emergency and outpatient departments for triage, time to triage was markedly increased and effective triage practice was mostly postponed or forfeited altogether. Triage was largely informal, and the flow of patients was dependent on the availability and number of the staff and resources, as facilities adopted routines that worked most conveniently for these understaffed departments. The stretched capacities in space and staff-to-patient ratios further compounded the effects of ineffective triage.

"Ok in the triage system, sometimes you find that when we the staffs, there is shortage of staff. Like say you are alone for example like in the paediatric casualty; our acute room is a bit far from where we do triage so when you're handling a patient in the acute room and there is nobody at the triage so you might miss out identification of a critically ill patient in the queue so such scenario, we might end up losing a patient *akiwa kwa* (while still on the) queue." – **Nurse In-charge, Paediatric Department, Facility 2**.

With the overwhelming numbers of critically ill patients presenting in the emergency departments in the hospitals, Facility 3 activated a functional formal triage system, adopting the South African Triage System (SATS) in the Emergency Department, using colour-coding – red, orange, yellow and green – to classify patients upon arrival.

"Initially we didn't have a proper triage system, but we brought it up that we needed a proper triage system and for the past I think three or four days we've been having a proper triaging system, we have like the triage sheet where you see and then we have the list of patients who are emergency patients who patients can wait and like that." **Medical Officer, A&E department – Facility 3**

"So, we are using the South Africa triage system whereby we have at least started coding the patients. We are using the red, orange, green and yellow. So, when a patient comes, we are using the triage chart, which is at the triage area…so we follow that triage, we fill everything and any patient who appears to be red there is…we tag the patient then we take him or her to the emergency area." **Nurse, Facility 3.**

Delays in early recognition of condition (time to diagnosis)

For peer review only - http://bmjopen.bmj.com/site/about/guidelines.xhtml

Although the hypothetical journey map (Table 1) placed time to diagnosis within 35 minutes, findings from our actual patient journeys found that for tests available in the hospitals, it took 3-12 hours to make a clinical diagnosis after triage and initial evaluation. In-between the time the tests were ordered and when the results came back, the management of the patient was mainly symptomatic, with no system for continuous evaluation of patients' conditions (Box 1, Box 2, Box 3).

Besides being limited in physical space, the diagnostic departments were noted to be lacking variety of tests, occasioning profound delays and often necessitating patient referrals out of the hospitals to source the services. These can be seen in the hypothetical journey map, Case 1 & 2, and in the following quotes from IDIs.

> "We only have X-ray, and ultrasound. Yeah, so we don't have a CT scan yet…and MRI we don't have. If we want to do an MRI or a CT scan we have to send outside." – **Nurse In-charge, Paediatric outpatient department – Facility 2**.

> "Then the other thing is the laboratory. Our laboratory lacks many things; like there is no culture and sensitivity, those are things that miss in our lab. So, if we can have a bigger lab with many areas of assessment, it can be very much okay." – **Nursing Services Manager, Facility 1**.

With most hospitals having a centralised payment point, processing the laboratory and radiological tests ordered took considerable amounts of time. The delays were occasioned by long, inflexible registration and payment processes and long queues in these departments.

> "…there could be some delays depending with…maybe the patient needs a CT scan, you find the relative they don't have the money to do the CT scan or any other investigation which is not done with the hospital, so those are some of the challenges we get." **Nurse In-charge, A&E department – Facility 1**.

For peer review only

For peer review only - http://bmjopen.bmj.com/site/about/guidelines.xhtml

Inadequate resources for initiation and continuity of essential care

Resources and infrastructure contribution to delay

Context: *The unit has ED has 8 beds, all occupied at the time the patient is brought in. this department has 3 nurses, 1 Medical Officer, and 2 Emergency Medical Technicians (EMTs). The unit is full of patients: some are seated on chairs, others waiting outside on benches. The department lacks piped oxygen but has three oxygen cylinders with flowmeters attached (no flow splitters). The three cylinders are all in use at the time the patient is brought in.*

Four minutes after arrival, at 1210h, the nurse takes vital observations, and the SPO2 is **85%** on room air. At 1222h, the patient is placed on a bed, **but he's sharing the bed with another patient** since the space is full. The patient awaits review. No intervention since the initial vital observations at 1210h. Ten minutes later, the Medical Officer instructs EMTs to fix an IV line and draw blood samples for laboratory investigations.
At 1235h, the Medical Officer writes a laboratory request and hands the blood samples to the patient's **relatives to deliver to the laboratory upon making payment for the tests.** Twenty minutes later, a nurse sets up a 500ml bottle of IV Ringer's Lactate. The patient is yet to be reviewed.
At 1325h, the **patient has been noticed to be having convulsions**. We still haven't had a concrete review because the doctor is quite held up and the patient's details have not been updated. Fifteen minutes later, the Medical Officer prescribes an anticonvulsant. The **prescription is handed to a relative to pick up the drug** from the pharmacy **upon payment**. The unit is still very busy, with very sick patients being wheeled in. The **medication arrives one hour later**, at 1441h, and is administered six minutes later. All this while the patient has not yet been reviewed and is still in bed in status quo; still unresponsive and having convulsions. The results from the lab are not yet out.
At **1615h**, the results are yet to be received back from the lab. No further vitals have been taken. The patient's condition hasn't improved much, but the convulsions have subsided for the moment. The patient is yet to be reviewed.
The observer stopped the observation at 16:30h as his shift ended.

Box 2: Case 2 - The journey of an adult male patient received at the Emergency Department at Facility 2.

Although the vital observations were done within 20 minutes of patients' arrival at the emergency departments, subsequent monitoring was delayed or not done altogether. The reasons cited for this included limited physical infrastructure and shortage and/or failure of equipment. With the surge of patients needing constant monitoring during the COVID-19 pandemic, this proved to be a major challenge across the four facilities as illustrated in the contextual information of the patient journeys (Box 1, Box 2, Box 3) and the following IDI quotes.

> "Like we have only one monitor and we encounter many patients, and you see you cannot put this one [patient] on a monitor *umnyanganye uweke mwengine* (then you take it from them to connect to another patient). We have one monitor. The BP machines are also limited they are like two. The equipment is there but we cannot say it's enough." **– Nurse, Facility 1.**

The few equipment available were sometimes broken down and often shared between the many patients, and occasionally between departments. This further impacted negatively on patient monitoring and care and slowed down interdepartmental or interfacility transfers.

For peer review only - http://bmjopen.bmj.com/site/about/guidelines.xhtml

"...but sometimes you find the SPO_2 machine is not working, sometimes we don't have thermometers so we might miss out the degree of fever, so things like that. Sometimes they break down or they're stolen. Like the glucometer – you might travel with the patient but now if you can't check on the blood sugar level this patient might be in hypo[hypoglycaemic], but you'll fail to note." – **Nurse, Facility 3**.

Lack of essential commodities and consumables in the destination departments resulted in slow movement of patients from the outpatient and emergency departments, further slowing down the identification and management of incoming critically ill patients.

"I've seen only one point in Medical Ward. Oxygen is a challenge in the wards; most of the wards. And that is why you find patients of COVID stay here [Emergency Department] longer because they [wards] don't have piped oxygen like we, we have. They have to carry a cylinder. And right now, there is no oxygen." – **Nurse, Facility 3**.

As this was in the heart of the COVID-19 pandemic, in some cases, adaptive measures like referral and out-of-pocket purchase of these commodities were used to override the shortages and delays occasioned by unavailability of space and oxygen.

"Like recently, the last three four days we didn't have oxygen. So, you tell the patient to make an option where he or she can go to, she can be treated, either Facility 88 or Facility 99 [private hospitals], because we cannot handle them. In real sense, this is a government institution, whereby we are supposed to handle those COVID patients when they come." – **Nurse, Facility 3**.

Shortage of a skilled health workforce

Table 2 outlines the staffing structure across the departments in the four hospitals. Low staffing numbers were picked across departments in the four facilities. These data were collected in the health facility assessment (HFA) of these hospitals that was a preliminary stage of the POETIC-COVID study.

Table 2: staffing structure across the facilities during the visit (numbers represent one shift).
Key: N= Nurses, CO= Clinical officers, MO= Medical officers.*

* The Medical Officers across all facilities covered more than one department during their shift
** Multiple departments reduced their patient intake due to refurbishment work in Facility. Previous bed capacity for the medical and surgical departments was 32 beds each.

		Staff-to-patient ratios (day shift)			
**Department**	**Staff cadre**	Facility 1	Facility 2**	Facility 3	Facility 4
Accident & Emergency	N	4	5	3	2
	CO	1	4	0	2
	MO*	1	2	1	1

For peer review only - http://bmjopen.bmj.com/site/about/guidelines.xhtml

Obstetrics	N	10:12	8:37	9:73	2:19
	CO	2:12	2:37	0	0
	MO*	2:12	5:37	2:73	1:19
Paediatric ward	N	4:48	4:25	3:34	1:22
	CO	5:48	4:25	2:34	1:22
	MO*	2:48	3:25	1:34	2:22
Medical wards (male + female)	N	8:45	4:6	8:35	2:15
	CO	4:45	4:6	0	0
	MO*	2:45	4:6	2:35	1:15
Surgical wards (male + female)	N	8:50	3:8	8:51	3:12
	CO	2:50	3:8	0:51	0:12
	MO*	1:50	4:8	1:51	1:12
ICU	N	3:1		9:5	
	CO	1:1		1:5	
	MO	1:1	-	0	-

Across the hospitals, human resource shortage was a major hinderance to implementation of care interventions, and the available few staff could not allocate adequate focus to the critically ill patients in these departments.

> "The other thing I would require staff who are very well trained. We would require protocols and all staff need to be trained about these protocols. That if a patient presents with this symptom, what next? You know, people don't just walk around without you know, knowing what to do." – **Medical Officer, Facility 4**.

The facilities lacked an elaborate training system for staff on emergency and critical care, and since most of the staff train on the job, it was difficult to keep track of staff readiness to provide care to the critically ill patients.

> "And also, many of us, you see we have not been trained all of us about the BLS and ACLS, those ones. Not all of them have been trained; so, if we can get any update training and motivation, that is, it can be very good. Because once you have knowledge, the skills and the technique, those are the three key things you'll need for production." – **Nursing Services Manager, Facility 1**.

In addition, training and retention of skill-specific workforce was picked as a challenge across the hospitals. Constant staff rotations compounded this skill dilution as the trained staff were moved to other departments without replacement of the skillset.

For peer review only - http://bmjopen.bmj.com/site/about/guidelines.xhtml

> "They keep on changing, rotations…You were in this department, next month you're taken to another department. So you find that you can be trained in handling emergencies and…you're taken to another department so whoever is replacing you has not been trained so things like that." **– Nurse, Facility 2.**

Sometimes the strained staffing capacities led to coping measures such as rationing of care even seriously ill patients, in order to spread out the care to as many patients as possible.

> "In the wards there is also shortage, so they just check in general, and they if they deteriorate there is resuscitation and they make it, sometimes they don't make it" **Nurse, Facility 1**

Disruption of the flow of care

The role of administrative processes in delay – registration and payments

Context: *The Emergency Room (ER) and the general outpatient are housed under one roof. The unit has one nurse and one Medical Officer (MO) and is full of patients waiting to be seen. The nurse reported at 7:30am and will oversee this unit until 12:30pm when another nurse will come. The medical officer is also covering theatre in case of any surgical emergencies. The ER has three [3] beds/couches for patients, and at the moment, all three beds are occupied. There are also a few nursing students assisting in the care of these patients. Also, two Clinical Officers; one here in the emergency room, the other one covering the outpatient section. The unit has one functional port for piped oxygen which is currently in use. The ER is full. There's a COVID-19 isolation ward but currently that is full as well. There's no departmental phone.*

An elderly male patient – a referral on follow-up for COVID-19 pneumonia – arrives at Facility 4 at **0857h** with symptoms of severe breathing difficulty, although the SPO_2 levels were above 95% on arrival. The patient is kept in the Emergency Department, awaiting allocation of space in the COVID-19 isolation ward at the Facility.
At **1217h**, the Medical Officer reviews the patient and noted the patient to be desaturating; SPO_2 as low as **84%. There's only one oxygen port at the ER, and at the moment it's in use by another patient**. The MO calls another facility, Facility X (a regional referral facility), since the isolation unit at Facility 4 is full. Facility X responds with a promise to call back once a space avails.
At **1252h** the SPO_2 levels are noted to be below **80%** and the patient is put on oxygen via face mask. The space at Facility X has not been confirmed yet. There's a back-and-forth between the medical officer and Facility X until **1543h** when the space in the isolation unit at Facility X is confirmed, and the ambulance is set up to ferry the patient.

Box 3: Case 3 - Journey of an adult male patient brought in with symptoms of severe difficulty in breathing at Facility 4

The hospitals lacked systems to override payments for tests, investigations, and essential services for critically ill patients, leading to delays in receipt of adequate and appropriate treatment by patients. Payment for registration, laboratory investigations, and radiological tests were a barrier to quality of care and smooth flow of patients between care points. Prescribed tests and investigations were not processed until the patients, or their relatives paid for these services and in between, care was often withheld while the payments were sorted out.

> "The issue that we have is this issue of funds. A patient may come to you presenting with hypoglycaemia and you don't know…it's an altered mental status. You really don't

For peer review only - http://bmjopen.bmj.com/site/about/guidelines.xhtml

> know, because the…the relatives have to pay for the RBS [random blood sugar]. You understand?" – **Medical Officer, A&E department – Facility 4**

Staff in some facilities felt that it would help if there were systems to hold off payments for basic tests until later when the results are out, and the patient has been attended to.

> "I would require that we have a very clear path with the lab so that we don't require patients to pay out for things like RBS, you know. Essential tests like BS [blood smear] for P. falciparum. So that in that clear path patients can always pay for these things later once, once they get well or whether they make it, or they don't make it" – **Medical Officer, A&E department – Facility 4**

Some facilities reported having a waiver process, through the office of the hospital Social Worker, if a patient is unable to pay for a test or investigation. However, even in the facilities that have waiver systems for patients who cannot afford a service, the processes were often very slow and unreliable in the event of critical illness.

> "So, the challenge is *pesa* (money)…money, and you see the patient cannot be waived, nowadays, you have to pay cash. And when you go to the social worker, what he'll tell you is that he or she can waive to a certain point – not beyond maybe I don't know, 2000 [Kenyan shillings], not beyond 3000. That is now the challenge." – **Nurse In-charge A&E department – Facility 3**

Inefficient communication systems

We identified ineffective communication systems as a major limitation to reducing the *time to initiation of care* – one of our indicators for Third Delay - of critically ill patients. Lack of direct interdepartmental communication lines was picked across all four hospitals. Most departments lacked a functional departmental telephone, and communication between departments was largely through the health workers' personal mobile phones (Box 3).

> "In fact, I was forgetting. That is a very big problem. We don't have communication equipment. It used to be there but *ziliharibika zote* (they all broke down) so right now we use our phones […] It's very difficult because you have to call personal phones. Somebody can even ignore." **Nurse, Facility 2.**

In the four facilities, communication generally relied on the office of the Covering Nurse even in emergencies. This office serves as a call centre for the hospital to provide liaison between departments and to link the hospital with other facilities for inbound and outgoing referrals. In every shift, there's a designated nurse who takes up the role, with a designated facility phone. The covering nurse receives and relays information between the relevant personnel or departments, including the administration when needed. With absence of EECC and lack of processes to maintain care in-

For peer review only - http://bmjopen.bmj.com/site/about/guidelines.xhtml

between, the protocol for escalation of care was also described as long and time-consuming, especially during night shifts and weekends.

> "The nurse now has to look for the nurse covering and tell the nurse covering that we have a patient, and this patient is to be reviewed by a medical officer. Then the nurse covering calls the medical officer, at the same time the nurse covering calls the driver… At times it takes one hour, two hours, three hours. And sometimes the medical officer is within the hospital yes but doing an operation. So, it means the patients have to wait until the operation is over…That is what happens in the night. In…over the weekend, again there is that delay." – **A&E Nurse, Facility 4.**

When probed about the possibility of a better and more prompt communication system, some of the facilities reported that it would be a welcome upgrade, although budgetary constraints would cripple the implementation and sustainability of such a system.

"…Maybe if that one [communication system] can be put in place will be able to help our patients" **Nurse, Facility 2.**

In some cases, the faulty communication hardware meant that the staff had to walk to other departments to relay the information in person when planning on transferring patients to the wards from the emergency department. This is not only time-consuming, but also takes away valuable manpower from the already understaffed units.

> "And it's…it's also difficult because of shortage every time I keep on walking in the ward, *"Naleta mgonjwa"* (I'm bringing a patient), I'm bringing a patient, you know, it's also cumbersome." – **Nurse In-charge A&E department – Facility 3**

In general, *time to initiation of care* was between 4-12 hours. In Case 1, for example, a follow-up call the following day revealed that the patient was admitted past 10:30 pm and prepared for an emergency surgical intervention, although the patient had arrived at the facility more than 12 hours prior.

For peer review only - http://bmjopen.bmj.com/site/about/guidelines.xhtml

DISCUSSION

We qualitatively examined the patient monitoring and evaluation processes and the limiting factors in the journey of critically ill patients to identify delays in the delivery of care when seeking care in Kenyan secondary-level public hospitals. We found that while the hospitals had allocated physical space in the emergency and outpatient departments for triage, *time to triage* was markedly increased and effective triage practice was mostly postponed or forfeited altogether. Our study also identified inadequate resources, ineffective communication systems, and administrative processes – non-clinical business- and protocol-related organizational processes – as the major limitations to reducing the *time to diagnosis* and *time to initiation care* to critically ill patients within low-resource hospital settings.

Shortage of material resources and acute shortage of human resources due to low employment rates and high staff turnovers greatly affect both initial and subsequent evaluation and review of patients, as the healthcare workers focus on clearing the overwhelming queues with the available resources in these resource-limited hospital settings (Downar et al., 2022; Floyd et al., 2018; Knight et al., 2013; Morof et al., 2019). The inadequacies not only directly affect care to patients negatively – as seen in the lengthy delays in triage, diagnosis, and definitive management – but they also have forced cyclic adaptive mechanisms that further constrain the capacity of the hospitals to improve. All these culminate into a marked Third Delay in patients seeking care in these hospitals, and in the case of critically ill patients, the delays could be detrimental to their clinical outcome and lead to preventable mortality. Measures such as a package of interventions focussing on adequate priority infrastructure and medical supplies, prioritizing steps such as triage and stabilization of critically ill patients, sufficiently-trained health care providers, evidence-based care, and referral capacity to support transfers to higher-level care (Morof et al., 2019; Schell et al., 2021) are therefore key in addressing Third Delay in hospitals in resource-limited settings.

The practicality of inter-disciplinary linkages remains an evident challenge in public hospitals in resource-constrained settings. For critically unwell patients, functioning linkage and communication systems are essential owing to the time-critical nature of their conditions, and the fact that they often cannot advocate for themselves. While it is vital to account for all the services offered to patients in the facilities, there should be straightforward systems in place to ensure that the payments, or lack thereof, do not interfere with the care delivered to critically ill patients and that care is not withheld when the patient is not able to raise the amounts required, or when the payments are yet to be processed. In addition, although literature illustrates that patient-specific information, particularly in the emergency departments, is an essential component of the patient pathway, patient-physician relationship and nursing practice and team working (Cohen et al., 2013), the process of obtaining and availing required patient details should not impede the initiation and/or flow of care especially in critical illness. Reducing the turn-around-time in emergencies is key to favourable prognosis, therefore eliminating these administrative stumbling blocks would help reduce the congestion in the emergency care pathways and well as capitalize on the cost-effectiveness of the basic emergency care interventions (2006; Jayaraman et al., 2009; Shah et al., 2023; Shanahan et al., 2018).

In chaos, the risks are profound. COVID-19 was pivotal to illuminating areas for improvement in ill-prepared hospital systems globally. In summary, the findings from our study speak to a series of dysfunctional systems for care with adaptive mechanisms that are unideal for the care of critically ill patients. Implementing a guiding principle that the most time-critical, life-saving, and feasible care is the priority when resources are scarce might be a helpful starting point in improving quality of care and outcomes hospitals. Such a policy, as articulated in the recent national strategic plan for implementing EECC in Tanzania (MOH, 2022) could be useful for the change needed to address defective triage, improper initial and continued evaluation of critically ill patients, strained resource capacities, and administrative bottlenecks that all impede the flow of care for critically ill patients

For peer review only - http://bmjopen.bmj.com/site/about/guidelines.xhtml

while seeking care in these secondary-level public hospitals. We believe that examining critical illness care through a Third-delay lens would allow us to develop better targeted interventions that improve the likelihood of receiving essential and timely critical illness care services. Figure 3 summarises our concept for the causes of Third Delay in care of critically ill patients.

CONCLUSION

Public hospitals in Kenya have long-standing ineffective intra and inter-facility systems that are unreliable in the care of critically ill patients when they present in hospitals. Care of critically ill patients – identification, monitoring and decision making – requires prompt systems to identify and communicate the urgency across multiple care points. Eliminating Third Delay using a package of prompt essential care is therefore an important and time-critical measure to improve the care outcomes of critically ill patients in resource-constrained settings. Findings from our study can help emphasize the need for standardised effective and reliable care priorities to maintain of care of critically ill patients within hospitals.

For peer review only

For peer review only - http://bmjopen.bmj.com/site/about/guidelines.xhtml

ACKNOWLEDGEMENTS

We are grateful to Prof. Claudia Hanson, Senior Lecturer, Karolinska Institutet, Sweden, Professor, London School of Hygiene & Tropical Medicine, UK and Dr Roy Nobhojit, Surgeon and Researcher, BARC Hospital, HBNI University, Mumbai, India, for their invaluable mentorship that helped shape the concept and direction of this study.

We wish to thank the POETIC-COVID team and the entire EECC network for the immense support and teamwork throughout the study period and beyond. We would also like to acknowledge the contribution of the Ministry of Health – Kenya, the participating hospitals and their staff in the process of this study.

Contributorship statement

OO conceptualized and designed the study, acquired and analysed the data, and developed the first draft of the manuscript with assistance from TW, JO, and JMcK. TB, ME, and COS contributed to the conceptualization and design of the study. TW, EM, JM, KK, and OO analysed the data together. JO also contributed to acquisition of data and was the Scientific Lead for the larger POETIC-COVID study. All the authors interpreted the findings, critically revised the manuscript, and approved the final version.

Conflict of interest statement

The authors in this study all declare no conflicts of interest.

Funding Statement

This study was funded through the POETIC-COVID project by a grant from Wellcome Trust [221571/Z/20/Z] as part of the *Innovation in Low and Middle-income Countries* Flagship. ME is supported by a Wellcome Senior Fellowship (#207522) with further support for authors from a core grant awarded to the KEMRI-Wellcome Trust Research Programme (#092654).

Data sharing statement

Additional anonymised transcripts of patient journeys, process maps, and in-depth interviews are attached separately as supplemental material submitted alongside this manuscript. The raw data supporting this article will be availed by the authors whenever necessary.

Ethics approval statement

This study was part of a larger study approved by the KEMRI Scientific and Ethical Review Committee (SERU Number 4085) and London School of Hygiene and Tropical Medicine (REF 22 866). Institutional approvals were also obtained at hospital level and/or their relevant County liaison for all four hospitals.

For peer review only

For peer review only - http://bmjopen.bmj.com/site/about/guidelines.xhtml

REFERENCES

. (2006). In D. T. Jamison, J. G. Breman, A. R. Measham, G. Alleyne, M. Claeson, D. B. Evans, P. Jha, A. Mills, & P. Musgrove (Eds.), *Disease Control Priorities in Developing Countries*. The International Bank for Reconstruction and Development / The World Bank

Oxford University Press

Copyright © 2006, The International Bank for Reconstruction and Development/The World Bank Group.

Adhikari, N. K., Fowler, R. A., Bhagwanjee, S., & Rubenfeld, G. D. (2010). Critical care and the global burden of critical illness in adults. *The Lancet*, *376*(9749), 1339-1346.

Barnes-Josiah, D., Myntti, C., & Augustin, A. (1998). The "three delays" as a framework for examining maternal mortality in Haiti. *Social science & medicine*, *46*(8), 981-993.

Bell, M. B., Konrad, D., Granath, F., Ekbom, A., & Martling, C.-R. (2006). Prevalence and sensitivity of MET-criteria in a Scandinavian University Hospital. *Resuscitation*, *70*(1), 66-73.

Cavallaro, F. L., & Marchant, T. J. (2013). Responsiveness of emergency obstetric care systems in low-and middle-income countries: A critical review of the "third delay". *Acta obstetricia et gynecologica Scandinavica*, *92*(5), 496-507.

Cohen, E. L., Wilkin, H. A., Tannebaum, M., Plew, M. S., & Haley, L. L. (2013). When Patients Are Impatient: The Communication Strategies Utilized by Emergency Department Employees to Manage Patients Frustrated by Wait Times. *Health Communication*, *28*(3), 275-285. https://doi.org/10.1080/10410236.2012.680948

Dart, P. J., Kinnear, J., Bould, M. D., Mwansa, S., Rakhda, Z., & Snell, D. (2017). An evaluation of inpatient morbidity and critical care provision in Zambia. *Anaesthesia*, *72*(2), 172-180.

Davies, E. L., Bulto, L. N., Walsh, A., Pollock, D., Langton, V. M., Laing, R. E., Graham, A., Arnold-Chamney, M., & Kelly, J. (2023). Reporting and conducting patient journey mapping research in healthcare: A scoping review. *Journal of advanced nursing*, *79*(1), 83-100.

Downar, J., Smith, M. J., Godkin, D., Frolic, A., Bean, S., Bensimon, C., Bernard, C., Huska, M., Kekewich, M., Ondrusek, N., Upshur, R., Zlotnik-Shaul, R., & Gibson, J. (2022). A framework for critical care triage during a major surge in critical illness. *Canadian Journal of Anaesthesia*, *69*(6), 774-781. https://doi.org/10.1007/s12630-022-02231-2 (Un algorithme pour le triage aux soins intensifs lors d'une augmentation majeure des maladies graves.)

Fereday, J., & Muir-Cochrane, E. (2008). Demonstrating rigor using thematic analysis: A hybrid approach of inductive and deductive coding and theme development. *International journal of qualitative methods*, *5*(1), 80-92.

Floyd, L., Bryce, F., Ramaswamy, R., Olufolabi, A., Srofenyoh, E., Goodman, D., Pearson, N., Morgan, K., Tetteh, C., & Ahwireng, V. (2018). The introduction of a midwife-led obstetric triage system into a regional referral hospital in Ghana. *Midwifery*, *61*, 45-52.

Gabrysch, S., & Campbell, O. M. (2009). Still too far to walk: literature review of the determinants of delivery service use. *BMC pregnancy and childbirth*, *9*(1), 1-18.

Guest G, & MacQueen N. (2012). Introduction to Thematic Analysis. In *Applied Thematic Analysis*. SAGE.

Gupta, S., Khajanchi, M., Kumar, V., Raykar, N. P., Alkire, B. C., Roy, N., & Park, K. B. (2019). Third delay in traumatic brain injury: time to management as a predictor of mortality. *Journal of neurosurgery*, *132*(1), 289-295.

Holden, R. J., & Carayon, P. (2021). SEIPS 101 and seven simple SEIPS tools. *BMJ Quality & Safety*, *30*(11), 901-910. https://doi.org/10.1136/bmjqs-2020-012538

Hsia, R. Y., Thind, A., Zakariah, A., Hicks, E. R., & Mock, C. (2015). Prehospital and emergency care: updates from the disease control priorities, version 3. *World Journal of Surgery*, *39*(9), 2161-2167.

For peer review only - http://bmjopen.bmj.com/site/about/guidelines.xhtml

Jayaraman, S., Mabweijano, J. R., Lipnick, M. S., Caldwell, N., Miyamoto, J., Wangoda, R., Mijumbi, C., Hsia, R., Dicker, R., & Ozgediz, D. (2009). First things first: effectiveness and scalability of a basic prehospital trauma care program for lay first-responders in Kampala, Uganda. *PloS one*, *4*(9), e6955.

Kayambankadzanja, R. K., Schell, C. O., Warnberg, M. G., Tamras, T., Mollazadegan, H., Holmberg, M., Alvesson, H. M., & Baker, T. (2022). Towards definitions of critical illness and critical care using concept analysis. *medRxiv*.

Kenya, M. o. H. (2020). *Kenya_2019-Novel-Coronavirus-Contingency-Readiness-and-Early-Response-Plan-February-April-2020.pdf*. Accessed online Jan 2022 Retrieved from https://macdrive.mcmaster.ca/f/f71ef070747a488794eb/

Knight, H. E., Self, A., & Kennedy, S. H. (2013). Why are women dying when they reach hospital on time? A systematic review of the 'third delay'. *PloS one*, *8*(5), e63846. https://doi.org/10.1371/journal.pone.0063846

Kruisselbrink, R., Kwizera, A., Crowther, M., Fox-Robichaud, A., O'Shea, T., Nakibuuka, J., Ssinabulya, I., Nalyazi, J., Bonner, A., & Devji, T. (2016). Modified early warning score (MEWS) identifies critical illness among ward patients in a resource restricted setting in Kampala, Uganda: a prospective observational study. *PloS one*, *11*(3), e0151408.

Lowe, A., Norris, A. C., Farris, A. J., & Babbage, D. R. (2018). Quantifying thematic saturation in qualitative data analysis. *Field methods*, *30*(3), 191-207.

Maina, M., Tosas-Auguet, O., English, M., Schultsz, C., & McKnight, J. (2020). COVID-19: an opportunity to improve infection prevention and control in LMICs. *The Lancet Global Health*, *8*(10), e1261.

Mkumbo, E., Willows, T. M., Onyango, O., Khalid, K., Maiba, J., Schell, C. O., Oliwa, J., McKnight, J., & Baker, T. (2023). Same label, different patients: Health-workers' understanding of the label 'critical illness'. *Frontiers in health services*, *3*, 1105078.

MOH. (2019). Kenya Harmonized Health Facility Assessment (KHFA) 2018/2019. In: Nairobi MOH.

MOH, T. (2022). *National Strategic Plan on Essential Emergency and Critical Care Services (2023-2026). 2022*.

Molyneux, E., Ahmad, S., & Robertson, A. (2006). Improved triage and emergency care for children reduces inpatient mortality in a resource-constrained setting. *Bulletin of the World Health Organization*, *84*, 314-319.

Morof, D., Serbanescu, F., Goodwin, M. M., Hamer, D. H., Asiimwe, A. R., Hamomba, L., Musumali, M., Binzen, S., Kekitiinwa, A., & Picho, B. (2019). Addressing the third delay in Saving Mothers, Giving Life districts in Uganda and Zambia: ensuring adequate and appropriate facility-based maternal and perinatal health care. *Global Health: Science and Practice*, *7*(Supplement 1), S85-S103.

Palinkas, L. A., Horwitz, S. M., Green, C. A., Wisdom, J. P., Duan, N., & Hoagwood, K. (2015). Purposeful sampling for qualitative data collection and analysis in mixed method implementation research. *Administration and policy in mental health and mental health services research*, *42*, 533-544.

Patton, M. Q. (2002). Qualitative research & evaluation methods, Thousand Oaks Sage Publications. *New York, New Delhi, London*.

Pope, C., Ziebland, S., & Mays, N. (2000). Qualitative research in health care: Analysing qualitative data. *BMJ: British Medical Journal*, *320*(7227), 114.

Razzak, J., Usmani, M. F., & Bhutta, Z. A. (2019). Global, regional and national burden of emergency medical diseases using specific emergency disease indicators: analysis of the 2015 global burden of disease study. *BMJ global health*, *4*(2), e000733.

Roy, N., Kizhakke Veetil, D., Khajanchi, M. U., Kumar, V., Solomon, H., Kamble, J., Basak, D., Tomson, G., & von Schreeb, J. (2017). Learning from 2523 trauma deaths in India-opportunities to prevent in-hospital deaths. *BMC health services research*, *17*(1), 1-8.

Sasser, S., Kellermann, A., & Lormand, J. (2013). Prehospital trauma care systems. Geneva: World Health Organization; 2005. *Arh Hig Rada Toksikol*, *64*, 567-579.

For peer review only - http://bmjopen.bmj.com/site/about/guidelines.xhtml

Schell, C. O., Khalid, K., Wharton-Smith, A., Oliwa, J., Sawe, H. R., Roy, N., Sanga, A., Marshall, J. C., Rylance, J., Hanson, C., Kayambankadzanja, R. K., Wallis, L. A., Jirwe, M., Baker, T., Collaborators, E., & Collaborators*, E. (2021). Essential Emergency and Critical Care: a consensus among global clinical experts. *BMJ Glob Health*, *6*(9). https://doi.org/10.1136/bmjgh-2021-006585

Shah, H. A., Baker, T., Schell, C. O., Kuwawenaruwa, A., Awadh, K., Khalid, K., Kairu, A., Were, V., Barasa, E., & Baker, P. (2023). Cost Effectiveness of Strategies for Caring for Critically Ill Patients with COVID-19 in Tanzania. *PharmacoEconomics-open*, 1-16.

Shanahan, T., Risko, N., Razzak, J., & Bhutta, Z. (2018). Aligning emergency care with global health priorities. *International Journal of Emergency Medicine*, *11*(1), 1-4.

Sijm-Eeken, M., Zheng, J., & Peute, L. (2020). Towards a lean process for patient journey mapping–a case study in a large academic setting. In *Digital Personalized Health and Medicine* (pp. 1071-1075). IOS Press.

Simonse, L., Albayrak, A., & Starre, S. (2019a). Patient journey method for integrated service design [research-article]. *Design for Health*, *3*(1). https://doi.org/https://doi.org/10.1080/24735132.2019.1582741

Simonse, L., Albayrak, A., & Starre, S. (2019b). Patient journey method for integrated service design. *Design for Health*, *3*(1), 82-97. https://doi.org/10.1080/24735132.2019.1582741

Sorensen, B. L., Rasch, V., Massawe, S., Nyakina, J., Elsass, P., & Nielsen, B. B. (2011). Advanced life support in obstetrics (ALSO) and post-partum hemorrhage: a prospective intervention study in Tanzania. *Acta Obstet Gynecol Scand*, *90*(6), 609-614. https://doi.org/10.1111/j.1600-0412.2011.01115.x

Surgeons, C. o. T. o. t. A. C. o. (2006). Resources for optimal care of the injured patient 2006. *Chicago, IL: American College of Surgeons*.

Thaddeus, S., & Maine, D. (1994). Too far to walk: maternal mortality in context. *Social science & medicine*, *38*(8), 1091-1110.

Trbovich, P., & Vincent, C. (2019). From incident reporting to the analysis of the patient journey. https://doi.org/10.1136/bmjqs-2018-008485

Tuti, T., Bitok, M., Malla, L., Paton, C., Muinga, N., Gathara, D., Gachau, S., Mbevi, G., Nyachiro, W., Ogero, M., Julius, T., Irimu, G., & English, M. (2016). Improving documentation of clinical care within a clinical information network: an essential initial step in efforts to understand and improve care in Kenyan hospitals. *BMJ global health*, *1*(1), e000028. https://doi.org/10.1136/bmjgh-2016-000028

Vora, N., Maitra, N., & Pandya, P. (2021). Use of the Maternal Foetal Triage Index-Addressing the Third Delay in Obstetrics: An Observational Study. *Authorea Preprints*.

Zhao, Y., McKnight, J., & English, M. (2020). *Low-income countries' bids for World Bank funding raise serious concerns about their coronavirus strategies*. Retrieved 7th January from https://theconversation.com/low-income-countries-bids-for-world-bank-funding-raise-serious-concerns-about-their-coronavirus-strategies-138628

For peer review only - http://bmjopen.bmj.com/site/about/guidelines.xhtml

Figure Legends

1. Figure 1: The framework of the Third Delay model (adapted from Gabrysch & Campbell, and Cavallaro & Marchant). The loop represented by the green connectors illustrates the continuous re-assessment and monitoring of acutely ill patients needed to identify critical illness. The loop represented by the orange dotted connectors represents a second referral to a higher-level facility. Any complications that cannot be managed at the first facility should be promptly diagnosed and referred within the shortest time possible to eliminate **second emergency delay**.

2. Figure 2: Organization of the Kenyan public health system

3. Figure 3: An empirical concept of the causes of Third Delay in the care of patients presenting with clinical illness.

For peer review only

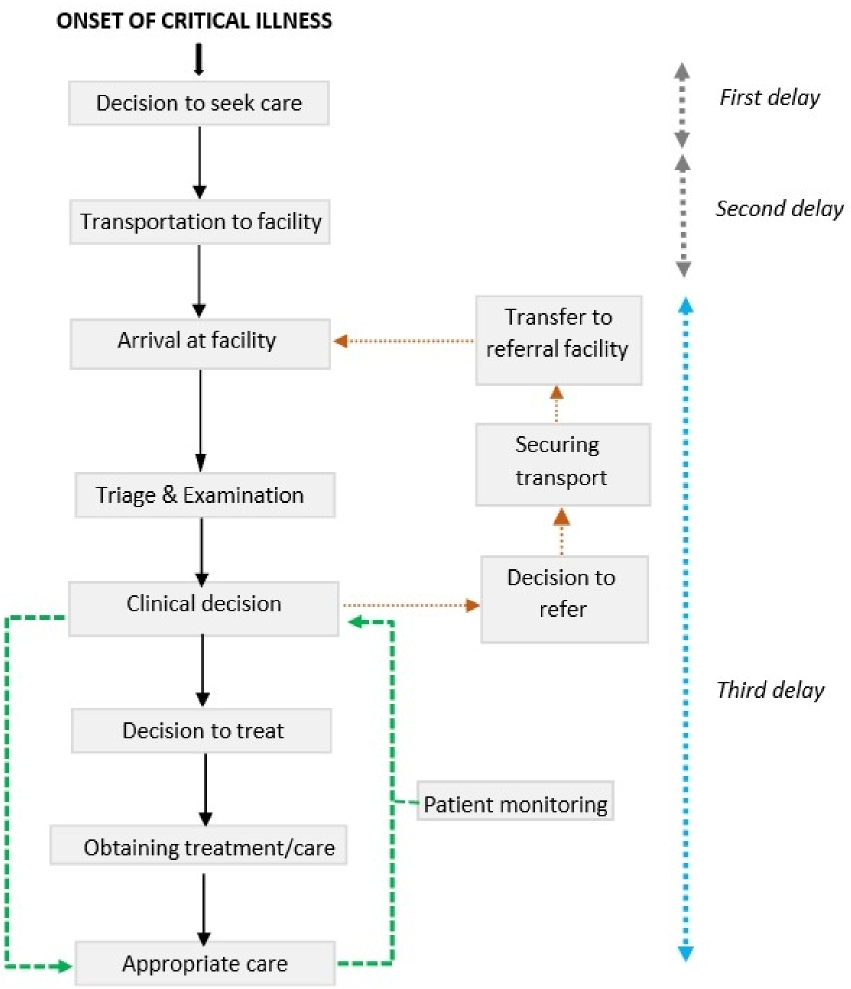

The framework of the Third Delay model (adapted from Gabrysch & Campbell, and Cavallaro & Marchant). The loop represented by the green connectors illustrates the continuous re-assessment and monitoring of acutely ill patients needed to identify critical illness. The loop represented by the orange dotted connectors illustrates a second referral to a higher-level facility. Any complications that cannot be managed at the first facility should be promptly diagnosed and referred within the shortest time possible to eliminate second emergency delay.

146x155mm (120 x 120 DPI)

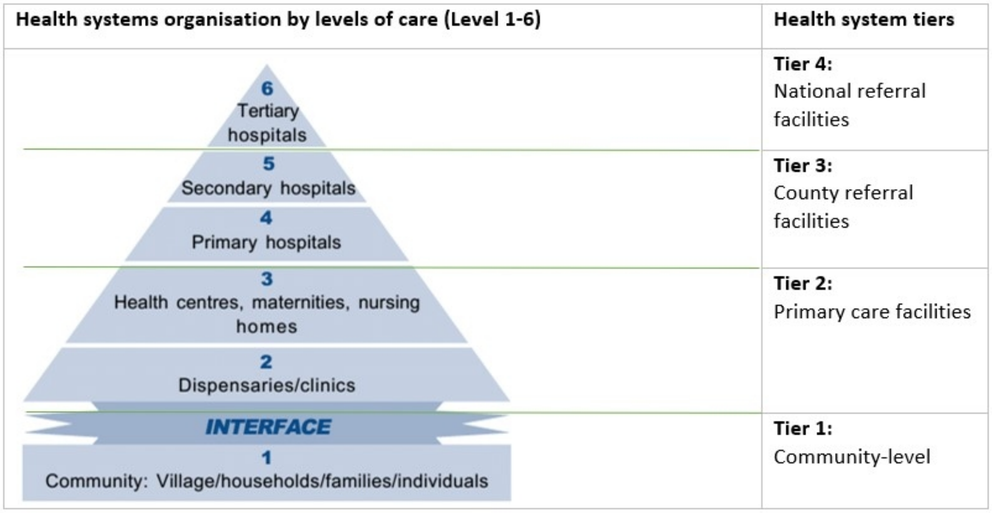

Organization of the Kenyan public health system by levels of care

170x87mm (120 x 120 DPI)

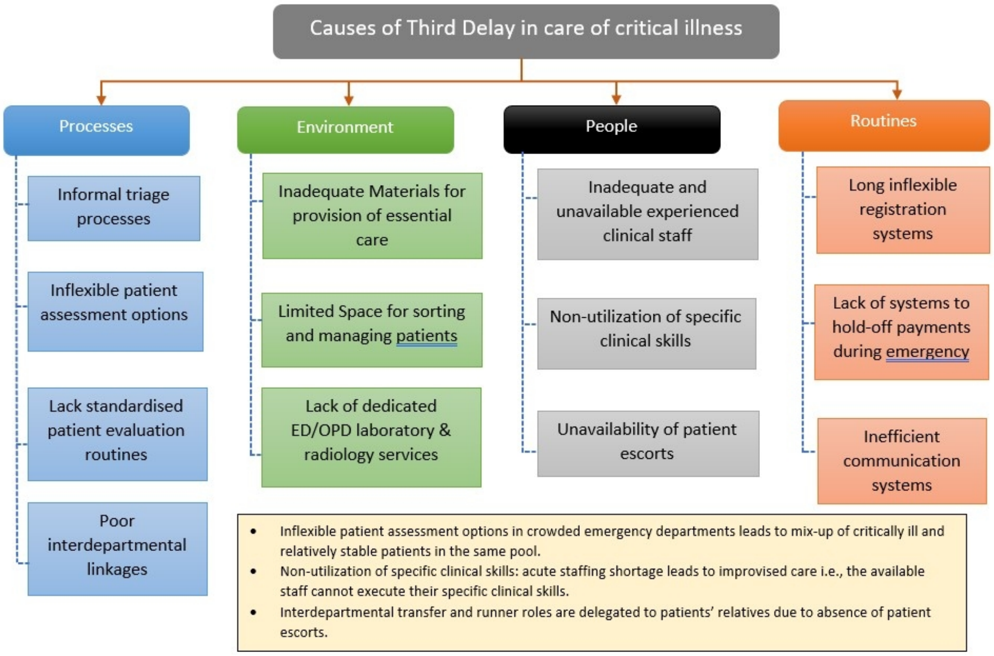

A theoretical concept of the causes of Third Delay in the care of patients presenting with clinical illness

251x165mm (96 x 96 DPI)

APPENDICES

Appendix 1: EECC checklists

PANEL 2. The Hospital Readiness Requirements for Essential Emergency and Critical Care

IDENTIFICATION OF CRITICAL ILLNESS

The following items are required for a hospital to be ready for the identification of critically ill patients:

CATEGORY	ITEM
1.1. EQUIPMENT	1.1.1 Clock with second hand<br>1.1.2 Pulse oximeter & probe<br>1.1.3 Blood pressure measuring equipment (eg. sphygmomanometer with a stethoscope)<br>1.1.4 Blood pressure cuffs of different paediatric and adult sizes<br>1.1.5 Light source (lamp or flashlight)<br>1.1.6 Thermometer
1.2 CONSUMABLES	1.2.1 Soap or hand disinfectant<br>1.2.2 Examination gloves
1.3 DRUGS	None
1.4 HUMAN RESOURCES	1.4.1 Health workers with the ability to identify critical illness 24h/day
1.5 TRAINING	1.5.1 The health workers are trained in the identification of critical illness
1.6 ROUTINES	1.6.1 Routines for the identification of critical illness
1.7 GUIDELINES	1.7.1 Guidelines for the identification of critical illness
1.8 INFRASTRUCTURE	1.8.1 Designated triage area (area for the identification of critical illness) in the Out-Patient Department or Emergency Unit (area of the hospital where patients arrive)<br>1.8.2 Running water

only

PANEL 2 continued ...

CARE OF CRITICAL ILLNESS

The following items are required for a hospital to be ready to provide the care of critically ill patients:

CATEGORY	ITEM
2.1 EQUIPMENT	2.1.1 Suction machine (electric or manual)<br>2.1.3 Oxygen supply 24h/day (cylinder, concentrator (with electricity supply) or piped oxygen)<br>2.1.4 Flow meter (if using cylinder or piped oxygen)<br>2.1.5 Leak-free connectors from oxygen source to tubing<br>2.1.6 Bag Valve Mask (resuscitator) – neonatal, paediatric and adult sizes<br>2.1.7 Sharps disposal container<br>2.1.8 External heat source
2.2 CONSUMABLES	2.2.1 Suction catheters of paediatric and adult sizes<br>2.2.2 Guedel airways of paediatric and adult sizes<br>2.2.3 Pillows<br>2.2.4 Oxygen tubing<br>2.2.5 Oxygen nasal prongs<br>2.2.6 Oxygen face masks of paediatric and adult sizes<br>2.2.7 Oxygen face masks with reservoir bags of paediatric and adult sizes<br>2.2.8 Masks for Bag Valve Mask (resuscitator) – neonatal, paediatric and adult sizes<br>2.2.9 Compression bandages<br>2.2.10 Plasters or tape<br>2.2.11 Gauze<br>2.2.12 Intravenous cannulas of paediatric and adult sizes<br>2.2.13 Intravenous giving sets<br>2.2.14 Skin disinfectant for cannulation<br>2.2.15 Syringes<br>2.2.16 Nutrition<br>2.2.17 Nasogastric tubes<br>2.2.18 Lubricant for nasogastric tube insertion<br>2.2.19 Intramuscular needles<br>2.2.20 Intraosseous cannulas of different sizes<br>2.2.21 Blankets<br>2.2.22 Facemasks for Infection Prevention and Control<br>2.2.23 Aprons or gowns<br>2.2.24 Charts/notes for documentation<br>2.2.25 Pens
2.3 DRUGS	2.3.1 Oral rehydration solution<br>2.3.2 Intravenous crystalloid fluids (eg. normal saline or Ringer's Lactate)<br>2.3.3 Intravenous dextrose fluid (eg. 5%, 10% or 50%)<br>2.3.4 Oxytocin<br>2.3.5 Adrenaline<br>2.3.6 Appropriate antibiotics<br>2.3.7 Diazepam<br>2.3.8 Magnesium sulphate<br>2.3.9 Paracetamol<br>2.3.10 Local anaesthetic (eg. 2% lignocaine) (eg. for intraosseous cannulation)
2.4 HUMAN RESOURCES	2.4.1 Health workers with the ability to care for critically ill patients 24hrs/day<br>2.4.2 Senior health worker who can be called to assist with the care of critically ill patients 24hrs/day

PANEL 2 continued ...

2.5 TRAINING	2.5.1 The health workers are trained in the care of critically ill patients
2.6 ROUTINES	2.6.1 Routines for managing critically ill patients<br>2.6.2 Routine for the provision of EECC without taking into account patients' ability to pay<br>2.6.3 Routines for who and how to call to seek senior help 24hrs/day, 7 days/week<br>2.6.4 Routines for integrating EECC with other care including the definitive care of the underlying condition (eg. use of condition-specific guidelines)
2.7 GUIDELINES	2.7.1 Guidelines for the essential care of critically ill patients
2.8 INFRASTRUCTURE	2.8.1 Designated space for the care of critically ill patients (eg. a bay, ward, high dependency unit)<br>2.8.2 Areas for separating and managing patients with a suspected or confirmed contagious disease from those without

PANEL 3. The essential diagnosis-specific care for critically ill patients with COVID-19

CLINICAL PROCESSES

1. The Essential Emergency and Critical Care (EECC) clinical processes as specified for all critical illnesses
2. Personal Protective Equipment (PPE) that is appropriate for COVID-19 as part of Infection, Prevention and Control (IPC)
3. Monitoring oxygen saturation using pulse oximetry at least every 6 hours, unless otherwise prescribed
4. Intermittent prone positioning
5. Low molecular weight heparin or other anticoagulant
6. Corticosteroid
7. Antibiotics in patients with suspected bacterial superinfection

HOSPITAL READINESS REQUIREMENTS

Critically ill patients with COVID-19 require the same hospital readiness for EECC as other critically ill patients. For the provision of the essential diagnosis specific care of critically ill patients with COVID-19, the following additional items are required:

CATEGORY	ITEM
3.1 EQUIPMENT	None
3.2 CONSUMMABLES	3.2.1 Facemasks appropriate for COVID-19 (eg. N95)<br>3.2.2 Eye protection or face shields
3.3 DRUGS	3.3.1 Low-molecular weight heparin (eg. enoxaparin or dalteparin) or other anticoagulant<br>3.3.2 Corticosteroid (eg. dexamethasone)
3.4 HUMAN RSOURCE	3.4.1 Health workers with the ability to care for critically ill COVID-19 patients 24hrs/day
3.5 TRAINING	3.5.1 The health workers are trained in essential care of critically ill COVID-19 patients
3.6 ROUTINES	3.6.1 Routines for care of critically ill COVID-19 patients
3.7 GUIDELINES	3.7.1 Guidelines for essential care of critically ill COVID-19 patients
3.8 INFRASTRUCTURE	3.8.1 Areas for separating and managing patients with suspected or confirmed COVID-19 from those without

Appendix 2: The combined in-depth interview guides (Guide 1 – frontline healthcare workers; Guide 2 – administrative healthcare workers)

2a) GUIDE 1: Interview Guides for Frontline Healthcare Workers

PART 1: Consent for recording

Before we begin, please can you confirm that you consent to having this interview recorded in line with the information sheet shared? Everything you say is confidential, the recorded data will not be shared with anyone outside the research team and the data will be anonymized.

PART 2: Introduction and basic information

Thank you for agreeing to participate in this research. Before we begin, I would just like to emphasize again that this is not an inspection or an audit – and no information specific to this hospital will be fed back to your managers. We are just interested in understanding more about the provision of care for critically ill patients in hospitals. So please feel at ease to be as open as possible with your responses.

**Date for interview**	**Day** [ ] [ ] **Month** [ ] [ ] **Year** [ ] [ ] [ ] [ ]
**Interviewer name**	
**County Name**	
**CODE** E.g. HCW_KE_H1_001_01	

GENERAL INFORMATION ABOUT RESPONDENT

**Gender**	1) Male … 01<br>2) Female… 02
**Age**	
**Job Title**	
**Name of hospital**	
**Name of department**	
**Highest educational qualification achieved?**	
**What kind of medical training do you have?**	
**How many years have you been qualified for this post?**	
**How many years have you been working at this hospital?**	

NOTE TO INTERVIEWER: PROVIDE OUR DEFINITION OF CRITICAL ILLNESS AND EXPLAIN THAT THE INTERVIEW QUESTIONS ARE BASED ON THIS DEFINITION.s

*Critical Illness is a state of ill health with vital organ dysfunction, a high risk of imminent death if action is not taken and the potential for reversibility: so, we mean **all the very sick patients who need immediate care** (and not just the patients in intensive care units or other critical care units).*

- Where are critically ill patients (children, pregnant women, medical adults, surgical adults) cared for in your hospital?
  Probes/clarification if they answer "ICU" or something else. Are critically ill patients cared for:
  - *In the OPD?*
  - *In the Emergency Dept?*
  - *In the general wards?*
  - *Anywhere else in the hospital?*
- How are you involved in the care of critically ill patients?
  Probe:
  - *in what capacity?*

- *In which department?*
- *For how long?*

PART 3: SCENARIO-BASED QUESTIONS

I will now ask a series of questions based on patient scenarios. This is not a test, I am just interested in the systems in your hospital and department. Please answer these questions based on what happens in your hospital, not what should happen based on your clinical training or in textbooks.

3.1 TRIAGE AND ADMISSION

NOTE TO INTERVIEWER: YOU MIGHT SKIP THROUGH RAPIDLY IF THE INTERVIEWEE IS NOT VERY FAMILIAR WITH THIS PROCESS.

Assume a new adult patient arrives at your hospital outpatient department/emergency department (as applicable) with symptoms of severe breathing difficulties.

- Can you describe what happens to this patient on arrival at your hospital?
  Probe:
  - *Who is the first person to help or provide care on arrival? Are they medically trained? Are they emergency medicine trained?*
  - *Registration system (paperwork, payments)?*
  - *Are there delays? Why?*
- How would the severity of this patient's condition be assessed to identify if he/she is critically ill? How does this work?
  Probe:
  - *Who does this?*
  - *Are triage categories used (mention which ones they use)/understood?*
  - Do you look for or screen people who might have signs associated with contagious diseases?
  - *Are Vital sign checked? Which ones?*
- Who is in-charge of triage at the outpatient department? How does this change over shifts?
  Probe:
  - *Night shifts versus day shift*
  - *How are your staff communicating with each other in this department? Are there differences in how staff members communicate with each other?*
- What care do you provide for critically ill patients in the OPD/emergency department in your hospital?
  Probe:
  - *If they become unconscious, what do you do? If they get shock or low blood pressure, what do you do? If their airway becomes unclear, what do you do?*
  - *Where you trained on the approach you use?*
  - *Is there a written guideline or protocol that you follow for unconscious patients?*
- *Is oxygen available in the OPD/emergency department?*
  Probe:
  - *In what form? Concentrators? Cylinders? Piped to bedside?*
  - *Is the supply reliable? How often are there shortages (e.g. weekly).*
  - *How many days in a week do you have patients who need oxygen but you aren't able to provide it?*
  - *How do you decide if the oxygen supply is enough?*
  - *If oxygen runs out or components are missing, what process do you use to report/resolve this?*
  - *If not: why not? How do you manage?*
- Where would this patient be admitted and how would they get there?
  Probe:
  - *Clarify process- straight to ward for admission or via resuscitation room/other?*
  - *Do patients have to wait along the way? And why?*
  - *Does the system for handover and communication with the receiving department/unit/ward work? Why not?*
- Overall, what are the biggest challenges you face during triage and admission of critically ill patients in your hospital?

For peer review only

Probe:

- *Reflect on what has already been discussed: equipment, supplies, communication, drugs, guidelines, routines, training, staffing issues?*

3.2 MANAGEMENT, MONITORING AND ESCALATION OF CARE

NOTE TO INTERVIEWER: YOU MIGHT SKIP THROUGH RAPIDLY OR COMPLETELY DEPENDING ON POSITION OF INTERVIEWEE AND HOW FAMILIAR HE/SHE IS WITH THIS PROCESS. USE YOUR JUDGMENT TO ADJUST QUESTIONS IF REQUIRED, BASED ON WHERE THE INTERVIEWEE WORKS.

*Now, consider the ward or unit where you work the most. Assume this **critically ill** patient has been admitted to your ward/unit with a clinical syndrome consistent with a pneumonia*

- Can you briefly describe the patient handover process when a critically ill patient is admitted at your ward/unit?
  Probe:
  - *Who is involved?*
  - *How do staff communicate with each other both in the department and with other departments?*
  - *Does it work? Why not?*

- After admission, how are critically ill patients checked or monitored for deterioration or improvement?
  Probe:
  - *What monitoring is done? How would you know a patient is deteriorating?*
  - *Are vital signs checked? Which ones?*
  - *How often is this monitoring done? By whom?*
  - *How is this documented and communicated to others on duty?*
  - *Where in your department are they monitored?*
  - *Is someone responsible/in- charge? Who?*
  - Are patients consulted or their opinions asked when the team makes a management decision?
- Is medical oxygen always available in your ward/unit for such patients?
  Probe:
  - *In what form? Concentrators? Cylinders? Piped to bedside?*
  - *Is the supply reliable? How often are there shortages (e.g. weekly). Is it available on all wards?*
  - *If not: why not? How do you manage?*

- What care do you provide for critically ill patients in your ward/unit?
  Probe: if they become unconscious, what do you do? If they get shock or low blood pressure, what do you do? If their airway becomes unclear, what do you do? Are there any written guidelines you follow for this? If so do they work well and how?
- What training have you received to monitor and manage such a critically ill patient?
  Probe:
  - *On the wards, are there any guidelines you follow to monitor and manage patients?*
  - *Who makes decisions about how a patient is monitored in your department?*
  - *Are you confident in being able to identify deterioration? Why/why not?*
  - *Are you confident in being able to care for such patients? Why/why not?*
- What options for escalation of care are available to you, if the patient deteriorates?
  Probe:
  - *CPAP*
  - *High-flow nasal oxygen*
  - *Intubation and mechanical ventilation*
  - *Admission to HDU or ICU*
  - *Referral to another hospital (which hospital?)*
  - *Is the capacity sufficient? Why not?*
- Is there a framework or guideline used in your ward or department to decide when to stop certain treatments?" If so is it helpful?
- Are senior staff available to advise/help for management of critically ill patients?

For peer review only

Probe:

  - *Which cadres and types of staff?*
  - *Is there a system for alerting them? Please describe.*
  - *How effective is this system? Is anyone in-charge of checking it is working?*
  - *Differences between night and day shifts?*

- What are the biggest challenges you face when monitoring and managing critically ill patients admitted to your ward/unit?
  Probe: Reflect on what has already been discussed: equipment, supplies, communication, drugs, guidelines, routines, training, staffing issues?

PART 4: GENERAL QUESTIONS

NOTE TO INTERVIEWER: YOU MIGHT NOT HAVE TIME TO DO ALL OF THIS. DO NOT PROBE IF TIME IS VERY LIMITED.

We know that some hospitals have experienced increased cases of patients with respiratory illness in the past 1 year due to COVID-19, and we are interested in how hospitals have managed.

- Did your hospital face any surge in patients with respiratory illness in the past one year due to COVID-19?
- How has care for critically ill patients changed in your facility since the COVID-19 pandemic?
  Probe:
  - *New emergency department or unit?*
  - *New HDU/ICU or increased capacity? Is it in use? Are there any plans to improve its use if its not fully operational? What was the previous capacity?*
  - *Increased number of staff working in critical care, emergency care, anaesthesia? Where have they been placed?*
  - *New equipment or supplies for emergency or critical care? Are they in use? What was you previous equipment capacity on the wards and in OPD/emergency department?*
  - *Increased oxygen capacity? E.g did you get a new oxygen plant? Is it in use?*
  - *Changed processes for identifying and managing patients who need higher levels of care? Has this been beneficial?*
- Have there been changes made to how things are done in this facility or your department in the past (I.e note keeping, handover, handling equipment etc)?
  - If so who started it? How did people receive this change?
  - Why was it successful or not successful?
  -
- Has your hospital received any support (funding/donations in kind/technical support) to strengthen management of the critically ill due to COVID-19? or is any support expected in the coming months?
  Probe:
  - *What support and from whom?*
  - *Do you think it has helped/not helped? Why? (Equipment, drugs, additional staff, supplies, training)*
  - *If no: has this been challenging?*
  - *How did your hospital cope e.g. repurpose staff, longer shifts, borrow equipment from other departments?*
- Compared to pre COVID-19 pandemic, do you think your hospital is more ready to identify, care for and monitor critically ill patients?
  Probe:
  - *If yes/no, how and why?*
- Are there any important issues that still need to be fixed, which you haven't already mentioned?
  Probe:
  - *Equipment, supplies, staff, training, routines/guidelines, communication, systems*
- Finally, if we want to run a program to improve the management of all critically ill patients, what would you recommend that we do?
  Probe:
  - *What additional support would have the most impact/be most helpful?*
  - *How would this improve the care of critically ill patients*

Thank you very much for your time.

For peer review only

2b) GUIDE 2: Interview Guide for Administrative Healthcare Workers- Kenya

PART 1: Consent for recording

Before we begin, please can you confirm that you consent to having this interview recorded in line with the information sheet shared? Everything you say is confidential, the recorded data will not be shared with anyone outside the research team and the data will be anonymized.

PART 2: Introduction and basic information

Thank you for agreeing to participate in this research. Before we begin, I would just like to emphasize again that this is not an inspection or an audit – and no information specific to this hospital will be fed back to your managers. We are just interested in understanding more about the provision of care for critically ill patients in hospitals. So please feel at ease to be as open as possible with your responses.

**Date for interview**	**Day** [ ] [ ] **Month** [ ] [ ] **Year** [ ] [ ] [ ] [ ]
**Interviewer name**	
**County Name**	
**CODE** E.g. HCW_KE_H1_001_01	

GENERAL INFORMATION ABOUT RESPONDENT

**Gender**	3) Male … 01<br>4) Female… 02
**Age**	
**Job Title**	
**Name of hospital**	
**Name of department**	
**Highest educational qualification achieved?**	
**What kind of medical training do you have?**	
**How many years have you been qualified for this post?**	
**How many years have you been working at this hospital?**	

NOTE TO INTERVIEWER: PROVIDE OUR DEFINITION OF CRITICAL ILLNESS AND EXPLAIN THAT THE INTERVIEW QUESTIONS ARE BASED ON THIS DEFINITION.

*Critical Illness is a state of ill health with vital organ dysfunction, a high risk of imminent death if action is not taken and the potential for reversibility: so, we mean **all the very sick patients who need immediate care** (and not just the patients in intensive care units or other critical care units).*

PART 3: SCENARIO-BASED QUESTIONS

I will now ask a series of questions based on patient scenarios. This is not a test, I am just interested in the systems in your hospital and department. Please answer these questions based on what happens in your hospital, not what should happen based on your clinical training or in textbooks.

3.1 TRIAGE AND ADMISSION

NOTE TO INTERVIEWER: YOU MIGHT SKIP THROUGH RAPIDLY IF THE INTERVIEWEE IS NOT VERY FAMILIAR WITH THIS PROCESS.

For peer review only

Assume a new adult patient arrives at your hospital outpatient department/emergency department (as applicable) with symptoms of severe breathing difficulties.

- Can you describe what happens to this patient on arrival at your hospital?
  Probe:
  - *Who is the first person to help or provide care on arrival? Are they medically trained? Are they emergency medicine trained?*
  - *Registration system (paperwork, payments)? How do you decide which payments must be made upfront?*
- Who decides which systems are used to identify the severity of this patient's condition if he/she is critically ill? How does this work?
  Probe:
  - *Are there staff trainings in this department on systems to manage critically unwell patients?*
  - *Who is responsible for training staff on which systems to use?*
  - *Are anaesthetists or critical care staff involved in training staff in your department on critical illness management?*
  - *Who ensures staff have the equipment they need to use these systems?*
- Who is in-charge of triage at the outpatient department? How does this change over shifts?
  Probe:
  - *Night shifts versus day shift*
  - *Who decides how many staff members are needed for each shift?*
- *Is oxygen available in the OPD/emergency department?*
  Probe:
  - *In what form? Concentrators? Cylinders? Piped to bedside?*
  - *Is the supply reliable? How often are there shortages (e.g. weekly).*
  - *Who is responsible for monitoring oxygen supply? Who maintains the oxygen delivery systems?*
- If this patient was admitted to a ward, how would staff communicate between departments?
  Probe:
  - *Who is responsible for setting up and maintaining communication systems?*

3.2 MANAGEMENT, MONITORING AND ESCALATION OF CARE

NOTE TO INTERVIEWER: YOU MIGHT SKIP THROUGH RAPIDLY OR COMPLETELY DEPENDING ON POSITION OF INTERVIEWEE AND HOW FAMILIAR HE/SHE IS WITH THIS PROCESS. USE YOUR JUDGMENT TO ADJUST QUESTIONS IF REQUIRED, BASED ON WHERE THE INTERVIEWEE WORKS.

Now, consider the ward or unit where you work the most. Assume this ***critically ill*** *patient has been admitted to your ward/unit with a clinical syndrome consistent with pneumonia*

- After admission, how are critically ill patients checked or monitored for deterioration or improvement?
  Probe:
  - *What monitoring is done? How would staff know a patient is deteriorating?*
  - *Are vital signs checked? Which?*
  - *How often is this monitoring done? By whom?*
  - *How is this documented and communicated to others on duty?*
  - *Where in your department are they monitored?*
  - *Is someone responsible/in- charge? Who?*
- Is medical oxygen always available in your ward/unit for such patients?
  Probe:
  - *In what form? Concentrators? Cylinders? Piped to bedside?*
  - *Is the supply reliable? How often are there shortages (e.g. weekly). Is it available in all wards?*
  - *Who is responsible for monitoring oxygen supply? Who maintains the oxygen delivery systems?*

For peer review only

- What training have staff received to monitor and manage such a critically ill patient?
  Probe:
  - *When did they receive this training?*
  - *Do they receive refresher sessions? How often?*
  - *Who decides which staff should undergo training?*
- What options for escalation of care are available to you, if the patient deteriorates?
  Probe:
  - *Is there a guideline or SOP staff use to escalate care?*
  - *Who decides which guidelines are used?*
- Is there a framework or guideline used in your ward or department to decide when to stop certain treatments?"

PART 4: GENERAL QUESTIONS

NOTE TO INTERVIEWER: YOU MIGHT NOT HAVE TIME TO DO ALL OF THIS. DO NOT PROBE IF TIME IS VERY LIMITED.

We know that some hospitals have experienced increased cases of patients with respiratory illness in the past 1 year due to COVID-19, and we are interested in how hospitals have managed.

- Did your hospital face any surge in patients with respiratory illness in the past one year due to COVID-19?
- How has care for critically ill patients changed in your facility since the COVID-19 pandemic?
  Probe:
  - *New emergency department or unit?*
  - *New HDU/ICU or increased capacity? Is it in use? Are there any plans to improve its use if its not fully operational? What was the previous capacity?*
  - *Increased number of staff working in critical care, emergency care, anaesthesia? Where have they been placed?*
  - *New equipment or supplies for emergency or critical care? Are they in use? What was you previous equipment capacity on the wards and in OPD/emergency department?*
  - *Increased oxygen capacity? E.g did you get a new oxygen plant? Is it in use?*
  - *Changed processes for identifying and managing patients who need higher levels of care? Has this been beneficial?*
- Have there been changes made to how things are done in this facility or your department in the past (I.e note keeping, handover, handling equipment etc)?
  - If so who started it? How did people receive this change?
  - Why was it successful or not successful?
- Has your hospital received any support (funding/donations in kind/technical support) to strengthen management of the critically ill due to COVID-19? or is any support expected in the coming months?
  Probe:
  - *What support and from whom?*
  - *Do you think it has helped/not helped? Why? (Equipment, drugs, additional staff, supplies, training)*
  - *If no: has this been challenging?*
  - *How did your hospital cope e.g. repurpose staff, longer shifts, borrow equipment from other departments?*
- Finally, if we want to run a program to improve the management of all critically ill patients, what would you recommend that we do?
  Probe:
  - *What additional support would have the most impact/be most helpful?*
  - *How would this improve the care of critically ill patients?*

Thank you very much for your time.

For peer review only

Appendix 3: Process map – Facility 1

Entry and triage

- The hospital receives patients either as walk-ins/referrals
- The triage nurse sorts patients to their respective care points
- Preliminary vital signs are taken by the triage nurse

Registration

- The patient then proceeds to the registration window
- Staff facilitate registration process for emergency cases
- Depending on the queue size, this takes 10-45 minutes

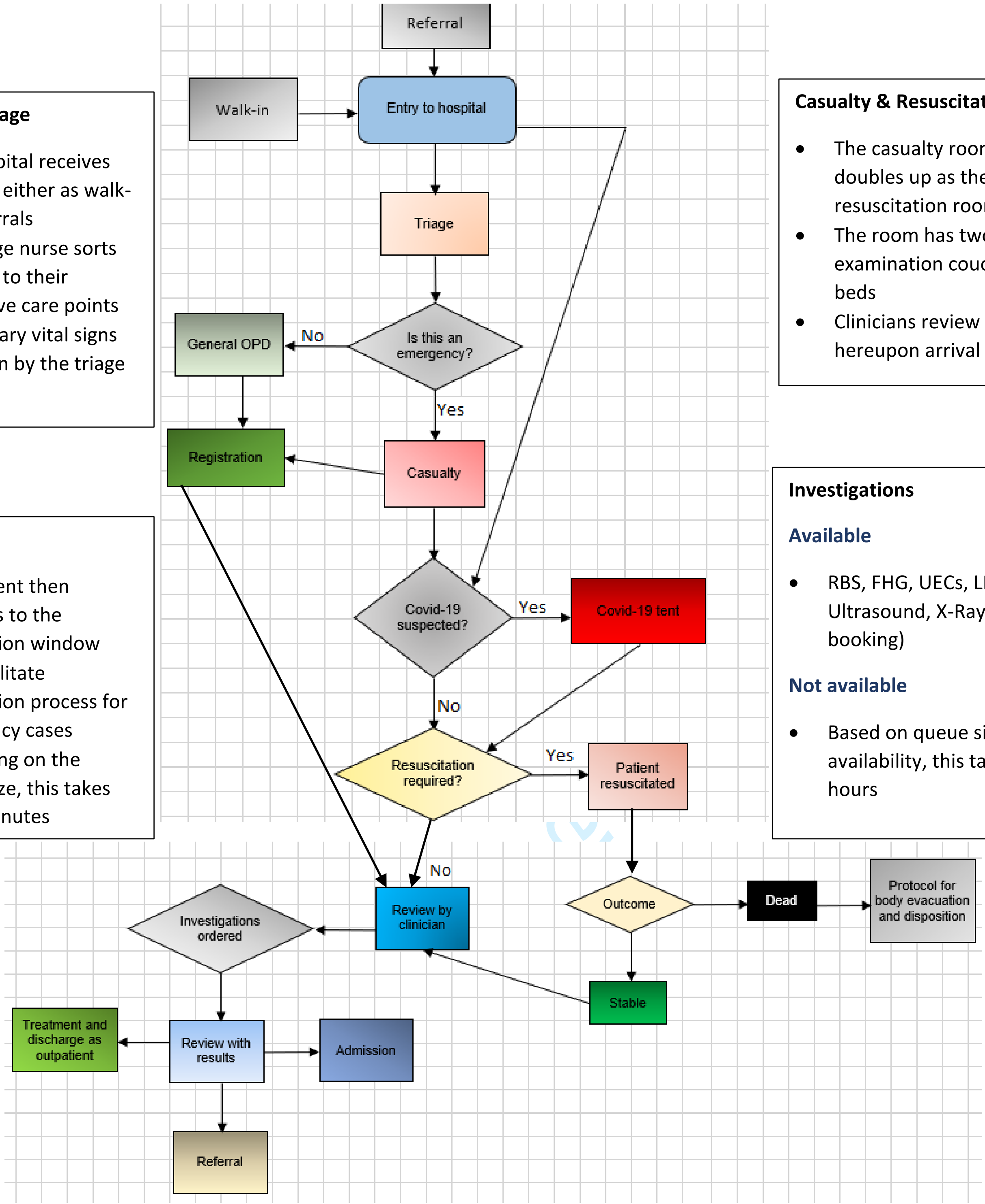

Casualty & Resuscitation

- The casualty room also doubles up as the resuscitation room
- The room has two examination couches and 5 beds
- Clinicians review patients hereupon arrival

Investigations

Available

- RBS, FHG, UECs, LFTs, Ultrasound, X-Ray, MRI (upon booking)

Not available

- Based on queue size and availability, this takes 1-4 hours

Consultation protocol

- Clinical Officer (+/- clinical officer interns) sees patient
- Clinical Officer calls Medical Officer/registrar
- MO/registrar calls consultant

Appendix 4: Process map – Facility 3

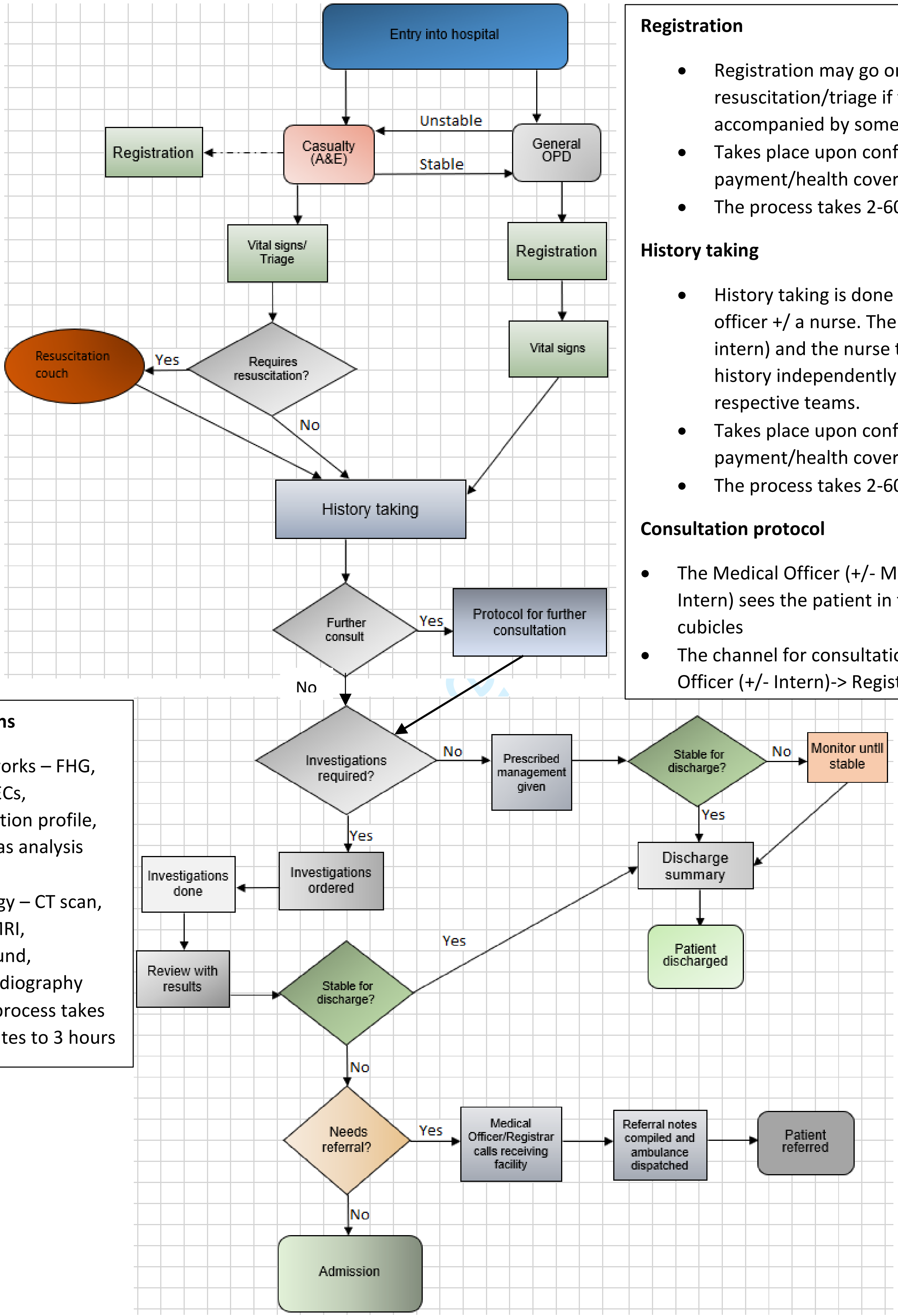

Registration

- Registration may go on during resuscitation/triage if the patient is accompanied by someone
- Takes place upon confirmation of payment/health cover
- The process takes 2-60 minutes

History taking

- History taking is done by a medical officer +/ a nurse. The MO (or MO + MO intern) and the nurse take patient history independently for their respective teams.
- Takes place upon confirmation of payment/health cover
- The process takes 2-60 minutes

Consultation protocol

- The Medical Officer (+/- Medical Officer Intern) sees the patient in the consultation cubicles
- The channel for consultation is Medical Officer (+/- Intern)-> Registrar -> Consultant

Investigations

- Blood works – FHG, LFTs, UECs, Coagulation profile, Blood gas analysis (BGA)
- Radiology – CT scan, X-ray, MRI, Ultrasound, Echocardiography
- Whole process takes 30 minutes to 3 hours

Appendix 5: Patient Journeys

5a) Journey of a patient presenting with severe abdominal pains (later diagnosed with ruptured ectopic pregnancy) at Facility 1.

Date: September 2, 2021

Narrator: OO, Assistant Research Officer – POETIC-COVID project

10:20 am: We have just received an adult female patient – she has been brought in [at the A&E] accompanied by two relatives. She is in obvious distress and reports symptoms of severe progressive abdominal pain. She has been put on a bed – the nurse and a clinician (Clinical Officer) are already with her. I'll keep you posted.

10:24 am: The vitals have been taken: SPO2 is 93% on room air, the heartrate is 90 b/min. Other parameters seem, okay? A thing to note is that this unit, the ER, has five couches, four oxygen ports and there are two bedside monitors that are operational. So, I'll keep you posted.

10:30 am: The nurse is fixing an IV line, together with the Clinical Officer.

10:34 am: The Clinical Officer has written a lab request for a blood test [full hemogram]. The nurse has drawn a blood sample to be taken to the lab. So, the patient is still in bed.

10:40 am: The nurse has repeated the vital signs; BP is 93/50mmHg. The nurse is putting up IV normal saline [500 ml] right now.

10:57 am: Status update: patient still in bed, IV fluid running. No other consultations made.

11:20 am: The first bottle of IV is over, the nurse has put up a second 500ml bottle of normal saline. The relatives have been asked to go make payments for the blood sample to be taken to the lab for processing. They are yet to make the payments.

11:30 am: The second IV is running well. We still await review. Notable is that the unit is favourably staffed, I can count five nurses in the shift – although one of them is the unit in-charge, but the other four are pretty much on the ground – also two Clinical Officers and one Medical Officer.

11:35 am: The relatives have gone to make payments, after which the blood sample will be taken to the lab.

12:04 pm: The relatives are back with the receipt of payment. So, any time now the sample will be taken to the lab.

12:21 pm: The second IV normal saline is still running. Vital signs have been repeated: BP is 98/59mmHg. Also, on assessment, the patient was noted to be pale. We still await the results from the lab.

12:45 pm: The Medical Officer has reviewed the patient and has requested for an abdominopelvic ultrasound to determine what could be the cause of her discomfort and symptoms. The IV is still running. Meanwhile, the relatives have been asked to go make payment for the radiological tests.

1:07 pm: The second IV fluid is still running. It is around the change-over time, new staff are coming in for the afternoon shift. They are sitting together, and a hand-over process is going on - the new staff are being briefed on the patients' conditions – so we hope that things are going to be swift and the patient is going to receive the care soonest possible.

For peer review only

1:14 pm: The patient is being wheeled to the radiology department for the abdominopelvic ultrasound. She's accompanied by her relatives. Payment to be made at the cashier's window next to the department.

1:15 pm: We are at the radiology department: there is a queue, so, the patient will have to wait outside on the wheelchair before she's seen. Payment is being made.

1:45 pm: One relative [patient's sister] has come back to the casualty [A&E]. Apparently, she has been told that the test imaging (abdominal ultrasound) cannot be done until a later time, maybe even tomorrow since the patient is required to have fasted before the radiological test. We are yet to establish if there's anything else that can be done.

1:52 pm: The doctor has instructed the patient's sister to communicate to the radiology department technicians that the test is urgent and that this patient is due for admission into the ward. We are hoping that the patient is going to get the test done soon. The patient is still waiting outside the radiology department on a wheelchair in the company of the other relative.

1:57 pm: The patient has been wheeled back to the casualty department, but the test has not been done because the radiology team want a clear communication from the casualty on the urgency of the test.

2:05 pm: The doctor has indicated [in writing] in the patient's file that the test request is URGENT, and it needs to be done as soon as possible. That communication is being relayed to the radiology through the file that is to be taken there by the patient's relatives. The patient is being wheeled back to the radiology department. We hope that the test is going to be done and the results to be out soon because this patient is supposed to be admitted.

2:15 pm: The patient has been wheeled back again into the A&E from the radiology department. The test has not been done. The radiology team holds that the abdominal ultrasound cannot be done right now because the patient is needed to have fasted so it will have to be done later. The doctor at the A&E has reviewed the initial request and ordered specifically for a pelvic ultrasound. The patient is to be taken for a pelvic ultrasound soon.

2:25 pm: The patient has been wheeled to the radiology department again, with the new request, so we await feedback. We hope that the test is going to be done so she can proceed to the next care point. They are to make payment for the new test and reverse the initial payment.

2:35 pm: Patient is in the waiting bay at the radiology department. She has been instructed to take some water (has a litre of water) to have a full bladder to aid the pelvic ultrasound. The patient's sister has gone to make the payment.

3:03 pm: The patient is still waiting outside. The waiting area seems packed; a lot of patients waiting to be seen. We are just hoping that the patient will be in soon.

3:37 pm: Status update: not much has moved, the patient is still waiting outside.

5:20 pm: Patient still waiting in the waiting bay. She is next in line to be seen, likely in the next ten or less minutes.

5:33 pm: The patient has just been wheeled into the radiology room.

5:50 pm: The patient has just left the radiology room. The results are expected to be out within the next 30 minutes, so they'll have to wait a little longer after she leaves the radiology department.

6:17 pm: Final update for Patient 1. The patient has been wheeled back to the casualty, awaiting review with the radiology results. This marks the end of my follow-up with this patient because of time but from

For peer review only

the look of things the doctor says the patient is to be admitted, although that is after review with results once the results are out.

Follow up call at 8:26 am - Friday September 3: The patient was admitted in the gynaecological ward at night around 10:30pm with a diagnosis of a ruptured ectopic pregnancy and was taken to theatre for urgent surgical intervention [exploratory laparotomy].

5b) An adult male patient brought in unconscious at Facility 2 (Case 2 in the main document)..

Date: September 13, 2021

Narrator: OO, Assistant Research Officer – POETIC-COVID project.

12:06 pm: I am at Facility 2. The patient has just been brought in, carried by two relatives. He looks unconscious. The patient has been put on a chair in the ER. The ER has two rooms; A& B, each with 4 beds.

12:08 pm: The nurse and two EMTs are setting up to take vital signs. The random blood sugar (RBS) has just been taken and is 8.5 mmol/L. The patient is still unresponsive. Meanwhile, the relatives have been asked to go start the registration process.

12:10 pm: The vitals seem okay except for the SPO_2: the BP is 130/75mmHg, HR is 81b/min and SPO_2 is 85% on room air. The ER doesn't have piped oxygen, but we have two huge oxygen tanks with flowmeters attached (no flow splitters). Right now, the two cylinders are both in use. The room has two monitors with one SPO_2 probe attached to one of the monitors. The other SPO_2 probe is in use at the triage area.

12:19 pm: The patient has been taken to the other room, where a space has been created in a bed. The patient is yet to be reviewed; the relatives are not yet back from the registration office.

12:22 pm: The patient has been put onto a bed in the other room, but he's sharing the bed with another patient since the space is full. This room also has 4 beds that are currently all occupied. Two more patients are sitting in chairs with IV drips. This cubicle has one oxygen tank with a capacity of about 50.2L.

12:31 pm: The Medical Officer and two EMTs are with the patient. The EMTs are fixing an IV cannula and drawing a blood sample into two vacutainers.

12:35 pm: The blood samples have been drawn into the two sample containers. The Medical Officer has drawn up a lab request and handed it to the patient's relative to deliver to the lab upon making the payments. The patient is still in bed and awaits further review.

12:55 pm: A 500ml bottle of IV Ringer's Lactate has been set up and is running well. The patient is yet to be reviewed.

1:16 pm: The IV is over and the patient still awaits further review. The patient is still in distress. No further vital signs have been taken. We await the next course of steps. The ER is quite busy, and there's a queue outside of patients waiting to be seen. The two rooms in the ER are full as well, with patients sharing beds. We are just hoping that the patient is going to be reviewed soon.

1:25 pm: The patient has been noticed to be having convulsions. We still haven't had a concrete review because the doctor is quite held up and the patient's details have not been updated.

1:40 pm: The doctor has prescribed an anticonvulsant. The prescription has been handed to a relative to pick up the drug from the pharmacy upon payment. The unit is still very busy, with very sick patients being wheeled in.

2:41 pm: The medication has just been brought and will be administered any time now. The patient is still in bed in status quo; still unresponsive and having convulsions. The results are not yet out.

2:56 pm: The nurse has just given the injection of the anticonvulsant, so, right now we await that to take effect. We hope the patient will be reviewed soon; the lab results are still not out.

3:50 pm: The results are still not out, and the patient still awaits a review. No further vital observations have been taken.

4:15 pm: The patient hasn't been reviewed yet; the results are yet to be received back from the lab. No further vitals have been taken. The patient's condition hasn't improved much, but the convulsions are not as strong as they were before. This is the final update for today because of time.

5c) Journey of a patient who presented with symptoms of severe difficulty in breathing (Covid-19 patient) at Facility 4 (Case 3 in the main document).

Facility: Facility 4

Date: June 9, 2021

Narrator: OO, Assistant Research Officer – POETIC-COVID project.

0830h: Morning. This is OO, the ARO for the POETIC-COVID study. It is about 8:30 am, a patient has just been wheeled into the casualty [ER] with apparent symptoms of severe breathing difficulties. The patient is being wheeled onto a couch in the casualty. The Medical Officer is still not yet in. The doctor [Medical Officer] was here overnight, so he has stepped out to refresh before coming back. Upon coming back, we hope the patient will be reviewed and if at all there are any investigations to be taken the patient will be taken accordingly. That's all for now, we wait for the next steps.

0835h: Update for Patient 2, it is 8:35 am. The vitals have just been taken. It has been noted that the heartrate is a little unstable, ranging as low as from 50s to 60s but at times it's spiking, so not really stable. She's in obvious breathing difficulty [distress]. The SPO2 currently is reading 58% off oxygen. To be noted again is that the unit has one pulse oximeter that is operational. There is another one attached to the monitor but not working. The other patients…there is one other patient who needs constant pulse oximetry, but he has his own…the family came with a personal pulse oximeter, so I think for that he's sorted for now. That is allfor now, I'll keep you posted.

0900h: Update for Patient 2, it's 9 am. The doctor arrived at around 8:57 am. He has reviewed the patient as well as the others. He's suggesting an X-ray, so the relatives have gone to the registration window to open a file for the patient. So, we await that after which the prescribed test [X-ray] …she'll be wheeled in.

0915h: Patient 2, the time is 9:15 am. The patient has just been wheeled in for the X-ray. Being that she isamong the very first patients who reported to this casualty we are hoping that it won't take long, she's going to be attended to almost immediately. So, we hope that she'll be out in the next few minutes.

0937h: The patient has just left the X-ray department, awaiting review with results [results are not out yet]…so, the patient is back in the casualty. I'll keep you posted. It's now 9:37 am, the patient went in at 9:15 am and is already out. So, we'll follow up upon review of the results.

For peer review only

0950h: The patient has been put on oxygen, in bed at the casualty department awaiting the X-ray results. Not really connected to a monitor because the monitors are faulty, but the patient is on oxygen, so from time to time the vitals are being checked. I'll keep you posted.

1015h: The results are out…the X-ray results are out but apparently there has to be a Covid test done. TheRDT [Covid rapid test] is not available in that department so the patient has to be taken away again; a stone-throw away within the hospital for the test. The relatives are still following up with whoever is responsibleon how best that can be done, but otherwise, the patient is still on oxygen, we are awaiting a review of theX-ray results. There is a queue…there's one patient ahead so the Medical Officer is still with another patientbut once he's done, I think the patient will be reviewed with the X-ray results. However, we are still following up with the Covid team for the Covid test. I'll keep you posted.

1043h: Update for Patient 2, 10:43 am. The Covid team came to take samples for the rapid test from the casualty. The samples are being taken, so we await the next few minutes for the results to be out after

which it'll inform the direction of care; whether this patient is going to be admitted in the medical ward orthe Covid isolation ward. I'll keep you posted, that's the update for now.

1127h: Update for Patient 2, it is 11:27am. The test results for Covid-19 are out, she has been confirmed tobe Covid positive. So, the patient has been indicated to be admitted in the Covid isolation ward. Initially they had planned to admit her in the female medical ward, but now that the results are out, she's going tobe taken to the isolation unit. One thing about the isolation unit (I was already there a just a while ago), it has a capacity of six [6]. It has a total of 6 beds. At the moment four [4] of those are occupied, so we are hoping that this patient is going to get space. We are just hopeful that the space avails…we keep on waitingat the casualty. The patient is still on oxygen via face mask, the vitals-the SPO2 is okay. She's saturating on97% on oxygen, but the pulse rate was a little worrisome initially. It was going as low as 52, at one point even at 43 beats per minute but at the moment we cannot say that she's so badly off. She's still on oxygen, pulse rising to above 68. That's the update for now.

1140h: Update for Patient 2. The vitals have been taken again…still the SPO2 is ranging between 90-93% onoxygen. To be noted again is that there is no high-flow oxygen in this facility due to the oxygen supply [strained]. Again, in the casualty there is on oxygen port which is being used to give oxygen to this patient via mask. There's no non-rebreather mask in the department as at now but hopefully the SPO2 will rise, it is promising. Again, this unit is actually very full at this point, remember there is only one medical officer. The unit is full and other patients are actually complaining. Also there's another patient who is having similar issues [as Patient 2] and they [the medical team] were trying to locate a bed space for the patient…there has been a push-and-pull between the Medical Officer here and the destination hospital [FACILITY X], they have been arguing over phone and the Medical Officer seems frustrated here. Looks like he feels his hands are tied and the patients need help and they cannot be able to help but we are hoping that things ease up over the next few minutes.

1254h: Update for Patient 2, the time is 12:54 pm. It has been confirmed that there is bed space for this patient in the Covid isolation ward in this Facility. Her latest SP02 was 94%. So, we are waiting for a stretcherto be brought so that the patient can be wheeled there.

1304h: Update for Patient 2, the time is 1304h. The patient has just been put on the stretcher to be takento the Covid isolation ward…the patient is being admitted in the Covid isolation ward. The patient is to be taken there on portable oxygen in a cylinder. They're setting that up. I'll keep you posted once the patientlands in the Covid isolation ward. They have just left the casualty.

For peer review only

1319h: Update for Patient 2, the time is 1319h. The patient has just been transferred to the Covid isolation unit...patient has been admitted. To be noted is that this patient is currently saturating at 94% on oxygen but on admission [when she landed at the casualty] the patient was actually saturating as low as 17% - thatwas off oxygen. The patient has been maintained in the casualty on oxygen and has been transferred into the Covid isolation unit. As it stands the patient has been admitted into the isolation unit with Covid-19 Pneumonia. I'll halt there for now, that marks the end of my journey with

5d) An adult male patient brought in unconscious at Facility 2.

Facility 2
Date: September 13, 2021
Narrator: OO, Assistant Research Officer – POETIC-COVID project.

11:40 am: I am in the A&E department at Facility 2. The ER has two rooms: A & B, each with 4 beds. At the moment all eight beds are occupied. There's no piped oxygen in the department but I can count three oxygen cylinders with a capacity of 50.2L each. Right now, two of the cylinders are in use. Each cylinder has a flowmeter attached, but no flow splitters. The cylinders serve one patient at a time.

11:45 am: An adult male patient has been brought in, carried by two relatives, apparently unconscious.

11:48 am: The patient is in a chair (all 8 beds are currently occupied), still not responsive. The nurse and an assistant - looks like a trained Patient Attendant or an EMT – are attending to the patient. They are setting up to take the patient's vital signs. The SPO2 probe has just been put. The EMT has fixed an IV line.
The RBS is 3.4 mmol/L. A blood sample has been drawn, to be taken to the lab upon payment by the relatives. I'll keep you posted.

12:15 pm: The patient is still in a chair since the 4 beds in this cubicle are occupied. There is an adjacent cubicle but that as well is currently full. The nurse and an EMT are trying to set up an IV fluid, but they first have to settle the patient in a bed. They are trying to create a bed space by relocating another patient who has stabilized. Just to add, this department has 3 nurses, 1 Medical Officer and 2 Emergency Medical Technicians (EMTs). The EMT assist with wheeling the patients as well as fixing IV lines.

12:21 pm: The patient has been put onto a bed, but he's sharing the bed with another patient since the space is full. The nurse has just put up a 500 ml bottle of 5% IV Dextrose. The patient's relatives have been given the sample to deliver to the lab upon making payment at the laboratory department.

12:40 pm: The IV Dextrose is still running. No further review at this point. The department is quite busyand packed with patients. The results are not yet out from the lab.

12:48 pm: The patient is currently responsive; he is waking up. The IV Dextrose is still running, but still no further review yet.

1:15 pm: The IV fluid (5% Dextrose) is over. The patient is awake and looks stable. We still await review. The lab results are not out yet. The patient has been taken outside to wait on the bench.

1:50 pm: The patient is stable and has been asked to wait outside on the bench in the waiting bay so as tocreate a bed space for another patient. The results are not ready from the lab. The patient will be reviewed once the results are out.

For peer review only

5e) An adult female patient brought in by social worker with symptoms of respiratory distress at Facility 2. Observation was concurrent with 4b above.

Facility: Facility 2
Date: September 13, 2021
Narrator: OO

12:13 pm: This is a new patient – patient 3. The patient is an adult female. She has been brought to the ER in the company of the City County personnel and a Social Worker from the hospital. She is from a street family. She is in obvious distress; generally, she looks unwell and seems to be having respiratory problems. She has been put in a chair since all beds are currently full. We await further steps.

12:31 pm: The patient is still in obvious respiratory distress. She has not been reviewed yet. The patient is alone right now (not in the company of anyone – she is from the street families). She's sitting in a chair but still inobvious distress. I can see she's carrying some medications in her hands, seems to have been seen elsewhere prior to being brought here. She also has an IV cannula already fixed in her arm, apparently fixed outside the hospital. We hope she gets seen soon.

12:41 pm: The patient has just been seen, the vitals have been taken; BP seems to be 110/52 mmHg, HRis 109 b/min and SPO2 is 78% on room air. She's still in a chair, yet to be reviewed further. The beds arestill full, so the patient will have to wait in this chair a little longer.

1:07 pm: The patient has been put on oxygen via face mask. She's still seated on the chair. To note is thatthis patient seems to be having altered mental status with incoherent speech. She's still by herself but occasionally the EMTs are checking up on her, and she's yet to be evaluated in the next round of vital signs. Right now she doesn't seem good: still in severe respiratory distress and altered mental status.

1:15 pm: Patient is still on oxygen, status quo. The nurse has noticed that the patient keeps pulling off the oxygen mask from her face, doesn't want the mask put on her face. The patient still hasn't been reviewed by the doctor. No samples or further vitals have been taken. She's still on oxygen.

1:37 pm: The oxygen has been discontinued for now since the patient is uncooperative. The patient is still in a chair, no improvement. We still await a review, hopefully she'll get seen soon.

1:43 pm: The patient has been noted to have fallen off the chair and is lying on the floor, unresponsive. I have alerted the nurse and she has come accompanied with an EMT. The nurse and the EMT are surrounding the patient, trying to assist. The patient is unresponsive. We hope she's okay.

1:47 pm: The patient has just been confirmed dead. Just to repeat, the patient was brought in by a socialworker and the Nairobi City County team. She has no relatives as of this moment. The morgue is being informed shortly; the body is still on the floor in the ER.

3:00 pm: The body is being prepared for transport to the morgue. This marks the end of my follow-upwith the patient.

For peer review only

Standards for Reporting Qualitative Research (SRQR)*

http://www.equator-network.org/reporting-guidelines/srqr/

Page/line no(s).

Title and abstract

**Title** - Concise description of the nature and topic of the study Identifying the study as qualitative or indicating the approach (e.g., ethnography, grounded theory) or data collection methods (e.g., interview, focus group) is recommended	**Page 1/**line 1-2
**Abstract** - Summary of key elements of the study using the abstract format of the intended publication; typically includes background, purpose, methods, results, and conclusions	**Page 2/**line 1-25

Introduction

**Problem formulation** - Description and significance of the problem/phenomenon studied; review of relevant theory and empirical work; problem statement	**Page 4/**line 1-49
**Purpose or research question** - Purpose of the study and specific objectives or questions	**Page 5/**line 1-8

Methods

**Qualitative approach and research paradigm** - Qualitative approach (e.g., ethnography, grounded theory, case study, phenomenology, narrative research) and guiding theory if appropriate; identifying the research paradigm (e.g., postpositivist, constructivist/ interpretivist) is also recommended; rationale**	**Page 6/**line 3-9
**Researcher characteristics and reflexivity** - Researchers' characteristics that may influence the research, including personal attributes, qualifications/experience, relationship with participants, assumptions, and/or presuppositions; potential or actual interaction between researchers' characteristics and the research questions, approach, methods, results, and/or transferability	**Page 7/**line 13-16
**Context** - Setting/site and salient contextual factors; rationale**	**Page 6/**line 11-20
**Sampling strategy** - How and why research participants, documents, or events were selected; criteria for deciding when no further sampling was necessary (e.g., sampling saturation); rationale**	**Page 6/**line 22-35
**Ethical issues pertaining to human subjects** - Documentation of approval by an appropriate ethics review board and participant consent, or explanation for lack thereof; other confidentiality and data security issues	**Page 6/**line 29-40 and **Page 20/**line 33-37
**Data collection methods** - Types of data collected; details of data collection procedures including (as appropriate) start and stop dates of data collection and analysis, iterative process, triangulation of sources/methods, and modification of procedures in response to evolving study findings; rationale**	**Page 6/**line 42-48 and **Page 7/**line 1-39

For peer review only

For peer review only - http://bmjopen.bmj.com/site/about/guidelines.xhtml

**Data collection instruments and technologies** - Description of instruments (e.g., interview guides, questionnaires) and devices (e.g., audio recorders) used for data collection; if/how the instrument(s) changed over the course of the study	**Page 6/**line 44-48 and **Page 7/**line 29-31
**Units of study** - Number and relevant characteristics of participants, documents, or events included in the study; level of participation (could be reported in results)	**Page 6/**line 44-48
**Data processing** - Methods for processing data prior to and during analysis, including transcription, data entry, data management and security, verification of data integrity, data coding, and anonymization/de-identification of excerpts	**Page 6/**line 46-48 and **Page 7/**line 42-43
**Data analysis** - Process by which inferences, themes, etc., were identified and developed, including the researchers involved in data analysis; usually references a specific paradigm or approach; rationale**	**Page 7/**line 41-50 and **Page 8/**line 1-9
**Techniques to enhance trustworthiness** - Techniques to enhance trustworthiness and credibility of data analysis (e.g., member checking, audit trail, triangulation); rationale**	**Page 7/**line 45-50 and **Page 8/**line 15-22

Results/findings

**Synthesis and interpretation** - Main findings (e.g., interpretations, inferences, and themes); might include development of a theory or model, or integration with prior research or theory	**Page 9/**line 2-8
**Links to empirical data** - Evidence (e.g., quotes, field notes, text excerpts, photographs) to substantiate analytic findings	**From Page 9/**line 10 **to Page 17/**line 6

Discussion

**Integration with prior work, implications, transferability, and contribution(s) to the field -** Short summary of main findings; explanation of how findings and conclusions connect to, support, elaborate on, or challenge conclusions of earlier scholarship; discussion of scope of application/generalizability; identification of unique contribution(s) to scholarship in a discipline or field	**From Page 18/**line 1 **to Page 19/**line 14
**Limitations** - Trustworthiness and limitations of findings	**Page 2/**line 40 to **Page 3/**line 2

Other

**Conflicts of interest** - Potential sources of influence or perceived influence on study conduct and conclusions; how these were managed	**Page 20/**line 19-20
**Funding** - Sources of funding and other support; role of funders in data collection, interpretation, and reporting	**Page 20/**line 22-26

*The authors created the SRQR by searching the literature to identify guidelines, reporting standards, and critical appraisal criteria for qualitative research; reviewing the reference lists of retrieved sources; and contacting experts to gain feedback. The SRQR aims to improve the transparency of all aspects of qualitative research by providing clear standards for reporting qualitative research.

For peer review only - http://bmjopen.bmj.com/site/about/guidelines.xhtml

**The rationale should briefly discuss the justification for choosing that theory, approach, method, or technique rather than other options available, the assumptions and limitations implicit in those choices, and how those choices influence study conclusions and transferability. As appropriate, the rationale for several items might be discussed together.

Reference:

O'Brien BC, Harris IB, Beckman TJ, Reed DA, Cook DA. **Standards for reporting qualitative research: a synthesis of recommendations.** *Academic Medicine*, Vol. 89, No. 9 / Sept 2014 DOI: 10.1097/ACM.0000000000000388

For peer review only

BMJ Open

Third delay in care of critically ill patients: a qualitative investigation of public hospitals in Kenya.

Journal:	*BMJ Open*
Manuscript ID	bmjopen-2023-072341.R2
Article Type:	Original research
Date Submitted by the Author:	23-Nov-2023
Complete List of Authors:	Onyango, Onesmus; KEMRI-Wellcome Trust Research Programme, Health Services Unit<br>Willows, Tamara; University of Oxford, Nuffield Department of Medicine<br>McKnight, Jacob ; University of Oxford, Tropical Medicine; Oxford University<br>Schell, Carl Otto; Karolinska Institutet, Department of Global Public Health; Uppsala University, Department of Global Public Health<br>Baker, Tim; Karolinska Institutet; Ifakara Health Institute<br>Mkumbo, Elibariki; Ifakara Health Institute<br>Maiba, John; Ifakara Health Institute<br>Khalid, Karima; Ifakara Health Institute; Muhimbili University of Health and Allied Sciences, Department of Anaesthesia and Critical care<br>English, Mike; University of Oxford, Nuffield Department of Medicine ; KEMRI-Wellcome Trust Research Programme Nairobi,<br>Oliwa, Jacqueline; KEMRI-Wellcome Trust Research Programme Nairobi, Health Services Unit; University of Nairobi, Department of Paediatrics
**Primary Subject Heading**:	Emergency medicine
Secondary Subject Heading:	Emergency medicine, Evidence based practice, Nursing, Patient-centred medicine, Public health
Keywords:	ACCIDENT & EMERGENCY MEDICINE, COVID-19, Decision Making, Health Services Accessibility, Quality in health care < HEALTH SERVICES ADMINISTRATION & MANAGEMENT, QUALITATIVE RESEARCH

SCHOLARONE™
Manuscripts

I, the Submitting Author has the right to grant and does grant on behalf of all authors of the Work (as defined in the below author licence), an exclusive licence and/or a non-exclusive licence for contributions from authors who are: i) UK Crown employees; ii) where BMJ has agreed a CC-BY licence shall apply, and/or iii) in accordance with the terms applicable for US Federal Government officers or employees acting as part of their official duties; on a worldwide, perpetual, irrevocable, royalty-free basis to BMJ Publishing Group Ltd ("BMJ") its licensees and where the relevant Journal is co-owned by BMJ to the co-owners of the Journal, to publish the Work in this journal and any other BMJ products and to exploit all rights, as set out in our licence.

The Submitting Author accepts and understands that any supply made under these terms is made by BMJ to the Submitting Author unless you are acting as an employee on behalf of your employer or a postgraduate student of an affiliated institution which is paying any applicable article publishing charge ("APC") for Open Access articles. Where the Submitting Author wishes to make the Work available on an Open Access basis (and intends to pay the relevant APC), the terms of reuse of such Open Access shall be governed by a Creative Commons licence – details of these licences and which Creative Commons licence will apply to this Work are set out in our licence referred to above.

Other than as permitted in any relevant BMJ Author's Self Archiving Policies, I confirm this Work has not been accepted for publication elsewhere, is not being considered for publication elsewhere and does not duplicate material already published. I confirm all authors consent to publication of this Work and authorise the granting of this licence.

For peer review only

Third Delay in care of critically ill patients: a qualitative investigation of public hospitals in Kenya.

Onesmus Onyango*[1], Tamara Willows[2], Jacob McKnight[2], Carl Otto Schell[4,5,6], Tim Baker[4,7,8,9], Elibariki Mkumbo[7], John Maiba[7], Karima Khalid[7,10], Mike English[1,2], Jacquie Oliwa[1,3]

1. Health Services Unit, KEMRI-Wellcome Trust Research Programme, Nairobi, Kenya
2. Health Systems Collaborative, Nuffield Department of Medicine, University of Oxford, UK
3. Department of Paediatrics, University of Nairobi, Nairobi, Kenya
4. Department of Global Public Health, Karolinska Institutet, Stockholm, Sweden
5. Centre for Clinical Research Sörmland, Uppsala University, Eskilstuna, Sweden
6. Department of Medicine, Nyköping Hospital, Nyköping, Sweden
7. Ifakara Health Institute, Dar es Salaam, Tanzania
8. Department of Emergency Medicine, Muhimbili University of Health and Allied Sciences, Tanzania
9. Department of Clinical Research, London School of Hygiene and Tropical Medicine, UK
10. Department of Anaesthesia and Intensive Care, Muhimbili University of Health and Allied Sciences, Tanzania

*Correspondence
Onesmus Odongo Onyango
Health Services Unit – KEMRI-Wellcome Trust Research Program, Nairobi, Kenya
OOnyango@kemri-wellcome.org | onesmusodongo@gmail.com

For peer review only

For peer review only - http://bmjopen.bmj.com/site/about/guidelines.xhtml

ABSTRACT

Objectives

Third Delay refers to delays in delivering requisite care to patients after they arrive at a health facility. In low-resource care settings, effective triage and flow of care are difficult to guarantee. In this study, we aimed to identify delays in the delivery of care to critically ill patients and possible ways to address these delays.

Design

This was an exploratory qualitative study using in-depth interviews and patient journeys. The qualitative data were transcribed and aggregated into themes in NVivo 12 Plus using inductive and deductive approaches.

Setting

This study was conducted in four secondary-level public Kenyan hospitals across four counties between March and December 2021. The selected hospitals were part of the Clinical Information Network (CIN).

Participants

Purposive sampling method was used to identify administrative and frontline healthcare providers and patients. We conducted 12 in-depth interviews with 11 healthcare workers and patient journeys of 7 patients. Informed consent was sought from the participants and maintained throughout the study.

Results

We identified a cycle of suboptimal systems for care with adaptive mechanisms that prevent quality care to critically ill patients. We identified suboptimal systems for identification of critical illness, inadequate resources for continuity care, and disruption of the flow of care, as the major causes of delays in identification and the initiation of essential care to critically ill patients. Our study also illuminated the contribution of inflexible bureaucratic non-clinical business-related organizational processes to Third Delay.

Conclusion

Eliminating or reducing delays after patients arrive at the hospital is a time-sensitive measure that could improve the care outcomes of critically ill patients. This is achievable through an essential emergency and critical care package within the hospitals. Our findings can help emphasize the need for standardised effective and reliable care priorities to maintain of care of critically ill patients.

Article summary

This paper highlights a qualitative investigation of delays in identification, monitoring, and treatment of critically ill patients in resource-constrained settings. Critical illness is hereby described as any potentially reversible condition presenting with vital organ dysfunction with a risk of imminent death if care is not provided promptly.

Our study shows the limitations that resource shortages impose on the nature and quality of care administered, and the rigid compensatory systems that emerge. The findings suggest a need for a holistic consideration of workflows with accountability of the whole hospital system, not just focusing on resources, to ensure this performance is maintained across the care pathways.

We conclude that as a standard, the administration of an essential emergency and critical care (EECC) package and sustained transmission of the care urgency is a time-critical and cost-effective measure to improve the clinical outcomes of critically ill patients in low-resource settings.

Strengths and limitations of this study

- This study draws strength from the multiple methods employed, which with triangulation, yielded valuable evidence and support.

For peer review only

- The study was conducted in facilities operating at the same level from four counties across different regions of the country.
- The agreement of various staff from the hospitals with our findings as being a true reflection of their various facilities provided a validation for our study findings.
- However, a wider sample of facilities, healthcare providers, and patients would likely provide more robust evidence to explore our aim.
- Although the use of video for interviewing during COVID has been justified, it is sub-optimal relative to in-person interviews.

For peer review only

For peer review only - http://bmjopen.bmj.com/site/about/guidelines.xhtml

BACKGROUND

Critical illness is defined as a *"state of ill health with vital organ dysfunction, a high risk of imminent death if care is not provided and the potential for reversibility"* [1]. Critical care is herein described as identifying, monitoring, and treating patients with critical illness through the initial and sustained support of vital organ functions. Critically ill patients therefore require prompt, effective care wherever they are in health facilities to reduce the risk of dying. Although the true burden is unknown, the morbidity and mortality from critical illness are thought to be very high and constitute a considerable contribution to the global burden of disease [2-6]. Many of the deaths caused by critical illness are potentially preventable [7, 8] if a prompt, basic, essential package of care (Appendix 1) is provided to these patients [9].

Critical illness gained significant global attention during the COVID-19 pandemic but strategies to improve the management of critically ill patients especially in resource-constrained settings are still in their infancy. With the overwhelming cases of critically ill patients during the COVID-19 pandemic, the Kenyan government – through the national and respective county governments – embarked on setting up COVID-19 isolation units and intensive care units (ICUs) as well as training healthcare workers to improve intensive care capacity [10]. However, these efforts were limited by weak public hospital systems and marked health workforce deficits [11, 12], and facilities continued to see an upsurge of critically ill patients.

Triage is the basis of early identification of critical illness and consequently the first step in the management and survival of critically ill patients in health facilities that attend to large volumes of patients [13-16]. A critically ill patient should ideally be examined immediately on arrival, leading to a decision on the priority level of the patient's condition and their management. For appropriate triage and care delivery, hospitals must have basic treatment materials, skilled personnel with full awareness of the resources and, if necessary, means of transportation for treatment or referrals to be carried out [17-19]. Available evidence suggests that triage, basic emergency care training, better flow in hospitals, and supervision of junior emergency providers reduces care delays and mortality in low-resource settings [20]. In low resource settings, effective triage and flow of care are however difficult to accomplish. Healthcare facilities are frequently characterised by overcrowding, insufficient drugs, limited equipment, and staff shortages [13, 21, 22] as well us a lack of understanding of critical illness [23], contributing directly to delay in essential care. To use the limited human and material resources effectively, different forms of triage are used to classify patients according to the severity of their condition and determine the priority for further treatment [24, 25].

The *Three Delays* model[26, 27] (Figure 1) has been used for decades in obstetrics to illuminate obstacles to the provision of timely and quality care. The First Delay refers to delays in making the decision to seek medical care, mostly due to family and community reasons. Second Delay refers to delays in arriving at a health facility after the decision to seek care has been made due to accessibility challenges. The Third Delay is concerned with delay in delivering care to patients after arrival to a health facility. Reducing all delays improves the clinical outcome of patients both in and outside the hospital, but reducing the Third Delay directly reduces in-hospital morbidity and mortality by adopting a holistic approach to understand the responsibilities of hospital systems.

While the Third Delay model helps us understand and address care gaps in maternal health in resource-constrained settings, the methods and findings of this approach have not been applied substantively to the delivery of care to critically ill patients, especially in low-resource hospitals, outside the obstetric domain. Application of the Third Delay concept through simple strategies such as mapping workflows could therefore be a useful model to identify points of urgent intervention for all patients presenting with critical illness in these settings. Therefore, using this concept, we aimed

For peer review only - http://bmjopen.bmj.com/site/about/guidelines.xhtml

to identify delays in the delivery of care to critically ill patients in Kenyan secondary-level public hospitals.

For peer review only

For peer review only - http://bmjopen.bmj.com/site/about/guidelines.xhtml

METHODS

Study design

This was an exploratory qualitative study design to observe care provided to critically ill patients. The study was part of a larger multi-method project – Provision of Essential Treatment in Critical Illness in the COVID-19 pandemic (POETIC-COVID) – that sought to investigate Essential Emergency and Critical Care (EECC)[9] in Kenyan hospitals during the pandemic. We used in-depth interviews and patient journeys across the selected secondary referral level hospitals and analysed the results in detail using inductive and deductive approaches.

Study setting

Kenya, a lower middle-income country, has a population of approximately 48 million people served by 907 hospitals across 47 counties operating at primary, secondary and tertiary levels [28](Figure 2). The study was conducted in four secondary-level public Kenyan hospitals (Figure 2) across four of the 47 counties in Kenya. The hospitals selected for our study were part of the Clinical Information Network (CIN) [29] and were selected purposively[30] in four counties based on the level of care they provide and their accessibility from Nairobi, where the lead researcher was based, owing to COVID-19 restrictions. All four hospitals included in our study had received funding to scale up critical illness care provision during the COVID-19 pandemic. These hospitals had bed capacities ranging from 185 to 449.

Table 1 below outlines the staffing structure across the departments in the four hospitals. Low staffing numbers were picked across departments in the four facilities. These data were collected in the health facility assessment (HFA) of these hospitals that was a preliminary stage of the POETIC-COVID study.

Table 1: staffing structure across the facilities during the visit (numbers represent one shift).

Key: N= Nurses, CO= Clinical officers, MO= Medical officers. The patient numbers in the A&E department were not included since this was fluctuant across the observation shift.*

* The Medical Officers across all facilities covered more than one department during their shift

** Multiple departments reduced their patient intake due to refurbishment work in Facility. Previous bed capacity for the medical and surgical departments was 32 beds each.

Department	Staff cadre	Staff-to-patient ratios (day shift)			
		Facility 1	Facility 2**	Facility 3	Facility 4
Accident & Emergency	N	4	5	3	2
	CO	1	4	0	2
	MO*	1	2	1	1
Obstetrics	N	10:12	8:37	9:73	2:19
	CO	2:12	2:37	0	0
	MO*	2:12	5:37	2:73	1:19
Paediatric ward	N	4:48	4:25	3:34	1:22
	CO	5:48	4:25	2:34	1:22
	MO*	2:48	3:25	1:34	2:22
Medical wards	N	8:45	4:6	8:35	2:15

For peer review only - http://bmjopen.bmj.com/site/about/guidelines.xhtml

(male + female)	CO	4:45	4:6	0	0
	MO*	2:45	4:6	2:35	1:15
Surgical wards (male + female)	N	8:50	3:8	8:51	3:12
	CO	2:50	3:8	0:51	0:12
	MO*	1:50	4:8	1:51	1:12
ICU	N	3:1		9:5	
	CO	1:1		1:5	
	MO	1:1	-	0	-

Participant selection and recruitment

A purposive sampling method [30] was used to identify administrative staff and frontline healthcare providers from the emergency and out-patient departments, intensive care units (ICUs), maternity units and general wards (paediatric and adult wards). Using staff clinical judgement, the patients were picked from the emergency departments that typically received critically ill patients in these hospitals.

We explained to the eligible participants the purpose and procedure of the study, and that privacy and confidentiality, and their right to opt out at their convenience would be upheld. The number of participants to interview was guided by thematic saturation [31, 32], that is, when no new findings were emerging. Individual written consent was sought from the healthcare workers regarding participation and recording of the interviews and observations. We obtained verbal consent from the patients and/or their relatives about observing the care they received along the pathways.

It was explained that anonymity would be ensured regarding the use of the transcribed interviews, observations, and voice notes in the patient journeys. We explained to the participants that all the audio recordings pertaining to the walkthroughs would be deleted once the recordings were transcribed. Only participants who gave informed consent were recruited in this study.

Data collection

In-depth Interviews (IDIs)

For the four study hospitals, we used a semi-structured interview guide (Appendix 2) to conduct a total of 12 in-depth interviews (IDIs) with 11 frontline healthcare workers – nurses, clinical officers, medical officers – and administrative staff. These interviews were conducted virtually via Microsoft Teams and phone calls owing to the COVID-19 restrictions regarding face-to-face meetings. The interviews, averaging 43 minutes, were audio recorded and saved onto password-protected cloud storage database managed by the KEMRI (Kenya Medical Research Institute)-Wellcome Trust Research Programme (KWTRP). All audio recordings were transcribed verbatim and were deleted after transcription.

Process mapping and Patient journeys

Patient journey mapping is a deliberate patient-oriented approach to better understand barriers, facilitators, experiences, interactions with services and/or outcomes for individuals and/or their caregivers and family members as they enter, navigate, experience, and exit service points in a health system by documenting elements of the journey to produce a visual or descriptive map [33, 34]. The patient journey approach has been applied in patient safety to analyse the care service system and support human centred design [35, 36]. The approach focuses on the sequence of care from the patient's perspective and allows for the examination of discrete patient interactions with providers at each encounter [35]. This can be used to identify gaps that might otherwise go unnoticed.

For peer review only - http://bmjopen.bmj.com/site/about/guidelines.xhtml

The observer – the lead author OO – a research assistant with background training in nursing, conducted the journey mapping during a health facility assessment (HFA) of the four hospitals for the larger POETIC-COVID project. He first identified eight frontline healthcare workers (two per hospital) working in the emergency departments and through informal conversations using scenario-based questions, mapped the journey of a hypothetical critically ill patient when they arrived in the hospitals form the healthcare workers' perspective. OO drew the process maps as described by the health workers in illustrative flow diagrams (Appendix 3). The healthcare workers were asked to estimate the time taken at each step along the journey.

With the help of the staff at the emergency and outpatient departments, the observer then purposively selected and followed 7 critically ill patients as determined by their signs and symptoms from the time of arrival at the Outpatient/Emergency department to the point of admission into the wards or referral out to another facility. The observer noted the care provided and periods of inactivity against the time. Using a Dictaphone, the observer made real-time onsite audio commentary of the steps and activities against the time performed and this data was used to write out each patient journey [37, 38].

To explore Third Delay issues, we assessed the time taken in identification and administration of appropriate care for the selected patients. We used time to triage, time to diagnosis and time to essential care as proxies for determining Third Delay. *Time to triage* here referred to the time from patient's first contact with a healthcare worker (clinical officer, nurse, medical officer, or intern) to the time of departure to the next appropriate care point. Between this time and the time of an objective diagnosis was termed *time to diagnosis*. The time from when an objective diagnosis was made to the administration of appropriate care or intervention was defined as *time to essential care*.

Data analysis

The audio recordings and notes were transcribed verbatim in English then deidentified and imported into NVIVO® 12 Plus to generate nodes and to enable thematic analysis [39-41] using inductive coding and a code book was developed. Four of the authors – OO, TW, EM, and JM – participated in coding transcripts. Separately at first, we used inductive coding to determine fundamental problems in the care critically ill patients currently. Next, we compared the themes with the Delphi that defined EECC backed with clinical input from the team to identify more EECC-appropriate information. Subsequently, we developed a new code book reflecting both the development of the grounded themes and the EECC themes. Transcripts were then recoded using our new framework.
We then abstracted data relevant to the domains in the Third Delay. These were statements speaking to equipment, space, infrastructure, administrative processes, staffing, skills, triage, and diagnostics. The IDI findings were used to build on the observations during the actual patient journeys. We initially read the patient journeys to provide context and further depth in IDIs. We used the narrative form of the patient journey to further develop and test our analytical framework for the IDIs on the provision of care in critical illness.

Patient and public involvement statement

The study design was informed by contemporary experiences of patients in Kenyan public hospitals, especially in light of the COVID-19 pandemic. Throughout the recruitment and conduct of the study, informed consent from patients and/or their relatives was maintained.
We held three feedback meetings with the hospitals after completion of data collection where we shared our findings and checked if our interpretation of the data made sense to hospital staff. Two of these (Facility 1 and Facility 2) were held in-person at the respective facilities, while one (Facility 3) was held virtually via Microsoft Teams. Facility 4 was unable to take part due to their COVID-19 workload. The audiences consisted of healthcare staff from various departments including accident &

For peer review only - http://bmjopen.bmj.com/site/about/guidelines.xhtml

For peer review only

emergency, outpatient, surgery, paediatrics, medical ward, laboratory, radiology, nutrition, and hospital administration. The teams confirmed that our findings were a fair representation of their respective hospitals.

For peer review only

For peer review only - http://bmjopen.bmj.com/site/about/guidelines.xhtml

RESULTS

Themes

The following are findings from the 12 in-depth interviews with the 11 healthcare workers and 4 patient journeys. The combined actual patient journeys are separately attached as Appendix 4. The main findings in this study were combined into three themes: **suboptimal systems for identification of critical illness** (subthemes – informal triage systems (*time to triage*) and delays in early recognition of condition (*time to diagnosis*)), **inadequate resources for initiation and continuity of essential care** (subthemes – resources and infrastructure contribution to delay and shortage of a skilled health workforce), and **disruption of the flow of care** (subthemes – inflexible administrative processes (registration and payments) and inefficient communication systems). These findings are summarised in the theoretical model we developed in this study (Figure 3).

Table 2: A sample patient journey from the perspective of healthcare workers at Hospital 1 (this is a hypothetical patient who ends up needing CT scan)

Step	Activity	Minimum time (minutes)	Maximum time (minutes)
1	Patient arrives in the casualty department		
2	Patient is received by the triage nurse	1	10
3	Preliminary vital signs are taken by the nurse	1	2
4	Patient waits in the queue at the registration window	1	30
5	Confirmation of patient health cover/ payment of registration fee	2	5
6	Patient registration	5	10
7	Waiting in the queue for vital signs checking	1	30
8	Vital signs repeated and recorded by nurse	2	5
9	History taking by nurse	5	20
10	Waiting in the queue for review by clinician	5	45
11	History taking by clinician	5	15
12	Examination by clinician	5	15
13	Investigations (CT scan) ordered	2	2
14	Transport outside facility to seek the required investigation	15	60
15	Waiting in queue for investigation	30	120
16	Waiting for interpretation of results	15	60
17	Travel back for review with CT Scan results	15	60
18	Waiting in queue for review with results	15	45
19	Doctor's review with results	10	45
20	Admission by nurse: nurse calls receiving ward for bed space	5	30
21	Waiting for availability of bed space	5	15
22	Nurse transports patient to destination ward	5	25

Consultation protocol
- Clinical officer (+/- clinical officer interns) sees patient
- Clinical officer calls Medical Officer (MO)/Registrar
- MO/Registrar calls consultant

Investigations
- Available tests: RBS, FHG, UECs, LFTs, Ultrasound, X-Ray, MRI (upon booking)
- Not available: CT-Scan, OGD

Referral protocol
- Through respective specialties;
  - Consultant reviews patient, decides referral
  - Unit manager notified
  - MO/ Registrar calls destination hospital with patient details
  - Ambulance set up and patient referred

Table 2 above outlines the steps from a healthcare worker's perspective of the journey of a hypothetical patient presenting to the Accident & Emergency department, typically as they navigate

For peer review only - http://bmjopen.bmj.com/site/about/guidelines.xhtml

the various care points across the hospital departments of one of the study hospitals. The minimum and maximum time are estimates from the healthcare worker's point of view.

Suboptimal systems for identification of critical illness

Context: *A County Referral Hospital. The A&E (ER) unit has five nurses on the shift – although one of them is the unit manager or 'in-charge' and, as such has duties other than frontline care. The unit also has two Clinical Officers and one Medical Officer. The Emergency Room (ER) has five couches, four oxygen ports, and there are two bedside monitors that are operational.*

An adult female patient arrives at **1020h**, accompanied by two relatives, one of whom is her sister. **The nurse takes a brief history and identifies symptoms of severe abdominal pains**. **Four minutes after the patients' arrival, the nurse takes vital signs** and the patient's SPO_2 is noted to be 93% on room air, and she has a slightly elevated heart rate of 90 beats per minute.

Twenty minutes later, the nurse takes her blood pressure measurement, and it is noted to be **93/50** mmHg. The patient is also found to be clinically pale. The nurse sets up an intravenous infusion of normal saline. At this point, the **clinician has ordered a laboratory request** for a full hemogram, but the sample is not delivered to the laboratory until the patient's relatives **make payments for the laboratory investigations – at 1204h, nearly two hours after she arrived**.

Over two hours after the initial vital signs, the Medical Officer reviews the patient and orders an abdominopelvic ultrasound. The **relative joins the queue at the payment window to make payments** for the investigation. The patient, in a wheelchair, then joins the queue at the radiology department.

At 1345h, a staff member at the radiology department **sends the patient's relative back to the A&E to communicate that the test cannot be done at the moment** because the patient is supposed to have fasted. The back-and-forth between these two departments continues until **1415h** when the doctor at the A&E orders specifically for a pelvic ultrasound. This is now four hours after the patient arrived.

Afterward, the patient is wheeled back to the radiology department and the **relatives queue up at the payment window to update payments for the new test**. The patient remains in the queue until 1733h when she is wheeled into the ultrasonography room. A diagnosis of **ruptured ectopic pregnancy** was made, and the patient was admitted **12 hours later**, at 2230h, for an 'urgent' surgical intervention: an **exploratory laparotomy**.

Informal triage systems (time to triage)

Overall, all four hospitals had a designated area for triage of patients as they arrive at the Casualty (Accident & Emergency) and Outpatient departments and processes to appraise a patient's vital signs – temperature, blood pressure, pulse rate, and oxygen saturation levels – and general status within twenty minutes of arrival (Table 2, Box 1, Box 2) at the Emergency departments. However, there was a consistent lack of routines and systems for identification and continuous patient monitoring after the initial evaluation.

> *"Okay for that one [triage] it's not really clear. It is not clear; there is a no systematic triaging." –* ***HCW3 (Clinical Officer – Facility 1).***

At Facility 1, for example, upon entry into the facility, the patients were directed into the tent where they waited to be seen – mostly on a first-come-first-served basis. The tent was located away from the general outpatient and emergency departments and was set up at the advent of COVID-19 to

For peer review only - http://bmjopen.bmj.com/site/about/guidelines.xhtml

Box 1: Case 1 – The journey of an adult female patient presenting with progressive abdominal pains at Facility 1

screen patients. The tent was reported to be congested with no specific guidelines governing the categorization of these patients in the tent.

> *"[…] there is delayed care because […] you know you have to remain in the triage until you're investigated for COVID at times there are no swabs, so you'll remain there. You see, the lab is far so even when they get their samples they have to go to the lab, back again, take care of other patients along the way you find that the patients can stay outside the tent for even twelve hours so there is delayed care." –* ***HCW3 (Clinical Officer – Facility 1).***

While the hospitals had allocated physical space – room or cubicle – in the emergency and outpatient departments for triage, time to triage was markedly increased and effective triage practice was mostly postponed or forfeited altogether. Triage was largely informal, and the flow of patients was dependent on the availability and number of the staff and resources, as facilities adopted routines that worked most conveniently for these understaffed departments. The stretched capacities in space and staff-to-patient ratios further compounded the effects of ineffective triage.

> *"Yes. Then they issue of congestion because we have outpatient, which is there, we have the emergency department, we have the…they are congested in one place…"* ***HCW6 (Clinical Officer – Facility 4).***

> *"Ok in the triage system, sometimes you find that when we the staffs, there is shortage of staff. Like say you are alone for example like in the paediatric casualty; our acute room is a bit far from where we do triage so when you're handling a patient in the acute room and there is nobody at the triage so you might miss out identification of a critically ill patient in the queue so such scenario, we might end up losing a patient while still on the queue." –* ***HCW1 (Nurse In-charge, Paediatric Department – Facility 2).***

With the overwhelming numbers of critically ill patients presenting in the emergency departments in the hospitals, one hospital activated a functional formal triage system, adopting the South African Triage System (SATS) in the Emergency Department, using colour-coding – red, orange, yellow and green – to classify patients upon arrival.

> *"Initially we didn't have a proper triage system, but we brought it up that we needed a proper triage system and for the past I think three or four days we've been having a proper triaging system, we have like the triage sheet where you see and then we have the list of patients who are emergency patients who patients can wait and like that." –* ***HCW5 (Medical Officer, A&E department – Facility 3).***

> *"So, we are using the South Africa triage system whereby we have at least started coding the patients. We are using the red, orange, green and yellow. So, when a*

For peer review only - http://bmjopen.bmj.com/site/about/guidelines.xhtml

patient comes, we are using the triage chart, which is at the triage area…so we follow that triage, we fill everything and any patient who appears to be red there is…we tag the patient then we take him or her to the emergency area." – ***HCW9 (Nurse In-charge, A&E department – Facility 3).***

Delays in early recognition of condition (time to diagnosis)

Although the hypothetical journey map (Table 2) placed time to diagnosis within 35 minutes, findings from our actual patient journeys found that for tests available in the hospitals, it took 3-12 hours to make a clinical diagnosis after triage and initial evaluation. In-between the time the tests were ordered and when the results came back, the management of the patient was mainly symptomatic, with no system for continuous evaluation of patients' conditions (Box 1, Box 2, Box 3).

Besides being limited in physical space, the diagnostic departments were noted to be lacking variety of tests, occasioning profound delays and often necessitating patient referrals out of the hospitals to source the services. These can be seen in the hypothetical journey map, Case 1 & 2, and in the following quotes from IDIs.

"We only have X-ray, and ultrasound. Yeah, so we don't have a CT scan yet…and MRI we don't have. If we want to do an MRI or a CT scan we have to send outside." – ***HCW1 (Nurse In-charge, Paediatric Department – Facility 2)***.

"Then the other thing is the laboratory. Our laboratory lacks many things; like there is no culture and sensitivity, those are things that miss in our lab. So, if we can have a bigger lab with many areas of assessment, it can be very much okay." – ***HCW10 (Nursing Services Manager – Facility 1)***.

With most hospitals having a centralised payment point, processing the laboratory and radiological tests ordered took considerable amounts of time. The delays were occasioned by long, inflexible registration and payment processes and long queues in these departments.

"…there could be some delays depending with…maybe the patient needs a CT scan, you find the relative they don't have the money to do the CT scan or any other investigation which is not done with the hospital, so those are some of the challenges we get." – ***HCW1 (Nurse In-charge, Paediatric Department – Facility 2)***.

For peer review only - http://bmjopen.bmj.com/site/about/guidelines.xhtml

Inadequate resources for initiation and continuity of essential care

Resources and infrastructure contribution to delay

Context: *The unit has ED has 8 beds, all occupied at the time the patient is brought in. this department has 3 nurses, 1 Medical Officer, and 2 Emergency Medical Technicians (EMTs). The unit is full of patients: some are seated on chairs, others waiting outside on benches. The department lacks piped oxygen but has three oxygen cylinders with flowmeters attached (no flow splitters). The three cylinders are all in use at the time the patient is brought in.*

Four minutes after arrival, at 1210h, the nurse takes vital observations, and the SPO2 is **85%** on room air. At 1222h, the patient is placed on a bed, **but he's sharing the bed with another patient** since the space is full. The patient awaits review. No intervention since the initial vital observations at 1210h. Ten minutes later, the Medical Officer instructs EMTs to fix an IV line and draw blood samples for laboratory investigations.
At 1235h, the Medical Officer writes a laboratory request and hands the blood samples to the patient's **relatives to deliver to the laboratory upon making payment for the tests.** Twenty minutes later, a nurse sets up a 500ml bottle of IV Ringer's Lactate. The patient is yet to be reviewed.
At 1325h, the **patient has been noticed to be having convulsions**. We still haven't had a concrete review because the doctor is quite held up and the patient's details have not been updated. Fifteen minutes later, the Medical Officer prescribes an anticonvulsant. The **prescription is handed to a relative to pick up the drug** from the pharmacy **upon payment**. The unit is still very busy, with very sick patients being wheeled in. The **medication arrives one hour later**, at 1441h, and is administered six minutes later. All this while the patient has not yet been reviewed and is still in bed in status quo; still unresponsive and having convulsions. The results from the lab are not yet out.
At **1615h**, the results are yet to be received back from the lab. No further vitals have been taken. The patient's condition hasn't improved much, but the convulsions have subsided for the moment. The patient is yet to be reviewed.
The observer stopped the observation at 16:30h as his shift ended.

Box 2: Case 2 - The journey of an adult male patient brought in unconscious at Facility 2.

Although the vital observations were done within 20 minutes of patients' arrival at the emergency departments, subsequent monitoring was delayed or not done altogether. The reasons cited for this included limited physical infrastructure and shortage and/or failure of equipment. With the surge of patients needing constant monitoring during the COVID-19 pandemic, this proved to be a major challenge across the four facilities as illustrated in the contextual information of the patient journeys (Box 1, Box 2, Box 3) and the following IDI quotes.

> *"Like we have only one monitor and we encounter many patients, and you see you cannot put this one [patient] on a monitor then take it from them to connect to another patient. We have one monitor. The BP machines are also limited; they are like two. The equipment is there but we cannot say it's enough." –* ***HCW2 (Nurse – Facility 1).***

The few equipment available were sometimes broken down and often shared between the many patients, and occasionally between departments. This further impacted negatively on patient monitoring and care and slowed down interdepartmental or interfacility transfers.

> *"...but sometimes you find the SPO_2 machine is not working, sometimes we don't have thermometers so we might miss out the degree of fever, so things like that. Sometimes*

For peer review only - http://bmjopen.bmj.com/site/about/guidelines.xhtml

they break down or they're stolen. Like the glucometer – you might travel with the patient but now if you can't check on the blood sugar level this patient might be in hypo[hypoglycaemic], but you'll fail to note." – ***HCW9 (Nurse In-charge, A&E department – Facility 3).***

Lack of essential commodities and consumables in the destination departments resulted in slow movement of patients from the outpatient and emergency departments, further slowing down the identification and management of incoming critically ill patients.

"I've seen only one point in Medical Ward. Oxygen is a challenge in the wards; most of the wards. And that is why you find patients of COVID stay here [Emergency Department] longer because they [wards] don't have piped oxygen like we, we have. They have to carry a cylinder. And right now, there is no oxygen." – ***HCW9 (Nurse In-charge, A&E department – Facility 3).***

As this was in the heart of the COVID-19 pandemic, in some cases, adaptive measures like referral and out-of-pocket purchase of these commodities were used to override the shortages and delays occasioned by unavailability of space and oxygen.

"Like recently, the last three four days we didn't have oxygen. So, you tell the patient to make an option where he or she can go to, she can be treated, either Facility 88 or Facility 99 [private hospitals], because we cannot handle them. In real sense, this is a government institution, whereby we are supposed to handle those COVID patients when they come." ***– HCW8 (Nurse, A&E department – Facility 4).***

Shortage of a skilled health workforce

Across the hospitals, human resource shortage was a major hinderance to implementation of care interventions, and the available few staff could not allocate adequate focus to the critically ill patients in these departments.

"The other thing I would require staff who are very well trained. We would require protocols and all staff need to be trained about these protocols. That if a patient presents with this symptom, what next? You know, people don't just walk around without you know, knowing what to do." – ***HCW7 (Medical Officer– Facility 4).***

In addition, the facilities lacked an elaborate training system for staff on emergency and critical care, and since most of the staff train on the job, it was difficult to keep track of staff readiness to provide care to the critically ill patients.

For peer review only - http://bmjopen.bmj.com/site/about/guidelines.xhtml

"And also, many of us, you see we have not been trained all of us about the BLS and ACLS, those ones. Not all of them have been trained; so, if we can get any update training and motivation, that is, it can be very good. Because once you have knowledge, the skills and the technique, those are the three key things you'll need for production." – ***HCW10 (Nursing Services Manager – Facility 1)****.*

In addition, training and retention of skill-specific workforce was picked as a challenge across the hospitals. Constant staff rotations compounded this skill dilution as the trained staff were moved to other departments without replacement of the skillset.

"They keep on changing, rotations…You were in this department, next month you're taken to another department. So, you find that you can be trained in handling emergencies and…you're taken to another department so whoever is replacing you has not been trained so things like that." – ***HCW1 (Nurse In-charge, Paediatric Department – Facility 2).***

Sometimes the strained staffing capacities led to coping measures such as rationing of care even seriously ill patients, in order to spread out the care to as many patients as possible.

"In the wards there is also shortage, so they just check in general, and they if they deteriorate there is resuscitation and they make it, sometimes they don't make it" ***– HCW2 (Nurse – Facility 1).***

For peer review only

Disruption of the flow of care

Inflexible administrative processes (registration and payments)

Context: *The Emergency Room (ER) and the general outpatient are housed under one roof. The unit has one nurse and one Medical Officer (MO) and is full of patients waiting to be seen. The nurse reported at 7:30am and will oversee this unit until 12:30pm when another nurse will come. The medical officer is also covering theatre in case of any surgical emergencies. The ER has three [3] beds/couches for patients, and at the moment, all three beds are occupied. There are also a few nursing students assisting in the care of these patients. Also, two Clinical Officers; one here in the emergency room, the other one covering the outpatient section. The unit has one functional port for piped oxygen which is currently in use. The ER is full. There's a COVID-19 isolation ward but currently that is full as well. There's no departmental phone.*

An elderly male patient – a referral on follow-up for COVID-19 pneumonia – arrives at Facility 4 at **0857h** with symptoms of severe breathing difficulty, although the SPO_2 levels were above 95% on arrival. The patient is kept in the Emergency Department, awaiting allocation of space in the COVID-19 isolation ward at the Facility.

At **1217h**, the Medical Officer reviews the patient and noted the patient to be desaturating; SPO_2 as low as **84%. There's only one oxygen port at the ER, and at the moment it's in use by another patient**. The **MO calls another facility**, Facility X (a regional referral facility), since the isolation unit at Facility 4 is full. **Facility X responds with a promise to call back once a space avails.**

At **1252h** the SPO_2 levels are noted to be below **80%** and the patient is put on oxygen via face mask. The space at Facility X has not been confirmed yet. **There's a back-and-forth between the medical officer and Facility X until 1543h** when the space in the isolation unit at Facility X is confirmed, and the ambulance is set up to ferry the patient.

Box 3: Case 3 – The journey of an adult male patient brought in with symptoms of severe difficulty in breathing at Facility 4

The hospitals lacked systems to override payments for tests, investigations, and essential services for critically ill patients, leading to delays in receipt of adequate and appropriate treatment by patients. Payment for registration, laboratory investigations, and radiological tests were a barrier to quality of care and smooth flow of patients between care points. Prescribed tests and investigations were not processed until the patients, or their relatives paid for these services and in between, care was often withheld while the payments were sorted out.

> *"The issue that we have is this issue of funds. A patient may come to you presenting with hypoglycaemia and you don't know…it's an altered mental status. You really don't know, because the…the relatives have to pay for the RBS [random blood sugar]. You understand?"* – ***HCW7 (Medical Officer – Facility 4).***

Staff in some facilities felt that it would help if there were systems to hold off payments for basic tests until later when the results are out, and the patient has been attended to.

> *"I would require that we have a very clear path with the lab so that we don't require patients to pay out for things like RBS, you know. Essential tests like BS [blood smear] for P. falciparum. So that in that clear path patients can always pay for these things later once, once they get well or whether they make it, or they don't make it"* – ***HCW7 (Medical Officer– Facility 4).***

For peer review only - http://bmjopen.bmj.com/site/about/guidelines.xhtml

Some facilities reported having a waiver process, through the office of the hospital Social Worker, if a patient is unable to pay for a test or investigation. However, even in the facilities that had waiver systems for patients who cannot afford a service, the processes were often very slow and unreliable in the event of critical illness.

> *"So, the challenge is money…money, and you see the patient cannot be waived, nowadays, you have to pay cash. And when you go to the social worker, what he'll tell you is that he or she can waive to a certain point – not beyond maybe I don't know, 2000 [Kenyan shillings], not beyond 3000. That is now the challenge."* – ***HCW9 (Nurse In-charge, A&E department – Facility 3).***

Inefficient communication systems

We identified ineffective communication systems as a major limitation to reducing the *time to essential care* of critically ill patients. Lack of direct interdepartmental communication lines was picked across all four hospitals. Most departments lacked a functional departmental telephone, and communication between departments was largely through the health workers' personal mobile phones (Box 3).

> *"In fact, I was forgetting. That is a very big problem. We don't have communication equipment. It used to be there but they all broke down so right now we use our phones […] It's very difficult because you have to call personal phones. Somebody can even ignore."* – ***HCW2 (Nurse – Facility 1).***

In the four facilities, communication generally relied on the office of the Covering Nurse even in emergencies. This office serves as a call centre for the hospital to provide liaison between departments and to link the hospital with other facilities for inbound and outgoing referrals. In every shift, there's a designated nurse who takes up the role, with a designated facility phone. The covering nurse receives and relays information between the relevant personnel or departments, including the administration when needed. With absence of processes to maintain care in-between, the protocol for escalation of care was also described as long and time-consuming, especially during night shifts and weekends.

> *"The nurse now has to look for the nurse covering and tell the nurse covering that we have a patient, and this patient is to be reviewed by a medical officer. Then the nurse covering calls the medical officer, at the same time the nurse covering calls the driver… At times it takes one hour, two hours, three hours. And sometimes the medical officer is within the hospital yes but doing an operation. So, it means the patients have to wait until the operation is over…That is what happens in the night. In…over the weekend, again there is that delay."* – ***HCW8 (Nurse, A&E department – Facility 4).***

For peer review only - http://bmjopen.bmj.com/site/about/guidelines.xhtml

When probed about the possibility of a better and more prompt communication system, some of the facilities reported that it would be a welcome upgrade, although budgetary constraints would cripple the implementation and sustainability of such a system.

> *"…Maybe if that one [communication system] can be put in place will be able to help our patients."* – ***HCW1 (Nurse In-charge, Paediatric Department – Facility 2).***

In some cases, the faulty communication hardware meant that the staff had to walk to other departments to relay the information in person when planning on transferring patients to the wards from the emergency department. This is not only time-consuming, but also takes away valuable manpower from the already understaffed units.

> *"And it's…it's also difficult because of shortage every time I keep on walking in the ward, "I'm bringing a patient! I'm bringing a patient!" You know, it's also cumbersome."* – ***HCW9 (Nurse In-charge, A&E department – Facility 3).***

In general, *time to essential care* was between 4-12 hours. In Case 1, for example, a follow-up call the following day revealed that the patient was admitted past 10:30 pm and prepared for an emergency surgical intervention, although the patient had arrived at the facility more than 12 hours prior.

For peer review only - http://bmjopen.bmj.com/site/about/guidelines.xhtml

DISCUSSION

We qualitatively examined the patient monitoring and evaluation processes and the limiting factors in the journey of critically ill patients to identify delays in the delivery of care when seeking care in Kenyan secondary-level public hospitals. We found that while the hospitals had allocated physical space in the emergency and outpatient departments for triage, *time to triage* was markedly increased and effective triage practice was mostly postponed or forfeited altogether. Our study also identified inadequate resources, ineffective communication systems, and inflexible administrative processes – bureaucratic non-clinical and business-related organizational procedures such as payments and registration processes – as the major limitations to reducing the *time to diagnosis* and *time to essential care* to critically ill patients within low-resource hospital settings.

Effective management of critically ill patients, i.e., initiation and maintenance of essential care, requires quality care sustained over hours or days including periods spent in emergency departments, during transfers, during waits in diagnostic departments for investigations and sometimes prolonged periods spent on the wards [42]. This quality of continuous care is often reliant on frontline health cadres, especially nurses [43]. However, shortage of skilled healthcare workforce is evidently an ongoing challenge across public hospitals. This is not only with regard to staff-to-patient ratios, but also adequacy of knowledge and skills to implement essential and timely care. This has been attributed to low rates of staff employment and high staff turnovers in addition to discordance in training of frontline staff. The effect of this chronic shortage is development and normalisation of adaptive responses such as rationing of care, as the healthcare workers focus on clearing the overwhelming queues with the available resources in these resource-limited hospital settings [13, 19, 21, 22].

Initial and subsequent evaluation and review of critically ill patients is limited by the chronic shortage of material resources and equipment such as blood pressure machines, thermometers, pulse-oximeters, and also by infrastructural limitations such as physical space and seating for examination of patients upon arrival to the study hospitals. These inadequacies have an immediate and direct effect on the prompt care of patients– as seen in the lengthy delays in triage, diagnosis, and definitive management – but they also have normalised coping mechanisms that further constrain the capacity of the hospitals to improve. All these culminate into a marked Third Delay for patients seeking care in these hospitals. In the case of critically ill patients, the delays could be detrimental to their clinical outcome and lead to preventable mortality. Measures such as a package of interventions focussing on adequate priority infrastructure and medical supplies, prioritizing steps such as triage and stabilization of critically ill patients, sufficiently-trained health care providers, evidence-based care, and referral capacity to support transfers to higher-level care [9, 22] are therefore key in addressing Third Delay in hospitals in resource-limited settings.

Evidence suggests that patient-specific information, particularly in the emergency departments, is an essential component of the patient pathway, the patient-physician relationship, nursing practice, and team working [44]. However, the process of obtaining and availing required patient details should not impede the initiation and/or flow of care especially in critical illness. While it is vital to account for all the services offered to patients in the facilities, there should be straightforward systems in place to ensure that the payments, or lack thereof, do not interfere with the care delivered to critically ill patients and that care is not withheld when the patient is not able to raise the amounts required, or when the payments are yet to be processed. For critically unwell patients, open and functional interdepartmental linkages and communication systems are vital owing to the time-critical nature of their conditions, and the fact that they often cannot advocate for themselves. If the turn-around-time is to be reduced, it is therefore essential to eliminate these administrative stumbling blocks, to reduce the congestion in the emergency care pathways, and capitalize on the cost-effectiveness of the basic emergency care interventions [45-48].

For peer review only - http://bmjopen.bmj.com/site/about/guidelines.xhtml

In chaos, the risks are profound. COVID-19 was pivotal to illuminating areas for improvement in ill-prepared hospital systems globally. In summary, the findings from our study speak to a series of dysfunctional systems for care with adaptive mechanisms that are unideal for the care of critically ill patients. Implementing a guiding principle that the most time-critical, life-saving, and feasible care is the priority when resources are scarce might be a helpful starting point in improving quality of care and outcomes in hospitals. Such a policy, as articulated in the recent national strategic plan for implementing EECC in Tanzania [49] could be useful for the change needed to address defective triage, improper initial and continued evaluation of critically ill patients, strained resource capacities, and administrative bottlenecks that all impede the flow of care for critically ill patients while seeking care in these secondary-level public hospitals. We believe that examining critical illness care through a Third-delay lens would allow us to develop better targeted interventions that improve the likelihood of receiving essential and timely critical illness care services. Figure 3 summarises our concept for the causes of Third Delay in care of critically ill patients.

Study limitations and strengths

Our study took place during the COVID-19 pandemic and access was limited due to movement restrictions. The research was therefore conducted in only four hospitals and was limited to 12 interviews and 7 actual patient walkthrough journeys. A wider sample of facilities, healthcare providers, and patients would likely provide more robust evidence to explore our aims and support our conclusions. Again, owing to the pandemic, there may have been significant, temporal differences in hospital operations that were not identified as such by the respondents and this may have influenced the management of critical patients in ways that were not recorded here. We therefore advocate for follow-up work. The patient journeys method, as we practiced it, is also novel and though very revealing in this study, deserves further refinement. Additionally, although the use of video for interviewing during COVID has been justified [50], it is sub-optimal relative to in-person interviews.

However, this study draws strength from the multiple methods employed, which with triangulation, yielded valuable evidence and support. In addition, the study was conducted in facilities operating at the same level from four counties across different regions of the country, which further fortifies the evidence. Lastly, the agreement of various staff from the hospitals – including administrative staff and unit managers – with our findings as being a true reflection of their various facilities provided a validation for our study findings.

CONCLUSION

Public hospitals in Kenya have long-standing ineffective intra and inter-facility systems that are unreliable in the care of critically ill patients when they present in hospitals. Care of critically ill patients – identification, monitoring and decision making – requires prompt systems to identify and communicate the urgency across multiple care points. Eliminating Third Delay using a sustained package of timely essential care is therefore an important time-critical and cost-effective measure to improve the care outcomes of critically ill patients in resource-constrained settings. Findings from our study can help emphasize the need for standardised effective and reliable care priorities to maintain of care of critically ill patients within hospitals.

For peer review only - http://bmjopen.bmj.com/site/about/guidelines.xhtml

ACKNOWLEDGEMENTS

We are grateful to Prof. Claudia Hanson, Senior Lecturer, Karolinska Institutet, Sweden, Professor, London School of Hygiene & Tropical Medicine, UK and Dr Roy Nobhojit, Surgeon and Researcher, BARC Hospital, HBNI University, Mumbai, India, for their invaluable mentorship that helped shape the concept and direction of this study.

We wish to thank the POETIC-COVID team and the entire EECC network for the immense support and teamwork throughout the study period and beyond. We would also like to acknowledge the contribution of the Ministry of Health – Kenya, the participating hospitals and their staff in the process of this study.

Contributorship statement

OO conceptualized and designed the study, acquired and analysed the data, and developed the first draft of the manuscript with assistance from TW, JO, and JMcK. TB, ME, and COS contributed to the conceptualization and design of the study. TW, EM, JM, KK, and OO analysed the data together. JO also contributed to acquisition of data and was the Scientific Lead for the larger POETIC-COVID study. All the authors interpreted the findings, critically revised the manuscript, and approved the final version.

Conflict of interest statement

The authors in this study all declare no conflicts of interest.

Funding Statement

This study was funded through the POETIC-COVID project by a grant from Wellcome Trust [221571/Z/20/Z] as part of the *Innovation in Low and Middle-income Countries* Flagship. ME is supported by a Wellcome Senior Fellowship (#207522) with further support for authors from a core grant awarded to the KEMRI-Wellcome Trust Research Programme (#092654).

Data sharing statement

Additional anonymised transcripts of patient journeys, process maps, and in-depth interviews are attached separately as supplemental material submitted alongside this manuscript. The raw data supporting this article will be availed by the authors whenever necessary.

Ethics approval statement

This study was part of a larger study approved by the KEMRI Scientific and Ethical Review Committee (SERU Number 4085) and London School of Hygiene and Tropical Medicine (REF 22 866). Institutional approvals were also obtained at hospital level and/or their relevant County liaison for all four hospitals.

For peer review only

REFERENCES

1. Kayambankadzanja, R.K., et al., *Towards definitions of critical illness and critical care using concept analysis.* medRxiv, 2022.
2. Adhikari, N.K., et al., *Critical care and the global burden of critical illness in adults.* The Lancet, 2010. **376**(9749): p. 1339-1346.
3. Bell, M.B., et al., *Prevalence and sensitivity of MET-criteria in a Scandinavian University Hospital.* Resuscitation, 2006. **70**(1): p. 66-73.
4. Dart, P.J., et al., *An evaluation of inpatient morbidity and critical care provision in Zambia.* Anaesthesia, 2017. **72**(2): p. 172-180.
5. Kruisselbrink, R., et al., *Modified early warning score (MEWS) identifies critical illness among ward patients in a resource restricted setting in Kampala, Uganda: a prospective observational study.* PloS one, 2016. **11**(3): p. e0151408.
6. Razzak, J., M.F. Usmani, and Z.A. Bhutta, *Global, regional and national burden of emergency medical diseases using specific emergency disease indicators: analysis of the 2015 global burden of disease study.* BMJ global health, 2019. **4**(2): p. e000733.
7. Molyneux, E., S. Ahmad, and A. Robertson, *Improved triage and emergency care for children reduces inpatient mortality in a resource-constrained setting.* Bulletin of the World Health Organization, 2006. **84**: p. 314-319.
8. Roy, N., et al., *Learning from 2523 trauma deaths in India-opportunities to prevent in-hospital deaths.* BMC health services research, 2017. **17**(1): p. 1-8.
9. Schell, C.O., et al., *Essential Emergency and Critical Care: a consensus among global clinical experts.* BMJ Glob Health, 2021. **6**(9).
10. Maina, M., et al., *COVID-19: an opportunity to improve infection prevention and control in LMICs.* The Lancet Global Health, 2020. **8**(10): p. e1261.
11. Zhao, Y., J. McKnight, and M. English. *Low-income countries' bids for World Bank funding raise serious concerns about their coronavirus strategies*. 2020 [cited 2022 7th January]; Available from: https://theconversation.com/low-income-countries-bids-for-world-bank-funding-raise-serious-concerns-about-their-coronavirus-strategies-138628.
12. Kenya, M.o.H., *Kenya_2019-Novel-Coronavirus-Contingency-Readiness-and-Early-Response-Plan-February-April-2020.pdf*, M.o. Health, Editor. 2020: Accessed online Jan 2022.
13. Knight, H.E., A. Self, and S.H. Kennedy, *Why are women dying when they reach hospital on time? A systematic review of the 'third delay'.* PLoS One, 2013. **8**(5): p. e63846.
14. Sorensen, B.L., et al., *Advanced life support in obstetrics (ALSO) and post-partum hemorrhage: a prospective intervention study in Tanzania.* Acta Obstet Gynecol Scand, 2011. **90**(6): p. 609-14.
15. Vora, N., N. Maitra, and P. Pandya, *Use of the Maternal Foetal Triage Index-Addressing the Third Delay in Obstetrics: An Observational Study.* Authorea Preprints, 2021.
16. Gupta, S., et al., *Third delay in traumatic brain injury: time to management as a predictor of mortality.* Journal of neurosurgery, 2019. **132**(1): p. 289-295.
17. Cavallaro, F.L. and T.J. Marchant, *Responsiveness of emergency obstetric care systems in low-and middle-income countries: A critical review of the "third delay".* Acta obstetricia et gynecologica Scandinavica, 2013. **92**(5): p. 496-507.
18. Gabrysch, S. and O.M. Campbell, *Still too far to walk: literature review of the determinants of delivery service use.* BMC pregnancy and childbirth, 2009. **9**(1): p. 1-18.
19. Downar, J., et al., *A framework for critical care triage during a major surge in critical illness.* Can J Anaesth, 2022. **69**(6): p. 774-781.
20. Hsia, R.Y., et al., *Prehospital and emergency care: updates from the disease control priorities, version 3.* World journal of surgery, 2015. **39**(9): p. 2161-2167.

For peer review only - http://bmjopen.bmj.com/site/about/guidelines.xhtml

21. Floyd, L., et al., *The introduction of a midwife-led obstetric triage system into a regional referral hospital in Ghana.* Midwifery, 2018. **61**: p. 45-52.
22. Morof, D., et al., *Addressing the third delay in Saving Mothers, Giving Life districts in Uganda and Zambia: ensuring adequate and appropriate facility-based maternal and perinatal health care.* Global Health: Science and Practice, 2019. **7**(Supplement 1): p. S85-S103.
23. Mkumbo, E., et al., *Same label, different patients: Health-workers' understanding of the label 'critical illness'.* Frontiers in health services, 2023. **3**: p. 1105078.
24. Sasser, S., A. Kellermann, and J. Lormand, *Prehospital trauma care systems. Geneva: World Health Organization; 2005.* Arh Hig Rada Toksikol, 2013. **64**: p. 567-579.
25. Surgeons, C.o.T.o.t.A.C.o., *Resources for optimal care of the injured patient 2006.* Chicago, IL: American College of Surgeons, 2006.
26. Barnes-Josiah, D., C. Myntti, and A. Augustin, *The "three delays" as a framework for examining maternal mortality in Haiti.* Social science & medicine, 1998. **46**(8): p. 981-993.
27. Thaddeus, S. and D. Maine, *Too far to walk: maternal mortality in context.* Social science & medicine, 1994. **38**(8): p. 1091-1110.
28. MOH, *Kenya Harmonized Health Facility Assessment (KHFA) 2018/2019*. 2019, Nairobi MOH.
29. Tuti, T., et al., *Improving documentation of clinical care within a clinical information network: an essential initial step in efforts to understand and improve care in Kenyan hospitals.* BMJ Global Health, 2016. **1**(1): p. e000028.
30. Palinkas, L.A., et al., *Purposeful sampling for qualitative data collection and analysis in mixed method implementation research.* Administration and policy in mental health and mental health services research, 2015. **42**: p. 533-544.
31. Lowe, A., et al., *Quantifying thematic saturation in qualitative data analysis.* Field methods, 2018. **30**(3): p. 191-207.
32. Pope, C., S. Ziebland, and N. Mays, *Qualitative research in health care: Analysing qualitative data.* BMJ: British Medical Journal, 2000. **320**(7227): p. 114.
33. Davies, E.L., et al., *Reporting and conducting patient journey mapping research in healthcare: A scoping review.* Journal of Advanced Nursing, 2023. **79**(1): p. 83-100.
34. Sijm-Eeken, M., J. Zheng, and L. Peute, *Towards a lean process for patient journey mapping–a case study in a large academic setting*, in *Digital Personalized Health and Medicine*. 2020, IOS Press. p. 1071-1075.
35. Simonse, L., A. Albayrak, and S. Starre, *Patient journey method for integrated service design.* Design for Health, 2019. **3**(1).
36. Trbovich, P. and C. Vincent, *From incident reporting to the analysis of the patient journey.* 2019.
37. Holden, R.J. and P. Carayon, *SEIPS 101 and seven simple SEIPS tools.* BMJ Quality & Safety, 2021. **30**(11): p. 901-910.
38. Simonse, L., A. Albayrak, and S. Starre, *Patient journey method for integrated service design.* Design for Health, 2019. **3**(1): p. 82-97.
39. Guest G and MacQueen N, *Introduction to Thematic Analysis*, in *Applied Thematic Analysis*. 2012, SAGE: Los Angeles.
40. Fereday, J. and E. Muir-Cochrane, *Demonstrating rigor using thematic analysis: A hybrid approach of inductive and deductive coding and theme development.* International journal of qualitative methods, 2008. **5**(1): p. 80-92.
41. Patton, M.Q., *Qualitative research & evaluation methods, Thousand Oaks Sage Publications.* New York, New Delhi, London, 2002.
42. English, M., et al., *Hospital care for critical illness in low-resource settings: lessons learned during the COVID-19 pandemic.* BMJ Global Health, 2023. **8**(11): p. e013407.
43. Gathara, D., et al., *Missed nursing care in newborn units: a cross-sectional direct observational study.* BMJ quality & safety, 2020. **29**(1): p. 19-30.

For peer review only - http://bmjopen.bmj.com/site/about/guidelines.xhtml

44. Cohen, E.L., et al., *When Patients Are Impatient: The Communication Strategies Utilized by Emergency Department Employees to Manage Patients Frustrated by Wait Times.* Health Communication, 2013. **28**(3): p. 275-285.
45. in *Disease Control Priorities in Developing Countries*, D.T. Jamison, et al., Editors. 2006, The International Bank for Reconstruction and Development / The World Bank

Oxford University Press

Copyright © 2006, The International Bank for Reconstruction and Development/The World Bank Group.: Washington (DC)

New York.
46. Jayaraman, S., et al., *First things first: effectiveness and scalability of a basic prehospital trauma care program for lay first-responders in Kampala, Uganda.* PLoS One, 2009. **4**(9): p. e6955.
47. Shanahan, T., et al., *Aligning emergency care with global health priorities.* International Journal of Emergency Medicine, 2018. **11**(1): p. 1-4.
48. Shah, H.A., et al., *Cost Effectiveness of Strategies for Caring for Critically Ill Patients with COVID-19 in Tanzania.* PharmacoEconomics-open, 2023: p. 1-16.
49. MOH, T., *National Strategic Plan on Essential Emergency and Critical Care Services (2023-2026). 2022*. 2022.
50. Sutherland, A.E., J. Stickland, and B. Wee, *Can video consultations replace face-to-face interviews? Palliative medicine and the Covid-19 pandemic: rapid review.* BMJ supportive & palliative care, 2020. **10**(3): p. 271-275.

For peer review only

For peer review only - http://bmjopen.bmj.com/site/about/guidelines.xhtml

Figure Legends

1. Figure 1: The framework of the Third Delay model (adapted from Gabrysch & Campbell, and Cavallaro & Marchant). The loop represented by the green connectors illustrates the continuous re-assessment and monitoring of acutely ill patients needed to identify critical illness. The loop represented by the orange dotted connectors represents a second referral to a higher-level facility. Any complications that cannot be managed at the first facility should be promptly diagnosed and referred within the shortest time possible to eliminate **second emergency delay**.

2. Figure 2: Organization of the Kenyan public health system

3. Figure 3: An empirical concept of the causes of Third Delay in the care of patients presenting with clinical illness.

For peer review only

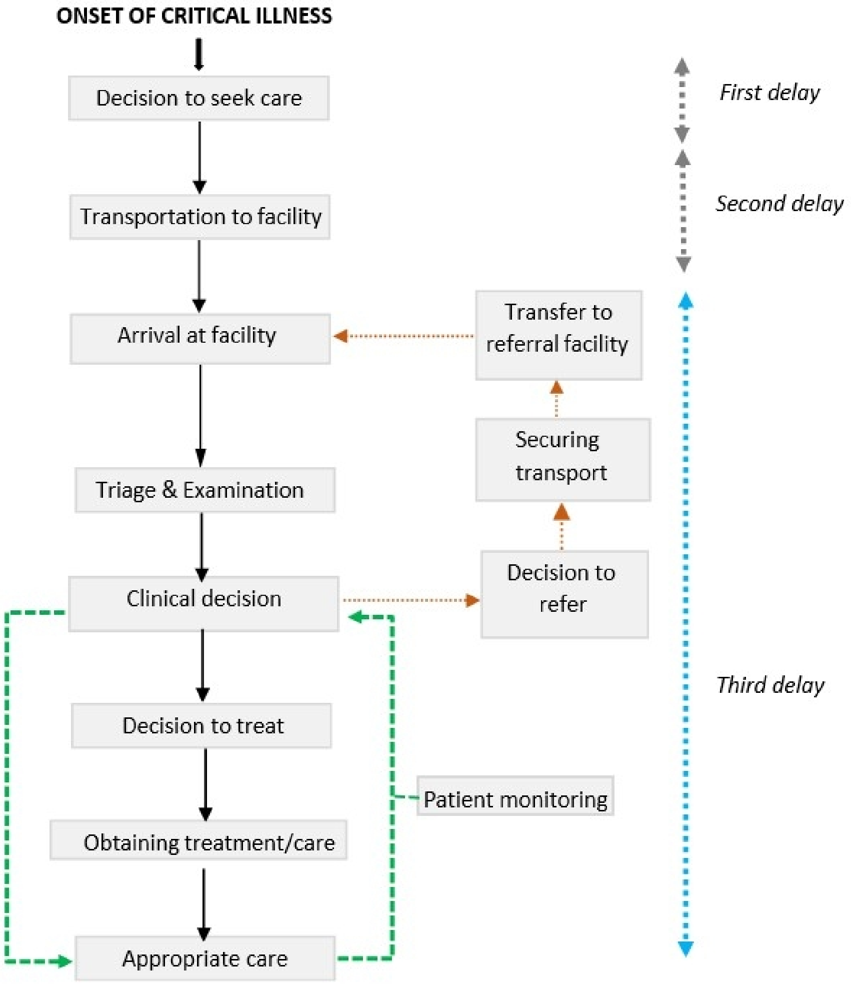

The framework of the Third Delay model (adapted from Gabrysch & Campbell, and Cavallaro & Marchant). The loop represented by the green connectors illustrates the continuous re-assessment and monitoring of acutely ill patients needed to identify critical illness. The loop represented by the orange dotted connectors illustrates a second referral to a higher-level facility. Any complications that cannot be managed at the first facility should be promptly diagnosed and referred within the shortest time possible to eliminate second emergency delay.

146x155mm (120 x 120 DPI)

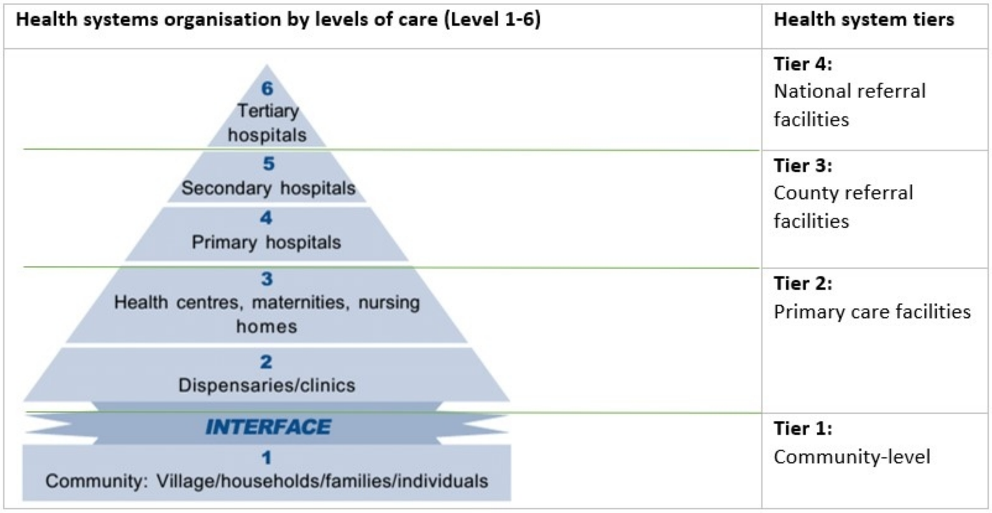

Organization of the Kenyan public health system by levels of care

170x87mm (120 x 120 DPI)

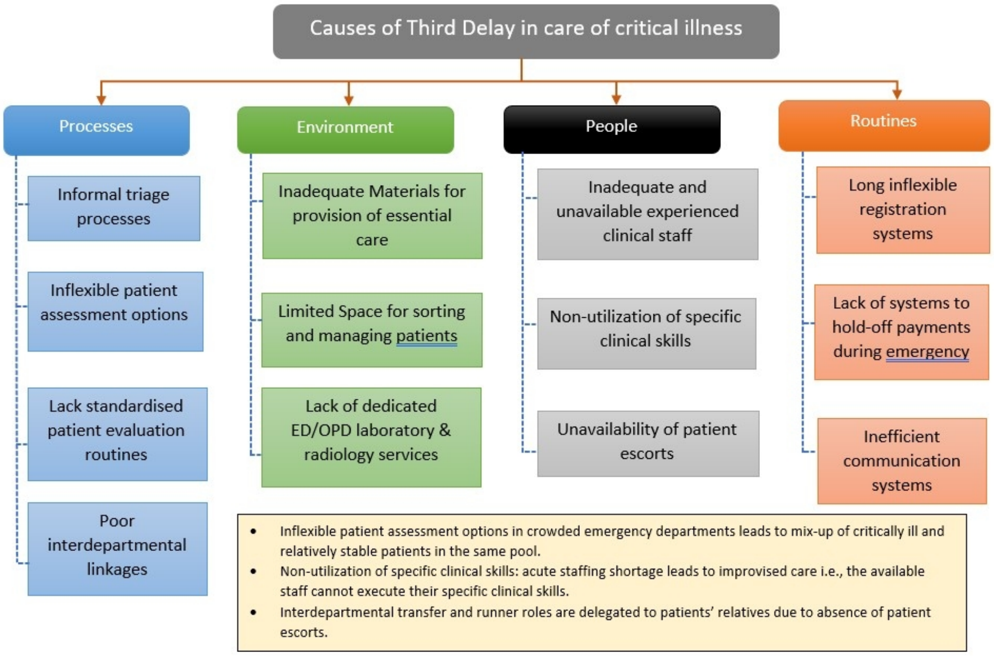

A theoretical concept of the causes of Third Delay in the care of patients presenting with clinical illness

251x165mm (96 x 96 DPI)

APPENDICES

Appendix 1: EECC checklists

PANEL 2. The Hospital Readiness Requirements for Essential Emergency and Critical Care

IDENTIFICATION OF CRITICAL ILLNESS

The following items are required for a hospital to be ready for the identification of critically ill patients:

CATEGORY	ITEM
1.1. EQUIPMENT	1.1.1 Clock with second hand<br>1.1.2 Pulse oximeter & probe<br>1.1.3 Blood pressure measuring equipment (eg. sphygmomanometer with a stethoscope)<br>1.1.4 Blood pressure cuffs of different paediatric and adult sizes<br>1.1.5 Light source (lamp or flashlight)<br>1.1.6 Thermometer
1.2 CONSUMABLES	1.2.1 Soap or hand disinfectant<br>1.2.2 Examination gloves
1.3 DRUGS	None
1.4 HUMAN RESOURCES	1.4.1 Health workers with the ability to identify critical illness 24h/day
1.5 TRAINING	1.5.1 The health workers are trained in the identification of critical illness
1.6 ROUTINES	1.6.1 Routines for the identification of critical illness
1.7 GUIDELINES	1.7.1 Guidelines for the identification of critical illness
1.8 INFRASTRUCTURE	1.8.1 Designated triage area (area for the identification of critical illness) in the Out-Patient Department or Emergency Unit (area of the hospital where patients arrive)<br>1.8.2 Running water

only

PANEL 2 continued ...

CARE OF CRITICAL ILLNESS

The following items are required for a hospital to be ready to provide the care of critically ill patients:

CATEGORY	ITEM
2.1 EQUIPMENT	2.1.1 Suction machine (electric or manual)<br>2.1.3 Oxygen supply 24h/day (cylinder, concentrator (with electricity supply) or piped oxygen)<br>2.1.4 Flow meter (if using cylinder or piped oxygen)<br>2.1.5 Leak-free connectors from oxygen source to tubing<br>2.1.6 Bag Valve Mask (resuscitator) – neonatal, paediatric and adult sizes<br>2.1.7 Sharps disposal container<br>2.1.8 External heat source
2.2 CONSUMABLES	2.2.1 Suction catheters of paediatric and adult sizes<br>2.2.2 Guedel airways of paediatric and adult sizes<br>2.2.3 Pillows<br>2.2.4 Oxygen tubing<br>2.2.5 Oxygen nasal prongs<br>2.2.6 Oxygen face masks of paediatric and adult sizes<br>2.2.7 Oxygen face masks with reservoir bags of paediatric and adult sizes<br>2.2.8 Masks for Bag Valve Mask (resuscitator) – neonatal, paediatric and adult sizes<br>2.2.9 Compression bandages<br>2.2.10 Plasters or tape<br>2.2.11 Gauze<br>2.2.12 Intravenous cannulas of paediatric and adult sizes<br>2.2.13 Intravenous giving sets<br>2.2.14 Skin disinfectant for cannulation<br>2.2.15 Syringes<br>2.2.16 Nutrition<br>2.2.17 Nasogastric tubes<br>2.2.18 Lubricant for nasogastric tube insertion<br>2.2.19 Intramuscular needles<br>2.2.20 Intraosseous cannulas of different sizes<br>2.2.21 Blankets<br>2.2.22 Facemasks for Infection Prevention and Control<br>2.2.23 Aprons or gowns<br>2.2.24 Charts/notes for documentation<br>2.2.25 Pens
2.3 DRUGS	2.3.1 Oral rehydration solution<br>2.3.2 Intravenous crystalloid fluids (eg. normal saline or Ringer's Lactate)<br>2.3.3 Intravenous dextrose fluid (eg. 5%, 10% or 50%)<br>2.3.4 Oxytocin<br>2.3.5 Adrenaline<br>2.3.6 Appropriate antibiotics<br>2.3.7 Diazepam<br>2.3.8 Magnesium sulphate<br>2.3.9 Paracetamol<br>2.3.10 Local anaesthetic (eg. 2% lignocaine) (eg. for intraosseous cannulation)
2.4 HUMAN RESOURCES	2.4.1 Health workers with the ability to care for critically ill patients 24hrs/day<br>2.4.2 Senior health worker who can be called to assist with the care of critically ill patients 24hrs/day

PANEL 2 continued ...

2.5 TRAINING	2.5.1 The health workers are trained in the care of critically ill patients
2.6 ROUTINES	2.6.1 Routines for managing critically ill patients<br>2.6.2 Routine for the provision of EECC without taking into account patients' ability to pay<br>2.6.3 Routines for who and how to call to seek senior help 24hrs/day, 7 days/week<br>2.6.4 Routines for integrating EECC with other care including the definitive care of the underlying condition (eg. use of condition-specific guidelines)
2.7 GUIDELINES	2.7.1 Guidelines for the essential care of critically ill patients
2.8 INFRASTRUCTURE	2.8.1 Designated space for the care of critically ill patients (eg. a bay, ward, high dependency unit)<br>2.8.2 Areas for separating and managing patients with a suspected or confirmed contagious disease from those without

PANEL 3. The essential diagnosis-specific care for critically ill patients with COVID-19

CLINICAL PROCESSES

1. The Essential Emergency and Critical Care (EECC) clinical processes as specified for all critical illnesses
2. Personal Protective Equipment (PPE) that is appropriate for COVID-19 as part of Infection, Prevention and Control (IPC)
3. Monitoring oxygen saturation using pulse oximetry at least every 6 hours, unless otherwise prescribed
4. Intermittent prone positioning
5. Low molecular weight heparin or other anticoagulant
6. Corticosteroid
7. Antibiotics in patients with suspected bacterial superinfection

HOSPITAL READINESS REQUIREMENTS

Critically ill patients with COVID-19 require the same hospital readiness for EECC as other critically ill patients. For the provision of the essential diagnosis specific care of critically ill patients with COVID-19, the following additional items are required:

CATEGORY	ITEM
3.1 EQUIPMENT	None
3.2 CONSUMMABLES	3.2.1 Facemasks appropriate for COVID-19 (eg. N95)<br>3.2.2 Eye protection or face shields
3.3 DRUGS	3.3.1 Low-molecular weight heparin (eg. enoxaparin or dalteparin) or other anticoagulant<br>3.3.2 Corticosteroid (eg. dexamethasone)
3.4 HUMAN RSOURCE	3.4.1 Health workers with the ability to care for critically ill COVID-19 patients 24hrs/day
3.5 TRAINING	3.5.1 The health workers are trained in essential care of critically ill COVID-19 patients
3.6 ROUTINES	3.6.1 Routines for care of critically ill COVID-19 patients
3.7 GUIDELINES	3.7.1 Guidelines for essential care of critically ill COVID-19 patients
3.8 INFRASTRUCTURE	3.8.1 Areas for separating and managing patients with suspected or confirmed COVID-19 from those without

Appendix 2: The combined in-depth interview guides (Guide 1 – frontline healthcare workers; Guide 2 – administrative healthcare workers)

2a) GUIDE 1: Interview Guides for Frontline Healthcare Workers

PART 1: Consent for recording

Before we begin, please can you confirm that you consent to having this interview recorded in line with the information sheet shared? Everything you say is confidential, the recorded data will not be shared with anyone outside the research team and the data will be anonymized.

PART 2: Introduction and basic information

Thank you for agreeing to participate in this research. Before we begin, I would just like to emphasize again that this is not an inspection or an audit – and no information specific to this hospital will be fed back to your managers. We are just interested in understanding more about the provision of care for critically ill patients in hospitals. So please feel at ease to be as open as possible with your responses.

**Date for interview**	**Day** [ ] [ ] **Month** [ ] [ ] **Year** [ ] [ ] [ ] [ ]
**Interviewer name**	
**County Name**	
**CODE** E.g. HCW_KE_H1_001_01	

GENERAL INFORMATION ABOUT RESPONDENT

**Gender**	1) Male … 01<br>2) Female… 02
**Age**	
**Job Title**	
**Name of hospital**	
**Name of department**	
**Highest educational qualification achieved?**	
**What kind of medical training do you have?**	
**How many years have you been qualified for this post?**	
**How many years have you been working at this hospital?**	

NOTE TO INTERVIEWER: PROVIDE OUR DEFINITION OF CRITICAL ILLNESS AND EXPLAIN THAT THE INTERVIEW QUESTIONS ARE BASED ON THIS DEFINITION.s

*Critical Illness is a state of ill health with vital organ dysfunction, a high risk of imminent death if action is not taken and the potential for reversibility: so, we mean **all the very sick patients who need immediate care** (and not just the patients in intensive care units or other critical care units).*

- Where are critically ill patients (children, pregnant women, medical adults, surgical adults) cared for in your hospital?
  Probes/clarification if they answer "ICU" or something else. Are critically ill patients cared for:
  - *In the OPD?*
  - *In the Emergency Dept?*
  - *In the general wards?*
  - *Anywhere else in the hospital?*
- How are you involved in the care of critically ill patients?
  Probe:
  - *in what capacity?*

- *In which department?*
- *For how long?*

PART 3: SCENARIO-BASED QUESTIONS

I will now ask a series of questions based on patient scenarios. This is not a test, I am just interested in the systems in your hospital and department. Please answer these questions based on what happens in your hospital, not what should happen based on your clinical training or in textbooks.

3.1 TRIAGE AND ADMISSION

NOTE TO INTERVIEWER: YOU MIGHT SKIP THROUGH RAPIDLY IF THE INTERVIEWEE IS NOT VERY FAMILIAR WITH THIS PROCESS.

Assume a new adult patient arrives at your hospital outpatient department/emergency department (as applicable) with symptoms of severe breathing difficulties.

- Can you describe what happens to this patient on arrival at your hospital?
  Probe:
  - *Who is the first person to help or provide care on arrival? Are they medically trained? Are they emergency medicine trained?*
  - *Registration system (paperwork, payments)?*
  - *Are there delays? Why?*
- How would the severity of this patient's condition be assessed to identify if he/she is critically ill? How does this work?
  Probe:
  - *Who does this?*
  - *Are triage categories used (mention which ones they use)/understood?*
  - Do you look for or screen people who might have signs associated with contagious diseases?
  - *Are Vital sign checked? Which ones?*
- Who is in-charge of triage at the outpatient department? How does this change over shifts?
  Probe:
  - *Night shifts versus day shift*
  - *How are your staff communicating with each other in this department? Are there differences in how staff members communicate with each other?*
- What care do you provide for critically ill patients in the OPD/emergency department in your hospital?
  Probe:
  - *If they become unconscious, what do you do? If they get shock or low blood pressure, what do you do? If their airway becomes unclear, what do you do?*
  - *Where you trained on the approach you use?*
  - *Is there a written guideline or protocol that you follow for unconscious patients?*
- *Is oxygen available in the OPD/emergency department?*
  Probe:
  - *In what form? Concentrators? Cylinders? Piped to bedside?*
  - *Is the supply reliable? How often are there shortages (e.g. weekly).*
  - *How many days in a week do you have patients who need oxygen but you aren't able to provide it?*
  - *How do you decide if the oxygen supply is enough?*
  - *If oxygen runs out or components are missing, what process do you use to report/resolve this?*
  - *If not: why not? How do you manage?*
- Where would this patient be admitted and how would they get there?
  Probe:
  - *Clarify process- straight to ward for admission or via resuscitation room/other?*
  - *Do patients have to wait along the way? And why?*
  - *Does the system for handover and communication with the receiving department/unit/ward work? Why not?*
- Overall, what are the biggest challenges you face during triage and admission of critically ill patients in your hospital?

Probe:

- *Reflect on what has already been discussed: equipment, supplies, communication, drugs, guidelines, routines, training, staffing issues?*

3.2 MANAGEMENT, MONITORING AND ESCALATION OF CARE

NOTE TO INTERVIEWER: YOU MIGHT SKIP THROUGH RAPIDLY OR COMPLETELY DEPENDING ON POSITION OF INTERVIEWEE AND HOW FAMILIAR HE/SHE IS WITH THIS PROCESS. USE YOUR JUDGMENT TO ADJUST QUESTIONS IF REQUIRED, BASED ON WHERE THE INTERVIEWEE WORKS.

*Now, consider the ward or unit where you work the most. Assume this **critically ill** patient has been admitted to your ward/unit with a clinical syndrome consistent with a pneumonia*

- Can you briefly describe the patient handover process when a critically ill patient is admitted at your ward/unit?
  Probe:
  - *Who is involved?*
  - *How do staff communicate with each other both in the department and with other departments?*
  - *Does it work? Why not?*

- After admission, how are critically ill patients checked or monitored for deterioration or improvement?
  Probe:
  - *What monitoring is done? How would you know a patient is deteriorating?*
  - *Are vital signs checked? Which ones?*
  - *How often is this monitoring done? By whom?*
  - *How is this documented and communicated to others on duty?*
  - *Where in your department are they monitored?*
  - *Is someone responsible/in- charge? Who?*
  - Are patients consulted or their opinions asked when the team makes a management decision?
- Is medical oxygen always available in your ward/unit for such patients?
  Probe:
  - *In what form? Concentrators? Cylinders? Piped to bedside?*
  - *Is the supply reliable? How often are there shortages (e.g. weekly). Is it available on all wards?*
  - *If not: why not? How do you manage?*

- What care do you provide for critically ill patients in your ward/unit?
  Probe: if they become unconscious, what do you do? If they get shock or low blood pressure, what do you do? If their airway becomes unclear, what do you do? Are there any written guidelines you follow for this? If so do they work well and how?
- What training have you received to monitor and manage such a critically ill patient?
  Probe:
  - *On the wards, are there any guidelines you follow to monitor and manage patients?*
  - *Who makes decisions about how a patient is monitored in your department?*
  - *Are you confident in being able to identify deterioration? Why/why not?*
  - *Are you confident in being able to care for such patients? Why/why not?*
- What options for escalation of care are available to you, if the patient deteriorates?
  Probe:
  - *CPAP*
  - *High-flow nasal oxygen*
  - *Intubation and mechanical ventilation*
  - *Admission to HDU or ICU*
  - *Referral to another hospital (which hospital?)*
  - *Is the capacity sufficient? Why not?*
- Is there a framework or guideline used in your ward or department to decide when to stop certain treatments?" If so is it helpful?
- Are senior staff available to advise/help for management of critically ill patients?

For peer review only

Probe:
- *Which cadres and types of staff?*
- *Is there a system for alerting them? Please describe.*
- *How effective is this system? Is anyone in-charge of checking it is working?*
- *Differences between night and day shifts?*

- What are the biggest challenges you face when monitoring and managing critically ill patients admitted to your ward/unit?
  Probe: Reflect on what has already been discussed: equipment, supplies, communication, drugs, guidelines, routines, training, staffing issues?

PART 4: GENERAL QUESTIONS

NOTE TO INTERVIEWER: YOU MIGHT NOT HAVE TIME TO DO ALL OF THIS. DO NOT PROBE IF TIME IS VERY LIMITED.

We know that some hospitals have experienced increased cases of patients with respiratory illness in the past 1 year due to COVID-19, and we are interested in how hospitals have managed.

- Did your hospital face any surge in patients with respiratory illness in the past one year due to COVID-19?
- How has care for critically ill patients changed in your facility since the COVID-19 pandemic?
  Probe:
  - *New emergency department or unit?*
  - *New HDU/ICU or increased capacity? Is it in use? Are there any plans to improve its use if its not fully operational? What was the previous capacity?*
  - *Increased number of staff working in critical care, emergency care, anaesthesia? Where have they been placed?*
  - *New equipment or supplies for emergency or critical care? Are they in use? What was you previous equipment capacity on the wards and in OPD/emergency department?*
  - *Increased oxygen capacity? E.g did you get a new oxygen plant? Is it in use?*
  - *Changed processes for identifying and managing patients who need higher levels of care? Has this been beneficial?*
- Have there been changes made to how things are done in this facility or your department in the past (I.e note keeping, handover, handling equipment etc)?
  - If so who started it? How did people receive this change?
  - Why was it successful or not successful?
  -
- Has your hospital received any support (funding/donations in kind/technical support) to strengthen management of the critically ill due to COVID-19? or is any support expected in the coming months?
  Probe:
  - *What support and from whom?*
  - *Do you think it has helped/not helped? Why? (Equipment, drugs, additional staff, supplies, training)*
  - *If no: has this been challenging?*
  - *How did your hospital cope e.g. repurpose staff, longer shifts, borrow equipment from other departments?*
- Compared to pre COVID-19 pandemic, do you think your hospital is more ready to identify, care for and monitor critically ill patients?
  Probe:
  - *If yes/no, how and why?*
- Are there any important issues that still need to be fixed, which you haven't already mentioned?
  Probe:
  - *Equipment, supplies, staff, training, routines/guidelines, communication, systems*
- Finally, if we want to run a program to improve the management of all critically ill patients, what would you recommend that we do?
  Probe:
  - *What additional support would have the most impact/be most helpful?*
  - *How would this improve the care of critically ill patients*

Thank you very much for your time.

2b) GUIDE 2: Interview Guide for Administrative Healthcare Workers- Kenya

PART 1: Consent for recording

Before we begin, please can you confirm that you consent to having this interview recorded in line with the information sheet shared? Everything you say is confidential, the recorded data will not be shared with anyone outside the research team and the data will be anonymized.

PART 2: Introduction and basic information

Thank you for agreeing to participate in this research. Before we begin, I would just like to emphasize again that this is not an inspection or an audit – and no information specific to this hospital will be fed back to your managers. We are just interested in understanding more about the provision of care for critically ill patients in hospitals. So please feel at ease to be as open as possible with your responses.

**Date for interview**	**Day** [ ] [ ] **Month** [ ] [ ] **Year** [ ] [ ] [ ] [ ]
**Interviewer name**	
**County Name**	
**CODE** E.g. HCW_KE_H1_001_01	

GENERAL INFORMATION ABOUT RESPONDENT

**Gender**	3) Male … 01<br>4) Female… 02
**Age**	
**Job Title**	
**Name of hospital**	
**Name of department**	
**Highest educational qualification achieved?**	
**What kind of medical training do you have?**	
**How many years have you been qualified for this post?**	
**How many years have you been working at this hospital?**	

NOTE TO INTERVIEWER: PROVIDE OUR DEFINITION OF CRITICAL ILLNESS AND EXPLAIN THAT THE INTERVIEW QUESTIONS ARE BASED ON THIS DEFINITION.

Critical Illness is a state of ill health with vital organ dysfunction, a high risk of imminent death if action is not taken and the potential for reversibility: so, we mean ***all the very sick patients who need immediate care*** *(and not just the patients in intensive care units or other critical care units).*

PART 3: SCENARIO-BASED QUESTIONS

I will now ask a series of questions based on patient scenarios. This is not a test, I am just interested in the systems in your hospital and department. Please answer these questions based on what happens in your hospital, not what should happen based on your clinical training or in textbooks.

3.1 TRIAGE AND ADMISSION

NOTE TO INTERVIEWER: YOU MIGHT SKIP THROUGH RAPIDLY IF THE INTERVIEWEE IS NOT VERY FAMILIAR WITH THIS PROCESS.

Assume a new adult patient arrives at your hospital outpatient department/emergency department (as applicable) with symptoms of severe breathing difficulties.

- Can you describe what happens to this patient on arrival at your hospital?
  Probe:
  - *Who is the first person to help or provide care on arrival? Are they medically trained? Are they emergency medicine trained?*
  - *Registration system (paperwork, payments)? How do you decide which payments must be made upfront?*
- Who decides which systems are used to identify the severity of this patient's condition if he/she is critically ill? How does this work?
  Probe:
  - *Are there staff trainings in this department on systems to manage critically unwell patients?*
  - *Who is responsible for training staff on which systems to use?*
  - *Are anaesthetists or critical care staff involved in training staff in your department on critical illness management?*
  - *Who ensures staff have the equipment they need to use these systems?*
- Who is in-charge of triage at the outpatient department? How does this change over shifts?
  Probe:
  - *Night shifts versus day shift*
  - *Who decides how many staff members are needed for each shift?*
- *Is oxygen available in the OPD/emergency department?*
  Probe:
  - *In what form? Concentrators? Cylinders? Piped to bedside?*
  - *Is the supply reliable? How often are there shortages (e.g. weekly).*
  - *Who is responsible for monitoring oxygen supply? Who maintains the oxygen delivery systems?*
- If this patient was admitted to a ward, how would staff communicate between departments?
  Probe:
  - *Who is responsible for setting up and maintaining communication systems?*

3.2 MANAGEMENT, MONITORING AND ESCALATION OF CARE

NOTE TO INTERVIEWER: YOU MIGHT SKIP THROUGH RAPIDLY OR COMPLETELY DEPENDING ON POSITION OF INTERVIEWEE AND HOW FAMILIAR HE/SHE IS WITH THIS PROCESS. USE YOUR JUDGMENT TO ADJUST QUESTIONS IF REQUIRED, BASED ON WHERE THE INTERVIEWEE WORKS.

*Now, consider the ward or unit where you work the most. Assume this **critically ill** patient has been admitted to your ward/unit with a clinical syndrome consistent with pneumonia*

- After admission, how are critically ill patients checked or monitored for deterioration or improvement?
  Probe:
  - *What monitoring is done? How would staff know a patient is deteriorating?*
  - *Are vital signs checked? Which?*
  - *How often is this monitoring done? By whom?*
  - *How is this documented and communicated to others on duty?*
  - *Where in your department are they monitored?*
  - *Is someone responsible/in- charge? Who?*
- Is medical oxygen always available in your ward/unit for such patients?
  Probe:
  - *In what form? Concentrators? Cylinders? Piped to bedside?*
  - *Is the supply reliable? How often are there shortages (e.g. weekly). Is it available in all wards?*
  - *Who is responsible for monitoring oxygen supply? Who maintains the oxygen delivery systems?*

For peer review only

- What training have staff received to monitor and manage such a critically ill patient?
  Probe:
  - *When did they receive this training?*
  - *Do they receive refresher sessions? How often?*
  - *Who decides which staff should undergo training?*
- What options for escalation of care are available to you, if the patient deteriorates?
  Probe:
  - *Is there a guideline or SOP staff use to escalate care?*
  - *Who decides which guidelines are used?*
- Is there a framework or guideline used in your ward or department to decide when to stop certain treatments?"

PART 4: GENERAL QUESTIONS

NOTE TO INTERVIEWER: YOU MIGHT NOT HAVE TIME TO DO ALL OF THIS. DO NOT PROBE IF TIME IS VERY LIMITED.

We know that some hospitals have experienced increased cases of patients with respiratory illness in the past 1 year due to COVID-19, and we are interested in how hospitals have managed.

- Did your hospital face any surge in patients with respiratory illness in the past one year due to COVID-19?
- How has care for critically ill patients changed in your facility since the COVID-19 pandemic?
  Probe:
  - *New emergency department or unit?*
  - *New HDU/ICU or increased capacity? Is it in use? Are there any plans to improve its use if its not fully operational? What was the previous capacity?*
  - *Increased number of staff working in critical care, emergency care, anaesthesia? Where have they been placed?*
  - *New equipment or supplies for emergency or critical care? Are they in use? What was you previous equipment capacity on the wards and in OPD/emergency department?*
  - *Increased oxygen capacity? E.g did you get a new oxygen plant? Is it in use?*
  - *Changed processes for identifying and managing patients who need higher levels of care? Has this been beneficial?*
- Have there been changes made to how things are done in this facility or your department in the past (I.e note keeping, handover, handling equipment etc)?
  - If so who started it? How did people receive this change?
  - Why was it successful or not successful?
- Has your hospital received any support (funding/donations in kind/technical support) to strengthen management of the critically ill due to COVID-19? or is any support expected in the coming months?
  Probe:
  - *What support and from whom?*
  - *Do you think it has helped/not helped? Why? (Equipment, drugs, additional staff, supplies, training)*
  - *If no: has this been challenging?*
  - *How did your hospital cope e.g. repurpose staff, longer shifts, borrow equipment from other departments?*
- Finally, if we want to run a program to improve the management of all critically ill patients, what would you recommend that we do?
  Probe:
  - *What additional support would have the most impact/be most helpful?*
  - *How would this improve the care of critically ill patients?*

Thank you very much for your time.

For peer review only

Appendix 3: Process maps

Appendix 3a: Process map – Facility 1

Entry and triage

- The hospital receives patients either as walk-ins/referrals
- The triage nurse sorts patients to their respective care points
- Preliminary vital signs are taken by the triage nurse

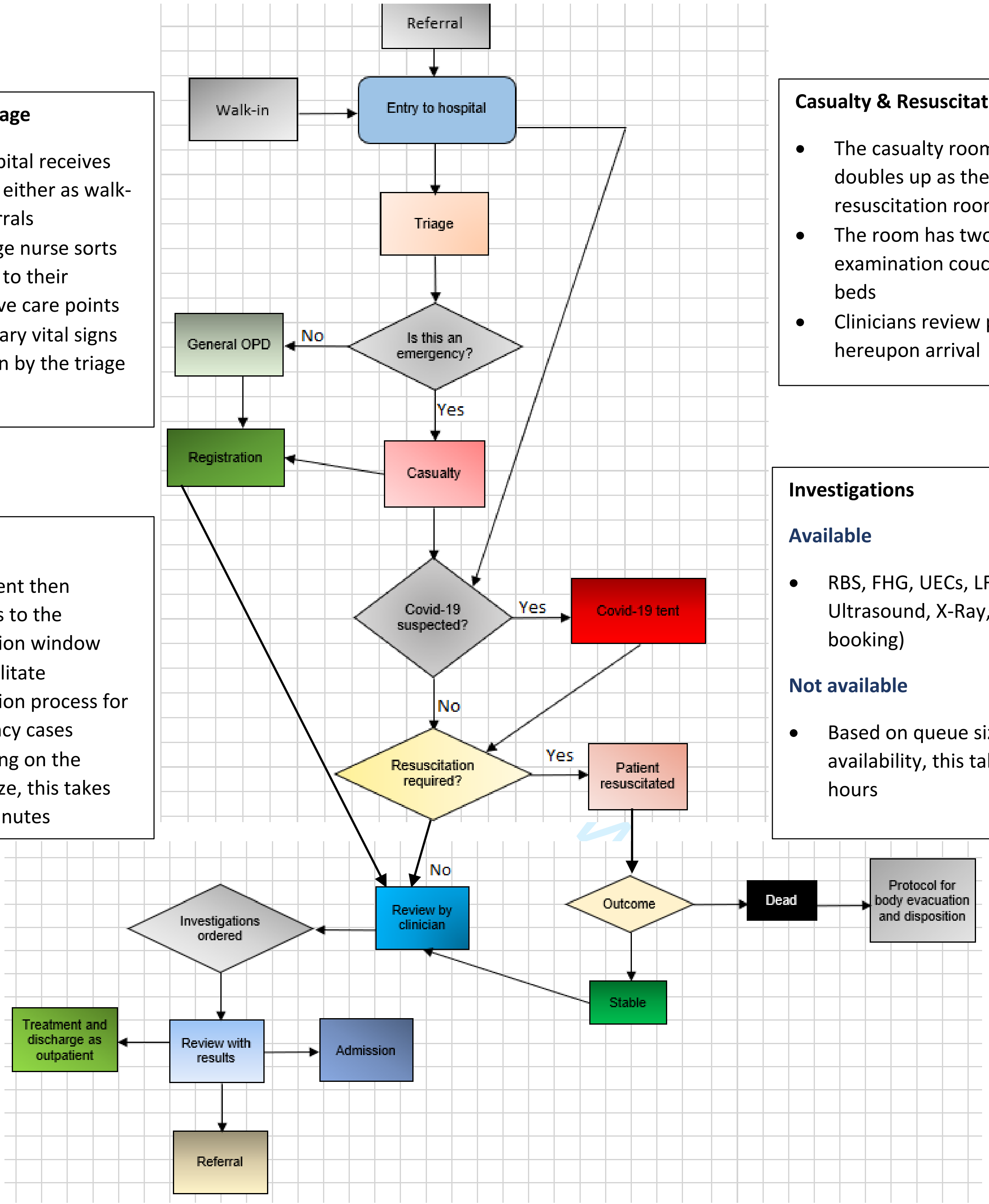

Casualty & Resuscitation

- The casualty room also doubles up as the resuscitation room
- The room has two examination couches and 5 beds
- Clinicians review patients hereupon arrival

Registration

- The patient then proceeds to the registration window
- Staff facilitate registration process for emergency cases
- Depending on the queue size, this takes 10-45 minutes

Investigations

Available

- RBS, FHG, UECs, LFTs, Ultrasound, X-Ray, MRI (upon booking)

Not available

- Based on queue size and availability, this takes 1-4 hours

Consultation protocol

- Clinical Officer (+/- clinical officer interns) sees patient
- Clinical Officer calls Medical Officer/registrar
- MO/registrar calls consultant

Appendix 3b: Process map – Facility 3

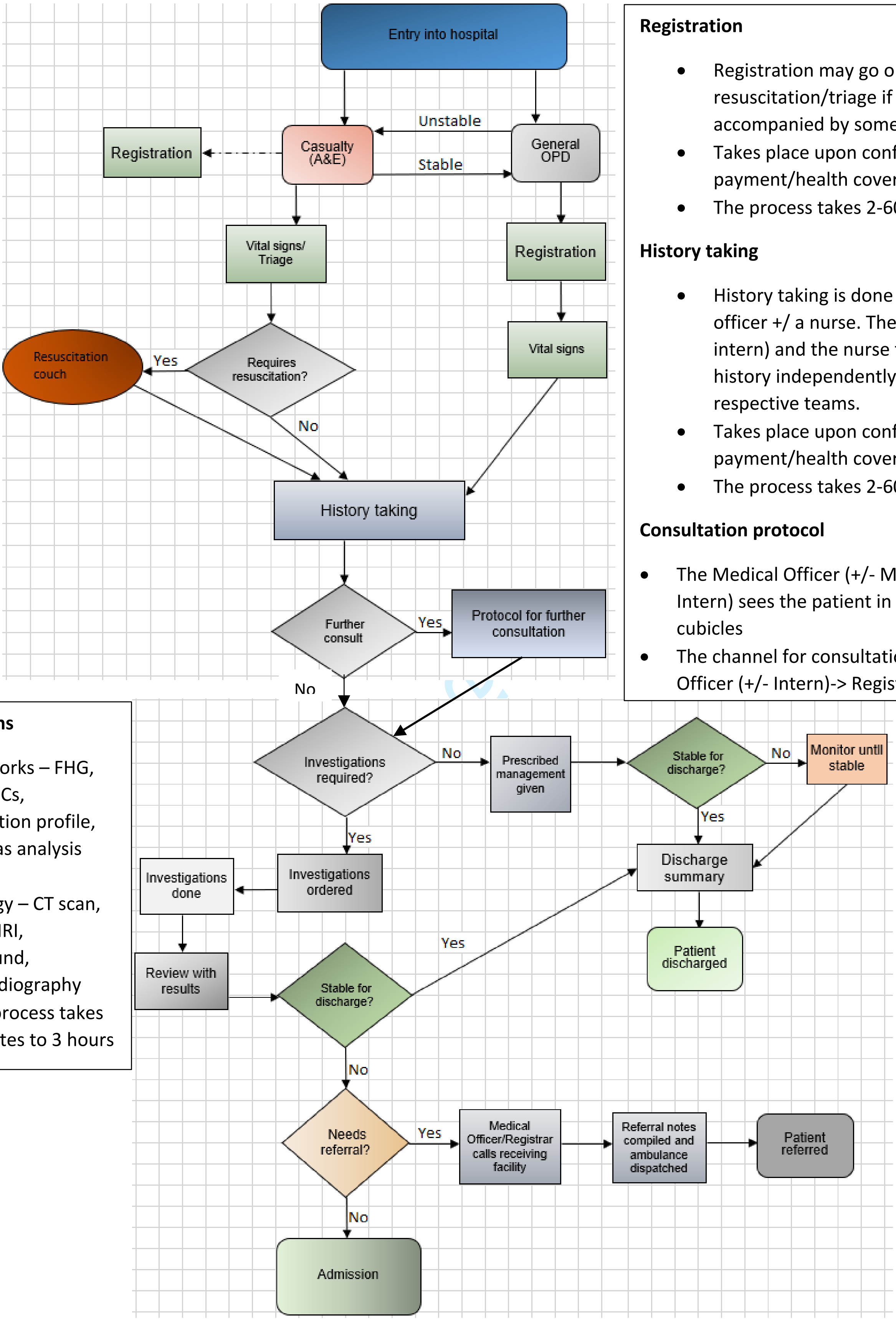

Registration

- Registration may go on during resuscitation/triage if the patient is accompanied by someone
- Takes place upon confirmation of payment/health cover
- The process takes 2-60 minutes

History taking

- History taking is done by a medical officer +/ a nurse. The MO (or MO + MO intern) and the nurse take patient history independently for their respective teams.
- Takes place upon confirmation of payment/health cover
- The process takes 2-60 minutes

Consultation protocol

- The Medical Officer (+/- Medical Officer Intern) sees the patient in the consultation cubicles
- The channel for consultation is Medical Officer (+/- Intern)-> Registrar -> Consultant

Investigations

- Blood works – FHG, LFTs, UECs, Coagulation profile, Blood gas analysis (BGA)
- Radiology – CT scan, X-ray, MRI, Ultrasound, Echocardiography
- Whole process takes 30 minutes to 3 hours

Appendix 4: Patient Journeys

4a) Journey of a patient presenting with severe abdominal pains (later diagnosed with ruptured ectopic pregnancy) at Facility 1.

Date: September 2, 2021

Narrator: OO, Assistant Research Officer – POETIC-COVID project

10:20 am: We have just received an adult female patient – she has been brought in [at the A&E] accompanied by two relatives. She is in obvious distress and reports symptoms of severe progressive abdominal pain. She has been put on a bed – the nurse and a clinician (Clinical Officer) are already with her. I'll keep you posted.

10:24 am: The vitals have been taken: SPO2 is 93% on room air, the heartrate is 90 b/min. Other parameters seem, okay? A thing to note is that this unit, the ER, has five couches, four oxygen ports and there are two bedside monitors that are operational. So, I'll keep you posted.

10:30 am: The nurse is fixing an IV line, together with the Clinical Officer.

10:34 am: The Clinical Officer has written a lab request for a blood test [full hemogram]. The nurse has drawn a blood sample to be taken to the lab. So, the patient is still in bed.

10:40 am: The nurse has repeated the vital signs; BP is 93/50mmHg. The nurse is putting up IV normal saline [500 ml] right now.

10:57 am: Status update: patient still in bed, IV fluid running. No other consultations made.

11:20 am: The first bottle of IV is over, the nurse has put up a second 500ml bottle of normal saline. The relatives have been asked to go make payments for the blood sample to be taken to the lab for processing. They are yet to make the payments.

11:30 am: The second IV is running well. We still await review. Notable is that the unit is favourably staffed, I can count five nurses in the shift – although one of them is the unit in-charge, but the other four are pretty much on the ground – also two Clinical Officers and one Medical Officer.

11:35 am: The relatives have gone to make payments, after which the blood sample will be taken to the lab.

12:04 pm: The relatives are back with the receipt of payment. So, any time now the sample will be taken to the lab.

12:21 pm: The second IV normal saline is still running. Vital signs have been repeated: BP is 98/59mmHg. Also, on assessment, the patient was noted to be pale. We still await the results from the lab.

12:45 pm: The Medical Officer has reviewed the patient and has requested for an abdominopelvic ultrasound to determine what could be the cause of her discomfort and symptoms. The IV is still running. Meanwhile, the relatives have been asked to go make payment for the radiological tests.

1:07 pm: The second IV fluid is still running. It is around the change-over time, new staff are coming in for the afternoon shift. They are sitting together, and a hand-over process is going on - the new staff are being briefed on the patients' conditions – so we hope that things are going to be swift and the patient is going to receive the care soonest possible.

For peer review only - http://bmjopen.bmj.com/site/about/guidelines.xhtml

1:14 pm: The patient is being wheeled to the radiology department for the abdominopelvic ultrasound. She's accompanied by her relatives. Payment to be made at the cashier's window next to the department.

1:15 pm: We are at the radiology department: there is a queue, so, the patient will have to wait outside on the wheelchair before she's seen. Payment is being made.

1:45 pm: One relative [patient's sister] has come back to the casualty [A&E]. Apparently, she has been told that the test imaging (abdominal ultrasound) cannot be done until a later time, maybe even tomorrow since the patient is required to have fasted before the radiological test. We are yet to establish if there's anything else that can be done.

1:52 pm: The doctor has instructed the patient's sister to communicate to the radiology department technicians that the test is urgent and that this patient is due for admission into the ward. We are hoping that the patient is going to get the test done soon. The patient is still waiting outside the radiology department on a wheelchair in the company of the other relative.

1:57 pm: The patient has been wheeled back to the casualty department, but the test has not been done because the radiology team want a clear communication from the casualty on the urgency of the test.

2:05 pm: The doctor has indicated [in writing] in the patient's file that the test request is URGENT, and it needs to be done as soon as possible. That communication is being relayed to the radiology through the file that is to be taken there by the patient's relatives. The patient is being wheeled back to the radiology department. We hope that the test is going to be done and the results to be out soon because this patient is supposed to be admitted.

2:15 pm: The patient has been wheeled back again into the A&E from the radiology department. The test has not been done. The radiology team holds that the abdominal ultrasound cannot be done right now because the patient is needed to have fasted so it will have to be done later. The doctor at the A&E has reviewed the initial request and ordered specifically for a pelvic ultrasound. The patient is to be taken for a pelvic ultrasound soon.

2:25 pm: The patient has been wheeled to the radiology department again, with the new request, so we await feedback. We hope that the test is going to be done so she can proceed to the next care point. They are to make payment for the new test and reverse the initial payment.

2:35 pm: Patient is in the waiting bay at the radiology department. She has been instructed to take some water (has a litre of water) to have a full bladder to aid the pelvic ultrasound. The patient's sister has gone to make the payment.

3:03 pm: The patient is still waiting outside. The waiting area seems packed; a lot of patients waiting to be seen. We are just hoping that the patient will be in soon.

3:37 pm: Status update: not much has moved, the patient is still waiting outside.

5:20 pm: Patient still waiting in the waiting bay. She is next in line to be seen, likely in the next ten or less minutes.

5:33 pm: The patient has just been wheeled into the radiology room.

5:50 pm: The patient has just left the radiology room. The results are expected to be out within the next 30 minutes, so they'll have to wait a little longer after she leaves the radiology department.

6:17 pm: Final update for Patient 1. The patient has been wheeled back to the casualty, awaiting review with the radiology results. This marks the end of my follow-up with this patient because of time but from

For peer review only

the look of things the doctor says the patient is to be admitted, although that is after review with results once the results are out.

Follow up call at 8:26 am - Friday September 3: The patient was admitted in the gynaecological ward at night around 10:30pm with a diagnosis of a ruptured ectopic pregnancy and was taken to theatre for urgent surgical intervention [exploratory laparotomy].

4b) An adult male patient brought in unconscious at Facility 2 (Case 2 in the main document).

Date: September 13, 2021

Narrator: OO, Assistant Research Officer – POETIC-COVID project.

12:06 pm: I am at Facility 2. The patient has just been brought in, carried by two relatives. He looks unconscious. The patient has been put on a chair in the ER. The ER has two rooms; A& B, each with 4 beds.

12:08 pm: The nurse and two EMTs are setting up to take vital signs. The random blood sugar (RBS) has just been taken and is 8.5 mmol/L. The patient is still unresponsive. Meanwhile, the relatives have been asked to go start the registration process.

12:10 pm: The vitals seem okay except for the SPO_2: the BP is 130/75mmHg, HR is 81b/min and SPO_2 is 85% on room air. The ER doesn't have piped oxygen, but we have two huge oxygen tanks with flowmeters attached (no flow splitters). Right now, the two cylinders are both in use. The room has two monitors with one SPO_2 probe attached to one of the monitors. The other SPO_2 probe is in use at the triage area.

12:19 pm: The patient has been taken to the other room, where a space has been created in a bed. The patient is yet to be reviewed; the relatives are not yet back from the registration office.

12:22 pm: The patient has been put onto a bed in the other room, but he's sharing the bed with another patient since the space is full. This room also has 4 beds that are currently all occupied. Two more patients are sitting in chairs with IV drips. This cubicle has one oxygen tank with a capacity of about 50.2L.

12:31 pm: The Medical Officer and two EMTs are with the patient. The EMTs are fixing an IV cannula and drawing a blood sample into two vacutainers.

12:35 pm: The blood samples have been drawn into the two sample containers. The Medical Officer has drawn up a lab request and handed it to the patient's relative to deliver to the lab upon making the payments. The patient is still in bed and awaits further review.

12:55 pm: A 500ml bottle of IV Ringer's Lactate has been set up and is running well. The patient is yet to be reviewed.

1:16 pm: The IV is over and the patient still awaits further review. The patient is still in distress. No further vital signs have been taken. We await the next course of steps. The ER is quite busy, and there's a queue outside of patients waiting to be seen. The two rooms in the ER are full as well, with patients sharing beds. We are just hoping that the patient is going to be reviewed soon.

1:25 pm: The patient has been noticed to be having convulsions. We still haven't had a concrete review because the doctor is quite held up and the patient's details have not been updated.

1:40 pm: The doctor has prescribed an anticonvulsant. The prescription has been handed to a relative to pick up the drug from the pharmacy upon payment. The unit is still very busy, with very sick patients being wheeled in.

For peer review only

2:41 pm: The medication has just been brought and will be administered any time now. The patient is still in bed in status quo; still unresponsive and having convulsions. The results are not yet out.

2:56 pm: The nurse has just given the injection of the anticonvulsant, so, right now we await that to take effect. We hope the patient will be reviewed soon; the lab results are still not out.

3:50 pm: The results are still not out, and the patient still awaits a review. No further vital observations have been taken.

4:15 pm: The patient hasn't been reviewed yet; the results are yet to be received back from the lab. No further vitals have been taken. The patient's condition hasn't improved much, but the convulsions are not as strong as they were before. This is the final update for today because of time.

4c) Journey of a patient who presented with symptoms of severe difficulty in breathing (Covid-19 patient) at Facility 4 (Case 3 in the main document).

Facility: Facility 4

Date: June 9, 2021

Narrator: OO, Assistant Research Officer – POETIC-COVID project.

0830h: Morning. This is OO, the ARO for the POETIC-COVID study. It is about 8:30 am, a patient has just been wheeled into the casualty [ER] with apparent symptoms of severe breathing difficulties. The patient is being wheeled onto a couch in the casualty. The Medical Officer is still not yet in. The doctor [Medical Officer] was here overnight, so he has stepped out to refresh before coming back. Upon coming back, we hope the patient will be reviewed and if at all there are any investigations to be taken the patient will be taken accordingly. That's all for now, we wait for the next steps.

0835h: Update for Patient 2, it is 8:35 am. The vitals have just been taken. It has been noted that the heartrate is a little unstable, ranging as low as from 50s to 60s but at times it's spiking, so not really stable. She's in obvious breathing difficulty [distress]. The SPO2 currently is reading 58% off oxygen. To be noted again is that the unit has one pulse oximeter that is operational. There is another one attached to the monitor but not working. The other patients…there is one other patient who needs constant pulse oximetry, but he has his own…the family came with a personal pulse oximeter, so I think for that he's sorted for now. That is allfor now, I'll keep you posted.

0900h: Update for Patient 2, it's 9 am. The doctor arrived at around 8:57 am. He has reviewed the patient as well as the others. He's suggesting an X-ray, so the relatives have gone to the registration window to open a file for the patient. So, we await that after which the prescribed test [X-ray] …she'll be wheeled in.

0915h: Patient 2, the time is 9:15 am. The patient has just been wheeled in for the X-ray. Being that she isamong the very first patients who reported to this casualty we are hoping that it won't take long, she's going to be attended to almost immediately. So, we hope that she'll be out in the next few minutes.

0937h: The patient has just left the X-ray department, awaiting review with results [results are not out yet]…so, the patient is back in the casualty. I'll keep you posted. It's now 9:37 am, the patient went in at 9:15 am and is already out. So, we'll follow up upon review of the results.

0950h: The patient has been put on oxygen, in bed at the casualty department awaiting the X-ray results. Not really connected to a monitor because the monitors are faulty, but the patient is on oxygen, so from time to time the vitals are being checked. I'll keep you posted.

1015h: The results are out…the X-ray results are out but apparently there has to be a Covid test done. TheRDT [Covid rapid test] is not available in that department so the patient has to be taken away again; a stone-throw away within the hospital for the test. The relatives are still following up with whoever is responsibleon how best that can be done, but otherwise, the patient is still on oxygen, we are awaiting a review of theX-ray results. There is a queue…there's one patient ahead so the Medical Officer is still with another patientbut once he's done, I think the patient will be reviewed with the X-ray results. However, we are still following up with the Covid team for the Covid test. I'll keep you posted.

1043h: Update for Patient 2, 10:43 am. The Covid team came to take samples for the rapid test from the casualty. The samples are being taken, so we await the next few minutes for the results to be out after

which it'll inform the direction of care; whether this patient is going to be admitted in the medical ward orthe Covid isolation ward. I'll keep you posted, that's the update for now.

1127h: Update for Patient 2, it is 11:27am. The test results for Covid-19 are out, she has been confirmed tobe Covid positive. So, the patient has been indicated to be admitted in the Covid isolation ward. Initially they had planned to admit her in the female medical ward, but now that the results are out, she's going tobe taken to the isolation unit. One thing about the isolation unit (I was already there a just a while ago), it has a capacity of six [6]. It has a total of 6 beds. At the moment four [4] of those are occupied, so we are hoping that this patient is going to get space. We are just hopeful that the space avails…we keep on waitingat the casualty. The patient is still on oxygen via face mask, the vitals-the SPO2 is okay. She's saturating on97% on oxygen, but the pulse rate was a little worrisome initially. It was going as low as 52, at one point even at 43 beats per minute but at the moment we cannot say that she's so badly off. She's still on oxygen, pulse rising to above 68. That's the update for now.

1140h: Update for Patient 2. The vitals have been taken again…still the SPO2 is ranging between 90-93% onoxygen. To be noted again is that there is no high-flow oxygen in this facility due to the oxygen supply [strained]. Again, in the casualty there is on oxygen port which is being used to give oxygen to this patient via mask. There's no non-rebreather mask in the department as at now but hopefully the SPO2 will rise, it is promising. Again, this unit is actually very full at this point, remember there is only one medical officer. The unit is full and other patients are actually complaining. Also there's another patient who is having similar issues [as Patient 2] and they [the medical team] were trying to locate a bed space for the patient…there has been a push-and-pull between the Medical Officer here and the destination hospital [FACILITY X], they have been arguing over phone and the Medical Officer seems frustrated here. Looks like he feels his hands are tied and the patients need help and they cannot be able to help but we are hoping that things ease up over the next few minutes.

1254h: Update for Patient 2, the time is 12:54 pm. It has been confirmed that there is bed space for this patient in the Covid isolation ward in this Facility. Her latest SP02 was 94%. So, we are waiting for a stretcherto be brought so that the patient can be wheeled there.

1304h: Update for Patient 2, the time is 1304h. The patient has just been put on the stretcher to be takento the Covid isolation ward…the patient is being admitted in the Covid isolation ward. The patient is to be taken there on portable oxygen in a cylinder. They're setting that up. I'll keep you posted once the patientlands in the Covid isolation ward. They have just left the casualty.

For peer review only

1319h: Update for Patient 2, the time is 1319h. The patient has just been transferred to the Covid isolation unit...patient has been admitted. To be noted is that this patient is currently saturating at 94% on oxygen but on admission [when she landed at the casualty] the patient was actually saturating as low as 17% - thatwas off oxygen. The patient has been maintained in the casualty on oxygen and has been transferred into the Covid isolation unit. As it stands the patient has been admitted into the isolation unit with Covid-19 Pneumonia. I'll halt there for now, that marks the end of my journey with

4d) An adult male patient brought in unconscious at Facility 2.

Facility 2
Date: September 13, 2021
Narrator: OO, Assistant Research Officer – POETIC-COVID project.

11:40 am: I am in the A&E department at Facility 2. The ER has two rooms: A & B, each with 4 beds. At the moment all eight beds are occupied. There's no piped oxygen in the department but I can count three oxygen cylinders with a capacity of 50.2L each. Right now, two of the cylinders are in use. Each cylinder has a flowmeter attached, but no flow splitters. The cylinders serve one patient at a time.

11:45 am: An adult male patient has been brought in, carried by two relatives, apparently unconscious.

11:48 am: The patient is in a chair (all 8 beds are currently occupied), still not responsive. The nurse and an assistant - looks like a trained Patient Attendant or an EMT – are attending to the patient. They are setting up to take the patient's vital signs. The SPO2 probe has just been put. The EMT has fixed an IV line.
The RBS is 3.4 mmol/L. A blood sample has been drawn, to be taken to the lab upon payment by the relatives. I'll keep you posted.

12:15 pm: The patient is still in a chair since the 4 beds in this cubicle are occupied. There is an adjacent cubicle but that as well is currently full. The nurse and an EMT are trying to set up an IV fluid, but they first have to settle the patient in a bed. They are trying to create a bed space by relocating another patient who has stabilized. Just to add, this department has 3 nurses, 1 Medical Officer and 2 Emergency Medical Technicians (EMTs). The EMT assist with wheeling the patients as well as fixing IV lines.

12:21 pm: The patient has been put onto a bed, but he's sharing the bed with another patient since the space is full. The nurse has just put up a 500 ml bottle of 5% IV Dextrose. The patient's relatives have been given the sample to deliver to the lab upon making payment at the laboratory department.

12:40 pm: The IV Dextrose is still running. No further review at this point. The department is quite busyand packed with patients. The results are not yet out from the lab.

12:48 pm: The patient is currently responsive; he is waking up. The IV Dextrose is still running, but still no further review yet.

1:15 pm: The IV fluid (5% Dextrose) is over. The patient is awake and looks stable. We still await review. The lab results are not out yet. The patient has been taken outside to wait on the bench.

1:50 pm: The patient is stable and has been asked to wait outside on the bench in the waiting bay so as tocreate a bed space for another patient. The results are not ready from the lab. The patient will be reviewed once the results are out.

For peer review only

4e) An adult female patient brought in by social worker with symptoms of respiratory distress at Facility 2. Observation was concurrent with 4b above.

Facility: Facility 2
Date: September 13, 2021
Narrator: OO

12:13 pm: This is a new patient – patient 3. The patient is an adult female. She has been brought to the ER in the company of the City County personnel and a Social Worker from the hospital. She is from a street family. She is in obvious distress; generally, she looks unwell and seems to be having respiratory problems. She has been put in a chair since all beds are currently full. We await further steps.

12:31 pm: The patient is still in obvious respiratory distress. She has not been reviewed yet. The patient is alone right now (not in the company of anyone – she is from the street families). She's sitting in a chair but still inobvious distress. I can see she's carrying some medications in her hands, seems to have been seen elsewhere prior to being brought here. She also has an IV cannula already fixed in her arm, apparently fixed outside the hospital. We hope she gets seen soon.

12:41 pm: The patient has just been seen, the vitals have been taken; BP seems to be 110/52 mmHg, HRis 109 b/min and SPO2 is 78% on room air. She's still in a chair, yet to be reviewed further. The beds arestill full, so the patient will have to wait in this chair a little longer.

1:07 pm: The patient has been put on oxygen via face mask. She's still seated on the chair. To note is thatthis patient seems to be having altered mental status with incoherent speech. She's still by herself but occasionally the EMTs are checking up on her, and she's yet to be evaluated in the next round of vital signs. Right now she doesn't seem good: still in severe respiratory distress and altered mental status.

1:15 pm: Patient is still on oxygen, status quo. The nurse has noticed that the patient keeps pulling off the oxygen mask from her face, doesn't want the mask put on her face. The patient still hasn't been reviewed by the doctor. No samples or further vitals have been taken. She's still on oxygen.

1:37 pm: The oxygen has been discontinued for now since the patient is uncooperative. The patient is still in a chair, no improvement. We still await a review, hopefully she'll get seen soon.

1:43 pm: The patient has been noted to have fallen off the chair and is lying on the floor, unresponsive. I have alerted the nurse and she has come accompanied with an EMT. The nurse and the EMT are surrounding the patient, trying to assist. The patient is unresponsive. We hope she's okay.

1:47 pm: The patient has just been confirmed dead. Just to repeat, the patient was brought in by a socialworker and the Nairobi City County team. She has no relatives as of this moment. The morgue is being informed shortly; the body is still on the floor in the ER.

3:00 pm: The body is being prepared for transport to the morgue. This marks the end of my follow-upwith the patient.

For peer review only

4f) Asample patient journey from the perspective of healthcare workers at Hospital 2 of a hypothetical patient requiring X-Ray, CT scan and ICU admission

Step	Activity	Minimum time (minutes)	Maximum time (minutes)
1	Patient arrives in the casualty department		
2	Patient is received by the triage nurse	2	30
3≠	Preliminary vital signs are taken by the nurse	1	2
4	Patient waits in the queue at the registration window	1	30
5 ≠	Confirmation of patient health cover/ payment of registration fee	2	5
6	Patient registration	5	10
7	Waiting in the queue for vital signs checking	1	30
8≠	Vital signs repeated and recorded by nurse	2	5
9	History taking by nurse	5	20
10	Waiting in the queue for review by clinician	5	45
11	Examination by clinician	5	15
12	History taking by clinician	5	15
13	Investigations (X-Ray and CT Scan) ordered	2	2
14	Payment of X-Ray fee at the cashier	5	45
15	Waiting in queue for X-Ray	15	120
16≠	Transport outside facility to seek the CT Scan	15	60
17	Waiting in queue for CT Scan	30	120
18≠	Waiting for interpretation of results	15	60
19	Travel back for review with CT Scan results	15	60
20	Waiting in queue for review with X-Ray and /CT Scan results	10	60
21	Doctor's review with results	10	45
22≠	Doctor calls a referral facility for ICU bed space	1	10
23	Waiting for availability of bed space (ICU)*	30	120
24≠	Ambulance set up and patient transported to destination facility	45	120

= waiting, ≠ = potential bottleneck

Registration is usually facilitated for a critically ill patient not accompanied by a relative

*Based on availability of ICU beds, the patient may wait for up to two hours or longer. In the event that no ICU space is available in the public health facilities the patient will have to source for space in private ICUs or wait until one avails in the public hospitals.

Consultation protocol

- Clinical officer (+/- clinical officer interns) sees patient
- Clinical officer calls Medical officer/registrar
- MO/registrar calls consultant

Investigations

- Available tests: RBS, FHG, UECs, LFTs, Ultrasound, X-Ray

For peer review only

- Not available: CT-Scan, MRI

Referral protocol (similar to Facility 1 in the main text)

- Through respective specialties;
- Consultant reviews patient, decides referral
- Unit manager notified
- MO/ Registrar calls destination hospital with patient details
- Ambulance set up and patient referred

For peer review only

Standards for Reporting Qualitative Research (SRQR)*

http://www.equator-network.org/reporting-guidelines/srqr/

Page/line no(s).

Title and abstract

**Title** - Concise description of the nature and topic of the study Identifying the study as qualitative or indicating the approach (e.g., ethnography, grounded theory) or data collection methods (e.g., interview, focus group) is recommended	**Page 1/**line 1-2
**Abstract** - Summary of key elements of the study using the abstract format of the intended publication; typically includes background, purpose, methods, results, and conclusions	**Page 2/**line 1-35

Introduction

**Problem formulation** - Description and significance of the problem/phenomenon studied; review of relevant theory and empirical work; problem statement	**Page 4/**line 1-48
**Purpose or research question** - Purpose of the study and specific objectives or questions	**Page 4/**line 48- **Page 5/**line 1-2

Methods

**Qualitative approach and research paradigm** - Qualitative approach (e.g., ethnography, grounded theory, case study, phenomenology, narrative research) and guiding theory if appropriate; identifying the research paradigm (e.g., postpositivist, constructivist/ interpretivist) is also recommended; rationale**	**Page 6/**line 3-9
**Researcher characteristics and reflexivity** - Researchers' characteristics that may influence the research, including personal attributes, qualifications/experience, relationship with participants, assumptions, and/or presuppositions; potential or actual interaction between researchers' characteristics and the research questions, approach, methods, results, and/or transferability	**Page 7/**line 40-47
**Context** - Setting/site and salient contextual factors; rationale**	**Page 6/**line 11-30
**Sampling strategy** - How and why research participants, documents, or events were selected; criteria for deciding when no further sampling was necessary (e.g., sampling saturation); rationale**	**Page 7/**line 1-17, **Page 8/**line 1-3
**Ethical issues pertaining to human subjects** - Documentation of approval by an appropriate ethics review board and participant consent, or explanation for lack thereof; other confidentiality and data security issues	**Page 7/**line 7-12 and **Page 20/**line 33-37
**Data collection methods** - Types of data collected; details of data collection procedures including (as appropriate) start and stop dates of data collection and analysis, iterative process, triangulation of sources/methods, and modification of procedures in response to evolving study findings; rationale**	**Page 7/**line 19-47 and **Page 8/**line 1-15

For peer review only

For peer review only - http://bmjopen.bmj.com/site/about/guidelines.xhtml

**Data collection instruments and technologies** - Description of instruments (e.g., interview guides, questionnaires) and devices (e.g., audio recorders) used for data collection; if/how the instrument(s) changed over the course of the study	**Page 7/**line 21-22 and **Page 8/**line 5-7
**Units of study** - Number and relevant characteristics of participants, documents, or events included in the study; level of participation (could be reported in results)	**Page 7/**line 22 and **Page 8/**line 2
**Data processing** - Methods for processing data prior to and during analysis, including transcription, data entry, data management and security, verification of data integrity, data coding, and anonymization/de-identification of excerpts	**Page 7/**line 23-28 and **Page 8/**line 18-31
**Data analysis** - Process by which inferences, themes, etc., were identified and developed, including the researchers involved in data analysis; usually references a specific paradigm or approach; rationale**	**Page 8/**line 18-31 and line 37-44
**Techniques to enhance trustworthiness** - Techniques to enhance trustworthiness and credibility of data analysis (e.g., member checking, audit trail, triangulation); rationale**	**Page 7/**line 45-50 and **Page 8/**line 15-22

Results/findings

**Synthesis and interpretation** - Main findings (e.g., interpretations, inferences, and themes); might include development of a theory or model, or integration with prior research or theory	**Page 9/**line 2-11
**Links to empirical data** - Evidence (e.g., quotes, field notes, text excerpts, photographs) to substantiate analytic findings	**From Page 9/**line 13 **to Page 17/**line 3

Discussion

**Integration with prior work, implications, transferability, and contribution(s) to the field -** Short summary of main findings; explanation of how findings and conclusions connect to, support, elaborate on, or challenge conclusions of earlier scholarship; discussion of scope of application/generalizability; identification of unique contribution(s) to scholarship in a discipline or field	**From Page 18/**line 1 **to Page 19/**line 14
**Limitations** - Trustworthiness and limitations of findings	**Page 19/**line 16-33

Other

**Conflicts of interest** - Potential sources of influence or perceived influence on study conduct and conclusions; how these were managed	**Page 20/**line 19-20
**Funding** - Sources of funding and other support; role of funders in data collection, interpretation, and reporting	**Page 20/**line 22-26

*The authors created the SRQR by searching the literature to identify guidelines, reporting standards, and critical appraisal criteria for qualitative research; reviewing the reference lists of retrieved sources; and contacting experts to gain feedback. The SRQR aims to improve the transparency of all aspects of qualitative research by providing clear standards for reporting qualitative research.

For peer review only

**The rationale should briefly discuss the justification for choosing that theory, approach, method, or technique rather than other options available, the assumptions and limitations implicit in those choices, and how those choices influence study conclusions and transferability. As appropriate, the rationale for several items might be discussed together.

Reference:

O'Brien BC, Harris IB, Beckman TJ, Reed DA, Cook DA. **Standards for reporting qualitative research: a synthesis of recommendations.** *Academic Medicine*, Vol. 89, No. 9 / Sept 2014 DOI: 10.1097/ACM.0000000000000388

For peer review only
